# Spatial and Social Distance at the Onset of the Fertility Transition: Sweden, 1880–1900

**DOI:** 10.1007/s13524-018-0737-9

**Published:** 2019-01-17

**Authors:** Sebastian Klüsener, Martin Dribe, Francesco Scalone

**Affiliations:** 1 Max Planck Institute for Demographic Research, Konrad-Zuse-Str. 1, 18057 Rostock, Germany; 2 Federal Institute for Population Research, Wiesbaden, Germany; 3 Vytautas Magnus University, Kaunas, Lithuania; 4 Centre for Economic Demography and Department of Economic History, Lund University, Lund, Sweden; 5 Department of Statistical Sciences, University of Bologna, Bologna, Italy

**Keywords:** Fertility transition, Socioeconomic status, Geography, Sweden

## Abstract

Most studies on the fertility transition have focused either on macro-level trends or on micro-level patterns with limited geographic scope. Much less attention has been given to the interplay between individual characteristics and contextual conditions, including geographic location. Here we investigate the relevance of geography and socioeconomic status for understanding fertility variation in the initial phase of the Swedish fertility transition. We conduct spatially sensitive multilevel analyses on full-count individual-level census data. Our results show that the elite constituted the vanguard group in the fertility decline and that the shift in fertility behavior occurred quickly among them in virtually all parts of Sweden. Other socioeconomic status groups experienced the decline with some delay, and their decline patterns were more clustered around early centers of the decline. Long-distance migrants initially had higher fertility than people living close to their birthplace. However, as the fertility decline unfolded, this advantage was either reduced or reversed. This supports the view that migration and fertility are linked in this process. Our results confirm that socioeconomic status differences were of considerable relevance in structuring the fertility transition. The degree to which spatial distance fostered spatial variation in the fertility decline seems to have been negatively correlated with socioeconomic status, with the pattern of decline among the elite showing the lowest degree of spatial variation.

**Electronic supplementary material:**

The online version of this article (10.1007/s13524-018-0737-9) contains supplementary material, which is available to authorized users.

## Introduction

The decline in fertility during the demographic transition has long been a major theme in contemporary and historical demography. Many studies have focused on demographic aspects of this process, while other research has offered explanations for the transition, mainly at the macro level. Meanwhile, much less attention has been paid to disaggregated patterns and micro-level analyses. Conceptually, the fertility transition is often viewed within the frameworks of adjustment and innovation (Carlsson 1966). According to the *adjustment perspective*, fertility decline is a response to changes in the motivation to have children, which is connected to the demand for and the supply of children (Easterlin and Crimmins 1985). In pretransitional societies, the demand for children is high, but the supply is low because of high mortality; thus, demand exceeds supply. Fertility decline is explained by the adjustment to processes that influence the demand and/or the supply side. These developments include reductions in infant and child mortality (Galloway et al. 1998; Haines 1998; Reher 1999; Reher and Sanz-Gimeno 2007) and increasing costs of having children due to rising food and housing prices or due to government policies that limit child labor (see Alter 1992; Guinnane 2011; Schultz 2001). As a result of such trends, investing in the quality rather than the quantity of children becomes more appealing.

According to the *innovation perspective*, fertility decline is mainly the result of the spread of knowledge about and the increased social acceptance of using contraceptive techniques (Cleland and Wilson 1987; Coale and Watkins 1986). It is thus assumed that the emergence of deliberate birth control as a mass phenomenon involves the transmission of ideas and changing attitudes regarding the appropriateness of fertility control as well as the acquisition of knowledge about how to limit fertility. Many scholars believe that this knowledge existed among families long before the fertility transition. However, according to this point of view, such knowledge was used less for parity-specific birth control than for the spacing of births or the avoidance of childbearing in difficult times (Bengtsson and Dribe 2006; David and Sanderson 1986; Dribe and Scalone 2010; Santow 1995; Van Bavel 2004a).

Researchers have often stressed that the adjustment and the innovation perspectives are best viewed as complementary rather than as competing (e.g., Cleland 2001; Goldstein and Klüsener 2014; Lesthaeghe and Neels 2002; Palloni 2001). In both explanations, access to information plays an important role. To adapt to structural change, individuals need to become aware of these developments. They gain this awareness not only through their own life experiences but also through communication with others and information spread by the media (Bongaarts and Watkins 1996; Montgomery and Casterline 1996). Likewise, for ideas to diffuse in societies or for social norms related to proper family behavior to shift, individuals need to become aware of these ideas and shifts through similar pathways. Individuals vary in their access to information, and the exchange of information is moderated by spatial and social distance (see also Hägerstrand 1965; Szreter 1996). This likely affects the spatiotemporal patterns of fertility changes across the social strata during the fertility transition. Imperfect access to information combined with bias in risk perceptions (for the latter, see Montgomery 2000) can also produce temporal lags between the emergence of structural conditions that provide incentives for reducing the number of offspring and the actual adoption of such behavior (Van Bavel 2004b).

Growing empirical evidence shows that variation in access to information relevant for shifts in fertility behavior is an important component of the fertility transition, with spatial and social distance acting as moderators (Garrett et al. 2001; Goldstein and Klüsener 2014; González-Bailón and Murphy 2013; Junkka 2018; Schmertmann et al. 2010; Szreter 1996). However, little is known about the interplay between individual characteristics and contextual conditions, including geographic location. Relatively few analyses have attempted to differentiate the spatial patterns of fertility decline by socioeconomic status (SES). This study contributes to closing these knowledge gaps.

Earlier research on spatial or social differences in fertility during the fertility transition has shown that the initial phase in particular tends to be characterized by divergence (on spatial differences, see Basten et al. 2011; Watkins 1991; on social differences, see Bengtsson and Dribe 2014; Dribe et al. 2017a; Haines 1989; Jaadla et al. 2018). These findings suggest that spatial and social distance might be particularly important moderators in this phase. We thus make use of rich data to study the initial phase of the fertility transition in Sweden. Our aim is to investigate the relevance of the interplay of spatial distance and SES differences for understanding fertility variation. Toward this end, we examine how the emergence of low fertility in Sweden was clustered in specific locations and/or SES groups. In addition, we explore the possible determinants of fertility disparities by location and SES.

**Fig. 1:**
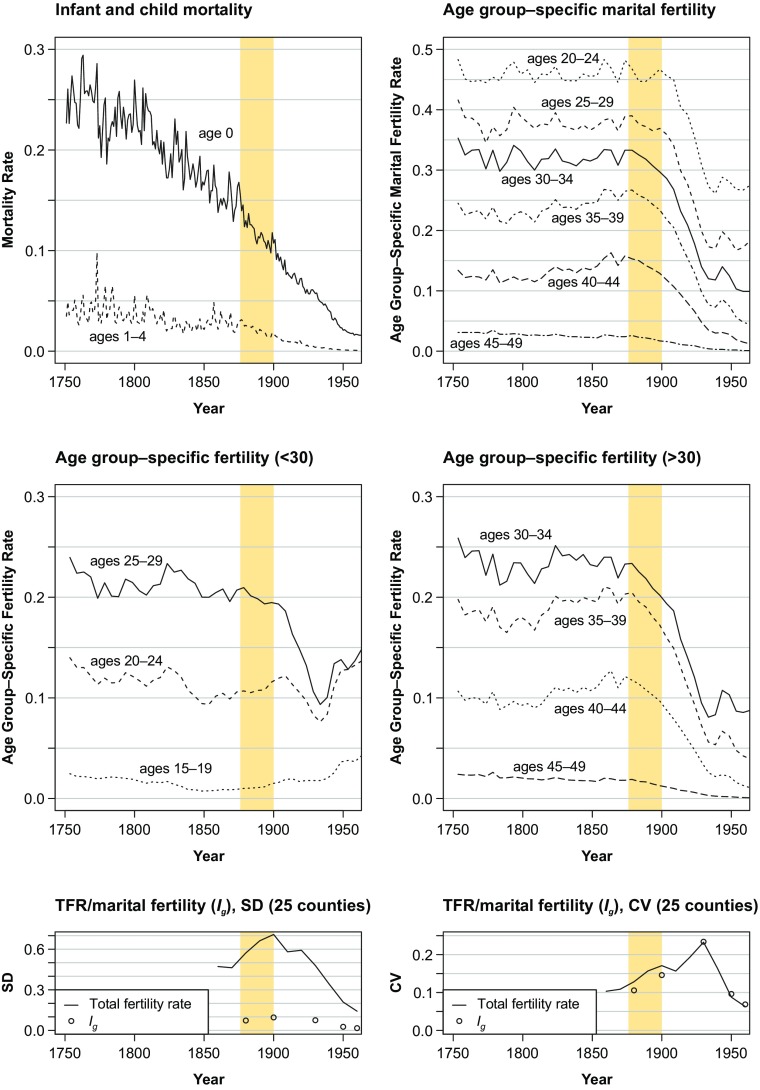
Trends in mortality below age 5 and fertility in Sweden (1750–1950). *I*_*g*_ is an index of marital fertility (Coale and Watkins 1986). The mortality rates are available for single years, the age group-specific fertility rates are averages for 5-year intervals, and the total fertility rate (TFR) data for the 25 counties are available for single years in 10-year intervals. The observation period for which we consider births (1876–1900) is highlighted. The coefficient of variance (CV) is derived by dividing the standard deviation (SD) by the mean. *Sources:* Human Mortality Database (2017), Statistics Sweden (1999), Coale and Watkins (1986), own calculations.

From full-count individual-level data from the censuses of 1880, 1890, and 1900, we derive information on children born between 1876 and 1900. We apply spatially sensitive multilevel models to investigate the role of spatial and socioeconomic dimensions in net fertility variation by accounting for various individual-level and parish-level characteristics. Figure 1 demonstrates which part of the demographic transition in Sweden our analysis covers. The figure displays trends in mortality below age 5 and fertility between 1750 and 1950. Although infant and child mortality were declining throughout the nineteenth century, the fertility transition did not gain momentum until the last quarter of the century. During our observation period, fertility decline was mostly concentrated among mothers aged 30 and older (Dribe 2009). The subplots at the bottom of Fig. 1 show long-term trends in (marital) fertility variation across the 25 Swedish counties. They illustrate that our study period was indeed characterized by a substantial divergence in regional fertility variation.

## Theoretical Considerations

According to Coale (1973), three preconditions are needed for a fertility transition to occur. First, couples have to be consciously aware that reducing their number of offspring is beneficial for them (*readiness*). Second, the new behavior must be culturally accepted (*willingness*). Third, the technical means for preventing conception and the knowledge of those means must be available (*ability*). Although scholars have debated whether these preconditions are universally applicable (Eckstein and Hinde 2002), they are helpful for developing a theoretical understanding of how the interplay of spatial distance and social differences between individuals might have contributed to fertility disparities across locations and SES groups during the fertility transition.

At the onset of the fertility transition in Sweden, the benefits of reducing fertility were perhaps most evident in large cities, where individuals were confronted with rapid social changes. These brought about new social mobility opportunities, but they also contributed to rising costs of living. Urban inhabitants were probably not only more ready but also more willing to change their behavior: cities generally provide greater anonymity than nonurban areas. Thus, compared with their rural counterparts, city dwellers faced a lower risk of losing social capital (Bourdieu and Wacquant 1992) by adopting a new, potentially norm-deviating behavior. Social capital losses might have also had financial implications if they affected employment opportunities or transfers from family members (e.g., inheritances). Another reason why residents of large cities were likely forerunners in the decline is that cities were central nodes of communication and transportation networks. This made cities important hubs for the diffusion of information on changes in structural conditions, shifts in social norms, and new ideas and technologies (Simon and Nardinelli 1996).

Because most social interactions with information exchange were still local during our study period, we believe that spatial distance was an important moderator in the fertility decline. Early adopters in a given location likely faced uncertainties about how their local social network would perceive their behavior. Thus, we expect that the adoption rates were initially most intense in localities where pioneers had already shifted their behavior without being subjected to substantial sanctions and in areas adjacent to these early centers of fertility decline. This chain of events can cause spatial fertility decline patterns in the form of nebula-like clusters (Hägerstrand 1965), in which early centers constitute cores with high adoption rates that are surrounded by areas in which the risk of early adoption progressively decreases by distance. However, the emergence of such patterns is not necessarily attributable to an information diffusion process; they might have also been caused by an adaptation to structural changes with a spatial dimension (e.g., increases in income in cities might have positively affected incomes in surrounding areas).

In addition to spatial distance, social distance may have constrained the spread of behavioral shifts (Matthys 2013; Rogers 2003; Rosero-Bixby and Casterline 1993; Skirbekk 2008). The elite have frequently been identified as a vanguard group during the fertility transition (Dribe and Scalone 2014; Dribe et al. 2014, 2017b; Haines 1992; Livi-Bacci 1986). They might have been more ready than their less-elite counterparts to change their fertility behavior because new social mobility opportunities were emerging for the higher social strata in particular at that time (Dribe et al. 2015). It has also been argued that declining fertility among the higher social strata can have self-reinforcing effects given that it can influence views on ideal family sizes (Skirbekk 2008). Swedish society in the late nineteenth century was highly stratified, with nobility, high-level managers, and professionals forming a distinctive elite into which mobility was limited (see, e.g., Clark 2014; Dribe et al. 2015). Thus, such norm shifts were more likely to first concern other elite member than to trickle down to other SES groups (see also Matthys 2013).

But elite groups also differed in their access to information: at least in historical contexts, they were much more likely than less-advantaged groups to maintain social networks across long distances. Szreter (1996) presented evidence in support of a higher social connectedness through space for Britain. He showed that elites of the same profession had very similar fertility trends during the fertility decline, even if they lived far apart. Based on his findings, he argued that recognizing the existence of *communication communities* of similar social backgrounds is important for understanding the mechanisms of the fertility decline.

The elite also likely differed from others in their access to assets and in their level of *local social embeddedness*, which we define as the degree to which their social capital depended on their social relationships in the local area.^1^ These conditions might have affected what Coale referred to as “willingness.” Quite a few elite couples had moved from urban centers to nonmetropolitan areas so that the husband could take a position as, for example, a doctor, clergyman, or local administrator. Because of such moves, a considerable share of Stockholm-born elite women were living in remote areas of Sweden (see Klüsener et al. 2017). It seems likely that these women were still following new developments in Stockholm, including shifts in fertility behavior. Elite women also might have been less embedded in local social control networks (Lesthaeghe 1980) for several reasons. First, many elite women were not living close to their place of birth (see upcoming Analytical Strategy section), and may therefore have been less subject to control by other family members (see also Creighton et al. 2012). The situation was very different for women in farm families, whose lives were generally more focused on the area in which they were born (Klüsener et al. 2017). Second, being part of the elite gave these women a distinctive identity that may have made it easier to risk adopting a new deviant behavior, even if the reaction of local social contacts was uncertain.

Based on our theoretical considerations, we formulate the following expectations. If we observe that the fertility decline in this early phase of the fertility transition in Sweden clustered around early centers of the decline, with the clusters persisting even after SES variation is controlled for, we will interpret this finding as evidence that geography was an important moderator in the transition. If we see different adoption rates by SES, independent of whether individuals were living in central or peripheral locations, we will interpret this observation as an indicator that SES disparities were an important moderator of the process. Based on the assumption that access to information on aspects such as structural changes, shifts in social norms, and new ideas is relevant for reducing the number of offspring, we expect that SES groups and locations with better access to such information were forerunners in the fertility decline.

## Data and Methods

We analyze micro-level data from the Swedish censuses of 1880, 1890, and 1900 (Swedish National Archives et al. 2011a, 2011b, 2014). In 1880, approximately 4.6 million persons in 1.2 million households were counted, compared with 4.8 million persons and 1.3 million households in 1890 and 5.2 million persons and 1.4 million households in 1900. These data, digitized by the Swedish National Archives, are freely available for scientific use through the North Atlantic Population Project (Ruggles et al. 2011). All persons are grouped by household. The individual-level attributes include information on the sex, age, marital status, occupation, household headship, parish of residence, and parish of birth. Family pointer variables indicate within the household the mother, the father, or the spouse, allowing us to link each woman to her own children and husband.

We control for socioeconomic contextual conditions at the parish level based on aggregated census data and derived spatial distances to large urban centers. In preparing our analysis, we accounted for changes in the parish boundaries in the period of observation. This resulted in 2,435 time-constant parishes for the 1880–1900 period (see Fig. OA1 in the online appendix, section 1). Our data set was then linked to a historical GIS file of administrative boundaries (Riksarkivet 2016).

### Dependent Variable

The census data do not permit us to compute fertility rates. We therefore apply the child-woman ratio (CWR), an indirect fertility measure traditionally defined as the number of children aged 0–4 per woman aged 15–49 (Shyrock and Siegel 1980). We use this measure at the individual level, which implies that children under age 5 reported in the census would have been born during the 5-year period before the census date, when the mother was up to five years younger. Given that we focus on marital fertility, we created for each of the three censuses a sample of married women aged 15–54 to cover all surviving children aged 0–4 who were born to women aged 15–49. This sample is limited to married women whose spouses were present because we derive SES information from these spouses. Hence, our focus is on women who had a partner with whom they could potentially conceive children. Descriptive statistics on the number of children per woman by SES are provided in our online appendix (section 2).

Focusing on marital fertility is also an advantage given that Sweden experienced substantial emigration during our observation period, with emigration levels varying both across regions and by SES. We cannot control for emigration directly, but previous research suggests that our analysis is unlikely to be substantially affected by emigration given that most emigrants were unmarried young adults (Bohlin and Eurenius 2010). In addition, the observed marital fertility decline was primarily concentrated among women aged 30 and older, who had low emigration rates (see the online appendix, section 3).

Using a net fertility measure raises the question of whether our analyses would have provided different results if we had been able to study gross fertility because trends and variation in net fertility can be affected by trends and variation in infant and child mortality. In the online appendix (section 4), we provide analyses related to this question. They suggest that the influence of infant and child mortality improvements on the CWR was particularly small in the second half of our observation period. Additional regional and SES comparisons show that fertility and net fertility were highly correlated, which reassures us that the outcomes of an analysis of fertility rates would not have been substantially different (Scalone and Dribe 2017). Net fertility might also be a more informative measure because we expect that families cared more about their surviving children than about all children ever born.

### Measuring Socioeconomic Status (SES)

During our study period, the husband was usually the main breadwinner in the family, and there was seldom an occupational notation for a married woman in the census. Hence, we measure SES based on the husband’s occupation. The census data offer detailed occupation information, which we categorized by occupational group according to the Historical International Standard Classification of Occupations (HISCO; van Leeuwen et al. 2002). These occupational groups were then aggregated into 12 SES groups by applying the HISCLASS scheme (van Leeuwen and Maas 2011), which takes into account skill level, degree of supervision, type of work (manual vs. nonmanual), and urban versus rural residence. The 12 classes are as follows: (1) higher managers; (2) higher professionals; (3) lower managers; (4) lower professionals/clerical and sales personnel; (5) lower clerical and sales personnel; (6) foremen; (7) medium-skilled workers; (8) farmers and fishermen; (9) lower-skilled workers; (10) lower-skilled farm workers; (11) unskilled workers; and (12) unskilled farm workers.

To address challenges associated with small sample sizes in some classes, we further aggregated the 12 classes into six or three SES groups, depending on the type of analysis. The six groups, which we use in some descriptive analyses and as SES controls in our models, are as follows: the elite and upper–middle class (HISCLASS 1–6), farmers (HISCLASS 8), skilled workers (HISCLASS 7), lower-skilled workers (HISCLASS 9–10), unskilled workers (HISCLASS 11–12), and individuals who could not be allocated to a specific class (others). The three further aggregated groups, for which we map the fertility decline and run separate models, are as follows: the elite (HISCLASS 1–6), farmers (HISCLASS 8), and workers and others (HISCLASS 7, 9–12, others). Szreter (1996) argued that social classes might be too broad for carving out social disparities in the fertility transition. However, a reevaluation of his analyses of Britain with more sophisticated methods has shown that broad social classes allow capturing large parts of the social variation in the fertility decline (Barnes and Guinnane 2012). This reassures us about our decision to perform analyses for broad SES groups.

### Analytical Strategy

One challenge is that although the fertility decline was unfolding dynamically in space and time, our detailed data come in a cross-sectional form that allow insight into net fertility only at specific times during the fertility transition. Therefore, we cannot directly identify individual and contextual factors that shaped fertility changes. Instead, we investigate how statistical associations between net fertility and individual- and parish-level characteristics shifted during the fertility transition. We are, however, able to map the spatiotemporal fertility dynamics by SES in the first descriptive part of our analysis. To our knowledge, this is the first time that geographically detailed maps on the fertility transition by SES are presented for an entire country.

In the second part of our analysis, we run separate regression models for 1880, 1890, and 1900 to estimate the associations between net fertility and SES and other individual- and parish-level characteristics.^2^ The models are based on a multilevel approach with parish-level random intercepts. In addition to running models that include all women independent of SES, we estimate separate models for each of the three big SES groups (the elite, farmers, and workers and others). The estimation equation is as follows:

$$ {y}_{wi}=\upalpha +{\upzeta}_i+\sum \limits_{l=1}^L\;{\upbeta}_l{\mathbf{X}}_{l, wi}+\sum \limits_{k=1}^K\;{\upbeta}_k{\mathbf{Z}}_{k,i}+{\upvarepsilon}_{wi}, $$

where the dependent variable *y* is the number of children aged 0–4 of a woman *w* in parish *i*, α is an intercept, and ζ is a random effect for each parish *i*. **X** represents a vector of individual-level covariates, **Z** is a vector of parish-level covariates, and ε is the error term.

Because we run regression models on geographically highly detailed data, spatial autocorrelation might introduce bias due to the violation of regression assumptions. One important assumption is that the observations are independent. This assumption is often violated in spatial models, given that adjacent units are likely to share many characteristics. If the models are unable to control for the factors that cause this so-called positive spatial autocorrelation, residuals with high or low values will be spatially clustered. Positive spatial autocorrelation might bias parameter estimates and deflate standard errors downward, with the latter resulting in overly optimistic statistical significance tests (Anselin 1988).

To explore whether our models might be affected by spatial autocorrelation, we derive the Moran’s I index^3^ of spatial autocorrelation (Moran 1950) for the parish-level mean values of the dependent variable and the model residuals. The Moran’s I is very similar to Pearson’s correlation coefficient except that instead of looking for the correlation between the values of two variables *x* and *y* in each parish *i*, it examines the correlation between the values of a variable *y* in each parish *i* with information on the values of the same variable *y* in the parishes *j* that are adjacent to parish *i*. The Moran’s I can take values ranging from –1 (strong negative spatial autocorrelation) to 1 (strong positive spatial autocorrelation). The closer it is to 0, the less we are faced with spatial autocorrelation. Our Moran’s I tests use spatial weight matrices that define the five nearest parishes *j* as neighbors, giving each neighbor equal weight.^4^ We also implemented sensitivity checks with alternative spatial weight matrices (see the online appendix, section 5), which generally provided very similar patterns. In addition to estimating our main models, we conducted sensitivity checks to investigate whether the main outcomes of our models hold if the remaining unaccounted spatial autocorrelation in the models is integrated into the random effects (see the online appendix, section 6). For this, we estimated conditional autoregressive models (Besag et al. 1991), using integrated nested Laplace approximation (INLA) based on Bayesian inference (Bivand et al. 2015; Martins et al. 2013).

Table 1 shows descriptive statistics for our covariates. These include controls for SES, woman’s age, and the age difference between spouses. The dummy variable indicating whether children over age 4 are linked to the mother can be viewed as an indirect measure of marital duration: that is, that the couple had the option of having children for the entire 5-year period preceding the census.^5^ We control for whether the husband was the household head because we expect that women whose husbands were not heading the household had lower fertility due to a more restricted access to resources.

**Table 1:** Distribution of covariates (%)

	1880	1890	1900
Individual-Level Covariates			
Woman’s age			
15–19	0.4	0.4	0.4
20–24	6.0	5.4	6.5
25–29	13.5	14.1	13.6
30–34	16.8	17.9	15.9
35–39	17.8	17.3	18.2
40–44	16.2	16.2	17.4
45–49	15.7	15.4	15.0
50–54	13.7	13.2	13.0
Age difference between spouses			
Wife older	27.9	26.9	26.0
Husband 0–2 years older	21.3	22.0	22.7
Husband 3–6 years older	25.2	25.6	26.3
Husband >6 years older	25.6	25.6	24.9
Children >4 years old in household			
No	30.9	29.9	29.6
Yes	69.1	70.1	70.4
Husband household head			
Yes	96.0	96.2	96.9
No	4.0	3.8	3.1
Socioeconomic status			
Elite	10.2	11.9	14.0
Farmers	41.2	37.5	32.4
Skilled workers	9.4	11.2	13.0
Lower-skilled workers	8.2	10.8	13.7
Unskilled workers	24.2	23.1	21.7
Others	6.9	5.5	5.1
Distance from parish of birth			
Less than 10 km	57.8	53.1	48.4
10–50 km	28.8	29.5	30.2
More than 50 km	12.8	16.6	20.4
Born abroad	0.6	0.8	1.0
Parish-Level Covariates			
Female labor force rate			
Low (1st quartile)	25.3 [<28.6]	24.1 [<28.6]	22.0 [<26.1]
Medium (2nd and 3rd quartiles)	48.0 [28.6–45.0]	47.2 [28.6–45.4]	45.1 [26.1–42.4]
High (4th quartile)	26.7 [>45.0]	28.8 [>45.4]	32.9 [>42.4]
Education rate (teacher/child ratio)			
Low (1st quartile)	21.6 [<0.1]	22.3 [<0.7]	22.9 [<1.1]
Medium (2nd and 3rd quartiles)	59.3 [0.1–1.2]	55.4 [0.7–2.0]	58.9 [1.1–2.4]
High (4th quartile)	19.2 [>1.2]	22.3 [>2.0]	18.2 [>2.4]
Proportion employed in industry			
Low (1st quartile)	19.3 [<4.1]	17.5 [<5.2]	15.9 [<6.1]
Medium (2nd and 3rd quartiles)	43.9 [4.1–11.7]	40.3 [5.2–14.0]	36.6 [6.1–17.1]
High (4th quartile)	36.8 [>11.7]	42.2 [>14.0]	47.5 [>17.1]
Proportion of migrants born more than 100 km away and/or abroad			
Low (1st quartile)	17.2 [<0.8]	14.6 [<0.9]	13.1 [<1.4]
Medium (2nd and 3rd quartiles)	47.0 [0.8–3.8]	44.5 [0.9–4.5]	40.0 [1.4–5.3]
High (4th quartile)	35.8 [>3.8]	41.0 [>4.5]	46.9 [>5.3]
Population density per km^2^			
Less than 50	76.4	71.6	67.1
50–100	9.3	8.4	8.4
100–1,000	7.3	9.8	12.2
More than 1,000	7.1	10.3	12.2
Regional dummy variable			
Less than 10 km from Stockholm	3.4	5.2	5.9
10–50 km from Stockholm	2.2	2.2	2.3
50–100 km from Stockholm	5.6	5.8	5.6
100–150 km from Stockholm	7.2	7.4	7.5
150–200 km from Stockholm	9.2	8.8	9.1
Less than 10 km from Gothenburg	2.0	2.8	3.2
10–50 km from Gothenburg	2.7	2.5	2.3
50–100 km from Gothenburg	7.2	6.5	6.3
Less than 10 km from Malmö	1.3	1.5	1.9
10–50 km from Malmö	4.9	4.6	4.6
50–100 km from Malmö	6.1	6.1	5.8
Gotland	1.3	1.2	1.1
Southern Norrland and Kopparberg county	11.6	12.9	12.8
Northern Norrland	4.3	4.9	5.6
Other areas (central and southern Sweden)	30.9	27.9	25.9
Number of Women	580,849	586,918	619,096
Number of Parishes	2,435	2,435	2,435

*Note:* For the parish-level indicators, which are introduced by quartiles, we provide the category bins in brackets.

*Sources:* Swedish National Archives et al. (2011a, 2011b, 2014), own calculations.

One of the innovative elements of our study is that we account for the lifetime net migration background (i.e., the distance between the parish of birth and the parish of residence).^6^ A conceptual challenge we face is that this measure could be a proxy for several aspects. As discussed earlier, we believe that it could be a proxy for social connectedness through space and local social embeddedness. Migrants living far from their birthplace might have had better access to nonlocal sources of information (Szreter 1996) given that they frequently maintained contact with family and friends in their former places of residence (Creighton et al. 2012). In addition, they may have been less embedded in local social and family control networks. These aspects might help to explain why migrants from low-fertility to high-fertility contexts tended to exhibit fertility patterns similar to those of their region of origin (see, e.g., Van Bavel 2004b). However, long-distance migrants might have also been selected for their ambition and risk tolerance and may therefore have been more receptive to changes in conditions that made shifting from quantity to quality investments in children appealing (Creighton et al. 2012). Because long-distance migrants were heavily clustered in densely populated locations, migrants in these contexts may have had greater incentives to reduce their number of offspring because they may have been less able to rely on their own property or local family networks of support (Creighton et al. 2012; Puschmann et al. 2014). All these factors may have increased the likelihood that long-distance migrants were among the pioneers of the process.

**Fig. 2:**
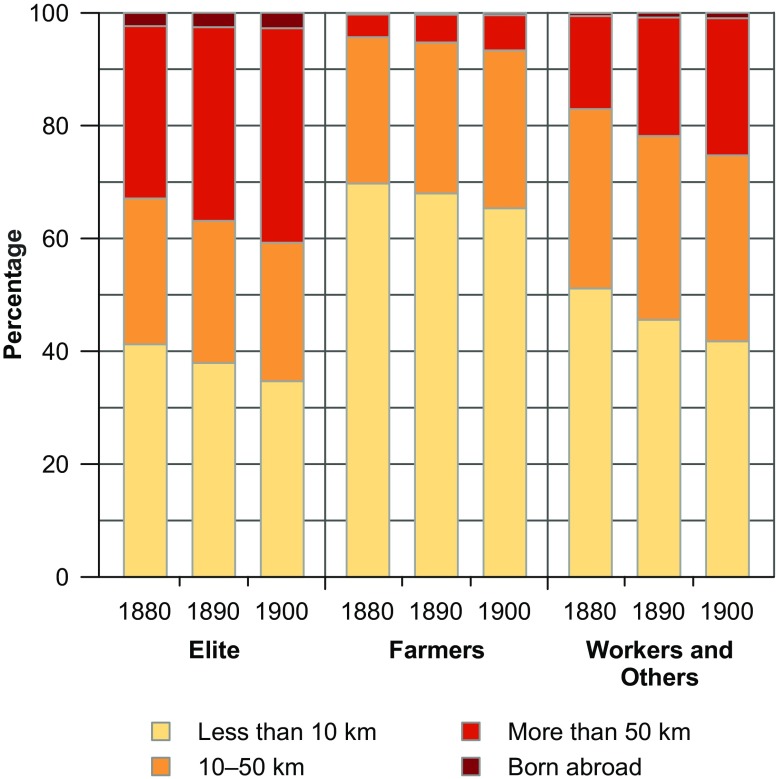
Lifetime net migration pattern by socioeconomic status (women aged 15–54). For individuals born in Sweden, migration is measured by calculating the spherical distances between the parish of birth and the parish of residence. *Sources:* Swedish National Archives et al. (2011a, 2011b, 2014), own calculations.

Figure 2 displays trends in lifetime net migration by SES. It demonstrates that the share of long-distance migrants was particularly high among the elite, whereas most women in farm families lived very local lives. We include lifetime net migration information at both the individual and the contextual levels because we believe that living in a parish with a large share of long-distance migrants may have also been relevant for early shifts in fertility behavior among locally born women (see Van Bavel 2004b). This pattern could again be related to various mechanisms. High levels of in-migration might have caused housing markets to become tighter, and may thus have provided greater structural incentives for a behavioral shift. Alternatively, local people may have also benefited from the better social connectedness of these in-migration locations to the locations the in-migrants had left, where the latter may have still had social contacts (Creighton et al. 2012). In our lifetime net migration variables, women who were born abroad are treated separately because we lack information on their parish of birth. For the contextual parish-level variable, we use the proportion of migrants who were born more than 100 km away or abroad.

Other parish-level covariates that capture socioeconomic conditions include female labor force participation, educational orientation, degree of industrialization, and population density. The latter also serves as a proxy for urbanization. We assume that all four variables are negatively related to fertility during the transition (see, e.g., Dribe 2009; Fox et al. 2018; Galloway et al. 1994; Goldstein and Klüsener 2014). In examining female labor force participation, we focus on never-married women aged 15–64 because their labor market status was less likely affected by prior births. Educational orientation is measured by the number of teachers in basic education per 100 children of school age (aged 7–14). The degree of industrialization is based on the HISCO–coded occupations and is calculated for males aged 15–64. Ideally, we would have also included information on infant mortality. Unfortunately, however, these data were not available at the parish level for this period.

Finally, we use a regional dummy variable to capture the regional variation that remains unexplained by the other covariates. Its main purpose is to determine the degree to which the unexplained fertility decline exhibits a nebula-like cluster around major cities. Thus, it covers categories with parishes within specific distances of the three biggest cities in Sweden: Stockholm, Gothenburg, and Malmö. Next, to reduce the bias introduced by spatial autocorrelation in the model estimates, we include controls for regions with specific fertility levels that cannot be explained with our models.

## Results

### Descriptive Findings

To investigate the spatiotemporal fertility decline patterns by SES in this initial phase of the fertility transition, we use age-standardized CWRs based on the age structure of all married women aged 15–54 in the 1890 census.^7^ The unstandardized and standardized CWRs by SES for the three censuses are presented in Table 2. Net fertility was rather stable between 1880 and 1890, and then decreased by 3 % between 1890 and 1900. However, these trends differed substantially by SES. The elite SES group already had a below-average CWR in 1880 and experienced a decline of approximately 13 % between 1880 and 1900. Farmers, on the other hand, had an above-average CWR in 1880 and experienced a slight increase over the subsequent decades. As a result, the gap between the CWRs of the elite and the farmers increased from 0.08 to 0.20. Skilled and lower-skilled workers experienced CWR declines of 7 % and 4 %, respectively; unlike among the elite, however, these declines occurred almost entirely in the second part of our study period.

**Table 2:** Child-woman ratio (CWR) by socioeconomic status

	Not Age-Standardized			Age-Standardized		
	1880	1890	1900	1880	1890	1900
Socioeconomic Status						
Elite	0.87	0.82	0.73	0.84	0.80	0.73
Farmers	0.85	0.85	0.83	0.92	0.92	0.93
Skilled workers	0.93	0.93	0.87	0.90	0.89	0.84
Lower-skilled workers	1.00	1.02	0.97	0.93	0.93	0.89
Unskilled workers	0.89	0.94	0.91	0.87	0.89	0.86
Others	0.75	0.73	0.74	0.80	0.78	0.77
Total	0.87	0.89	0.85	0.89	0.89	0.86

*Sources:* Swedish National Archives et al. (2011a, 2011b, 2014), own calculations.

**Fig. 3:**
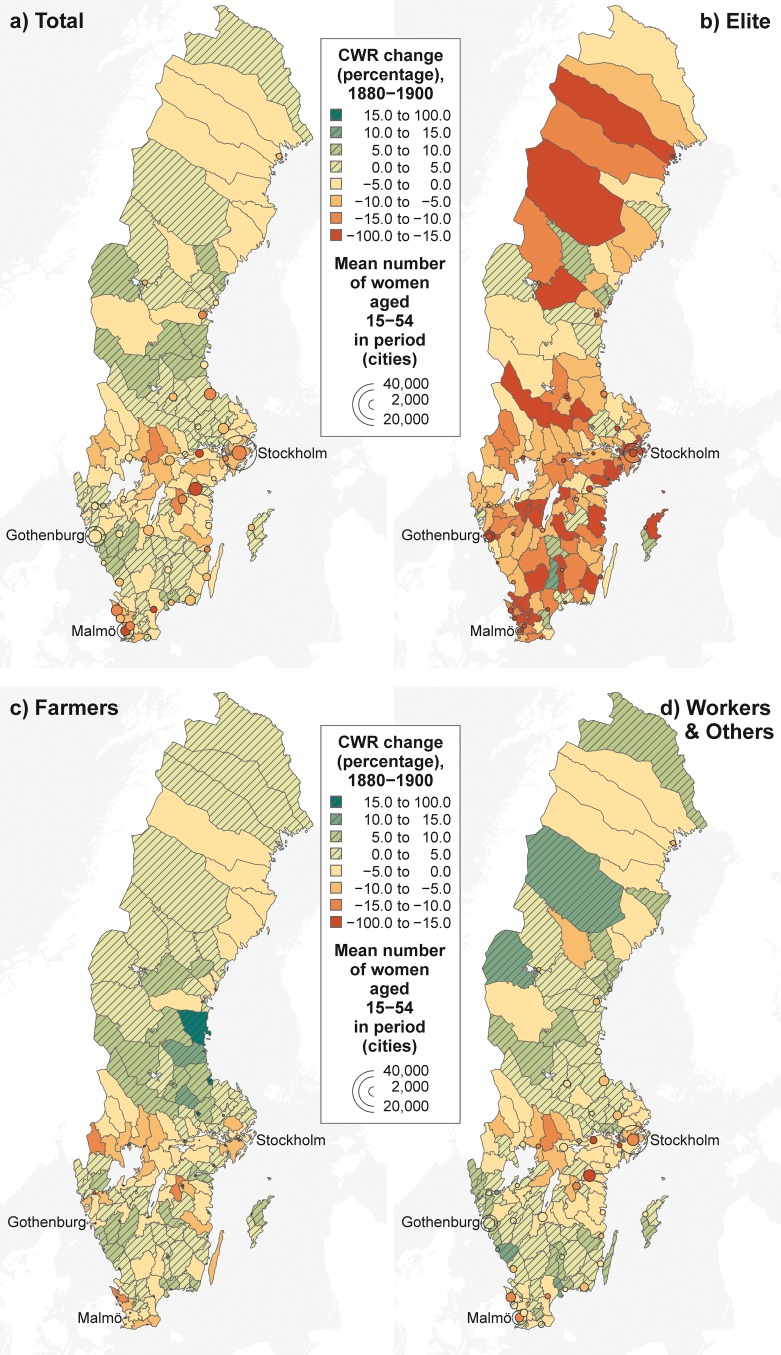
Percentage change in the child-woman ratio (CWR) by socioeconomic status: 159 Swedish judicial districts (1880–1900). Although our models consider the spatially more detailed parish level, these maps are based on data at the level of the judicial districts (see text for motivation). The CWRs have been age-standardized using the age structure of the total married female population aged 15–54 in 1890 as a reference. Cities that formed their own judicial districts and had more than 5,000 inhabitants in 1900 are highlighted with circles that vary proportionally by the number of women aged 15–54. For the three biggest cities, we leave the outer part of the circle transparent. *Sources:* Swedish National Archives et al. (2011a, 2011b, 2014), own calculations. *Base Maps:* Riksarkivet (2016), MPIDR (2014).

The vanguard role of the elite might be related in part to their concentration in big cities, which often constituted early fertility decline centers. The maps in Fig. 3 allow us to investigate this possibility. They show the percentage CWR change between 1880 and 1900 in the 159 judicial districts (*domsagas*) for all women and our three SES groups. We map the patterns at this higher administrative level to reduce random noise due to small local sample sizes.^8^ To some extent, the decline pattern for all women (Fig. 3, panel a) resembles a nebula-like cluster, which is very typical for cartographic representations of the fertility decline (Goldstein and Klüsener 2014; Schmertmann et al. 2008). The highest decline occurred in and next to large centers, such as Stockholm and Malmö,^9^ and in important transport and communication corridors. These corridors include the lake area in central Sweden between Stockholm and Gothenburg. An exception to this general pattern is Gothenburg (the second-largest city) and the surrounding territories, where the fertility decline in this period was more limited. We return to this issue in the discussion.

When we disaggregate the numbers by our three SES groups (Fig. 3, panels b–d), we see that the nebula-like clustering is also visible in the patterns of the farmers and workers and others, whereas the spatial pattern of the elite looks very different. In almost all areas of Sweden, regardless of whether the area was remote or central, the elite experienced a decline. The decline pattern among the elite suggests that information about the advantages of reducing fertility and contraceptive technologies had already spread to virtually all parts of Sweden during that period.

The spatial fertility change pattern of the farmers closely resembles the pattern for all social groups. This finding is not surprising given that farmers were the predominant SES group in rural areas, which covered most of Sweden at that time. Our third and most heterogeneous group—workers and others—also displays the most heterogeneous spatial pattern. In many areas of Sweden, their CWR was still increasing between 1880 and 1900. This was also the case in Stockholm and Gothenburg in the 1880–1890 period. On the other hand, we find distinct local urban hot spots of fertility decline. These include Norrköping, an industrial city southwest of Stockholm in which many women worked in textile production. In Norrköping, workers and others experienced a fertility decline of 16 % between 1880 and 1900. Overall, the maps in Fig. 3 show that in this early phase of the fertility transition in Sweden, fertility trends differed substantially by SES. In addition, trends varied greatly across locations within Sweden, especially among farmers and workers and others.

### Model Results

Table 3 displays the model results for all SES groups, while Table 4 shows the separate outcomes for our three SES groups (the elite, farmers, and workers and others). The Moran’s I diagnostics demonstrate that all 12 models exhibit positive spatial autocorrelation in the dependent variable. As is visible in the Moran’s I tests on the residuals, our models explain a substantial part of this spatial autocorrelation. However, in all but the models for the elite SES group, some unexplained spatial autocorrelation remains. We return to this point at the end of this section.

**Table 3:** Model estimates for the number of children aged 0–4 per married women aged 15–54

	1880		1890		1900	
	Coef.	*p* Value	Coef.	*p* Value	Coef.	*p* Value
Individual-Level Covariates						
Woman’s age						
15–19	–0.605	.000	–0.590	.000	–0.477	.000
20–24	–0.214	.000	–0.150	.000	–0.101	.000
25–29	0.054	.000	0.079	.000	0.120	.000
30–34 (ref.)						
35–39	–0.207	.000	–0.218	.000	–0.221	.000
40–44	–0.571	.000	–0.584	.000	–0.576	.000
45–49	–1.116	.000	–1.131	.000	–1.100	.000
50–54	–1.404	.000	–1.397	.000	–1.353	.000
Age difference between spouses						
Wife older	0.026	.000	0.028	.000	0.040	.000
Husband 0–2 years older (ref.)						
Husband 3–6 years older	–0.017	.000	–0.027	.000	–0.019	.000
Husband >6 years older	–0.083	.000	–0.102	.000	–0.083	.000
Children >4 years old in household						
No (ref.)						
Yes	0.252	.000	0.270	.000	0.252	.000
Husband household head						
Yes (ref.)						
No	–0.153	.000	–0.146	.000	–0.157	.000
Socioeconomic status						
Elite (ref.)						
Farmers	0.011	.005	0.050	.000	0.087	.000
Skilled workers	0.050	.000	0.086	.000	0.096	.000
Lower-skilled workers	0.060	.000	0.097	.000	0.115	.000
Unskilled workers	0.005	.162	0.060	.000	0.091	.000
Others	–0.022	.000	–0.002	.750	0.035	.000
Distance from parish of birth						
Less than 10 km (ref.)						
10–50 km	0.024	.000	0.028	.000	0.028	.000
More than 50 km	0.048	.000	0.034	.000	0.015	.000
Born abroad	0.015	.249	–0.017	.132	–0.029	.004
Parish-Level Covariates						
Female labor force rate						
Low (1st quartile)	–0.006	.181	–0.004	.348	0.010	.016
Medium (2nd and 3rd quartiles) (ref.)						
High (4th quartile)	–0.006	.166	–0.009	.047	–0.014	.002
Education rate (teacher/child ratio)						
Low (1st quartile)	–0.010	.010	0.008	.058	0.005	.200
Medium (2nd and 3rd quartiles) (ref.)						
High (4th quartile)	–0.011	.011	–0.011	.012	–0.027	.000
Proportion employed in industry						
Low (1st quartile)	0.021	.000	0.017	.000	0.016	.001
Medium (2nd and 3rd quartiles) (ref.)						
High (4th quartile)	0.001	.793	–0.009	.041	–0.005	.229
Proportion of migrants born more than 100 km away and/or abroad						
Low (1st quartile)	0.001	.823	0.001	.895	–0.003	.488
Medium (2nd and 3rd quartiles) (ref.)						
High (4th quartile)	–0.004	.470	–0.001	.913	–0.009	.048
Population density per km^2^						
Less than 50 (ref.)						
50–100	–0.027	.000	–0.021	.002	–0.035	.000
100–1,000	–0.057	.000	–0.049	.000	–0.058	.000
More than 1,000	–0.066	.002	–0.080	.000	–0.104	.000
Regional dummy variable						
Less than 10 km from Stockholm (ref.)						
10–50 km from Stockholm	0.034	.357	0.023	.516	0.071	.035
50–100 km from Stockholm	–0.003	.938	–0.026	.440	0.053	.109
100–150 km from Stockholm	0.014	.692	–0.003	.933	0.070	.034
150–200 km from Stockholm	0.084	.022	0.052	.127	0.110	.001
Less than 10 km from Gothenburg	0.163	.000	0.169	.000	0.253	.000
10–50 km from Gothenburg	0.165	.000	0.175	.000	0.246	.000
50–100 km from Gothenburg	0.165	.000	0.152	.000	0.248	.000
Less than 10 km from Malmö	0.156	.000	0.076	.054	0.194	.000
10–50 km from Malmö	0.112	.002	0.045	.194	0.157	.000
50–100 km from Malmö	0.136	.000	0.093	.007	0.171	.000
Gotland	–0.114	.003	–0.130	.000	–0.016	.645
Southern Norrland and Kopparberg county	0.095	.009	0.081	.019	0.166	.000
Northern Norrland	0.259	.000	0.216	.000	0.297	.000
Other areas (central and southern Sweden)	0.165	.000	0.132	.000	0.217	.000
Constant	1.101	.000	1.086	.000	0.953	.000
Number of Women	580,849		586,198		619,096	
Number of Parishes	2,435		2,435		2,435	
Spatial Autocorrelation Diagnostics						
Moran’s I dependent variable	0.465	.000	0.471	.000	0.414	.000
Moran’s I residuals	0.194	.000	0.162	.000	0.142	.000

*Notes:* The Moran’s I is derived at the parish level; the neighborhood is defined as the five nearest neighbors, with each neighbor given equal weight. Moran’s I tests for alternative spatial weight matrix specifications are presented in the online appendix (section 5).

*Sources:* Swedish National Archives et al. (2011a, 2011b, 2014), own calculations.

**Table 4:** Models by socioeconomic status: Estimates for the number of children aged 0–4 per married women aged 15–54

	Elite						Farmers						Workers and Others					
	1880		1890		1900		1880		1890		1900		1880		1890		1900	
	Coef.	*p* Value	Coef.	*p* Value	Coef.	*p* Value	Coef.	*p* Value	Coef.	*p* Value	Coef.	*p* Value	Coef.	*p* Value	Coef.	*p* Value	Coef.	*p* Value
Individual-Level Covariates																		
Woman’s age																		
15–19	–0.618	.000	–0.674	.000	–0.392	.000	–0.740	.000	–0.713	.000	–0.645	.000	–0.542	.000	–0.541	.000	–0.444	.000
20–24	–0.234	.000	–0.138	.000	–0.126	.000	–0.216	.000	–0.163	.000	–0.142	.000	–0.205	.000	–0.143	.000	–0.075	.000
25–29	0.075	.000	0.101	.000	0.123	.000	0.071	.000	0.094	.000	0.123	.000	0.043	.000	0.072	.000	0.125	.000
30–34 (ref.)																		
35–39	–0.250	.000	–0.262	.000	–0.240	.000	–0.217	.000	–0.223	.000	–0.226	.000	–0.191	.000	–0.207	.000	–0.218	.000
40–44	–0.651	.000	–0.640	.000	–0.608	.000	–0.578	.000	–0.599	.000	–0.602	.000	–0.552	.000	–0.564	.000	–0.556	.000
45–49	–1.159	.000	–1.119	.000	–1.032	.000	–1.137	.000	–1.154	.000	–1.165	.000	–1.095	.000	–1.122	.000	–1.080	.000
50–54	–1.402	.000	–1.343	.000	–1.231	.000	–1.439	.000	–1.438	.000	–1.442	.000	–1.380	.000	–1.384	.000	–1.328	.000
Age difference between spouses																		
Wife older	0.035	.001	0.054	.000	0.051	.000	0.032	.000	0.040	.000	0.042	.000	0.021	.000	0.016	.000	0.038	.000
Husband 0–2 years older (ref.)																		
Husband 3–6 years older	–0.036	.001	–0.030	.001	–0.031	.000	–0.023	.000	–0.035	.000	–0.033	.000	–0.010	.014	–0.022	.000	–0.008	.034
Husband >6 years older	–0.099	.000	–0.136	.000	–0.109	.000	–0.092	.000	–0.104	.000	–0.099	.000	–0.078	.000	–0.096	.000	–0.067	.000
Children >4 years old in household																		
No (ref.)																		
Yes	0.267	.000	0.279	.000	0.221	.000	0.252	.000	0.263	.000	0.249	.000	0.251	.000	0.274	.000	0.261	.000
Husband household head																		
Yes (ref.)																		
No	–0.222	.000	–0.124	.000	–0.145	.000	–0.077	.000	–0.085	.000	–0.092	.000	–0.186	.000	–0.197	.000	–0.202	.000
Distance from parish of birth																		
Less than 10 km (ref.)																		
10–50 km	0.028	.001	0.012	.132	0.030	.000	0.028	.000	0.031	.000	0.033	.000	0.018	.000	0.024	.000	0.022	.000
More than 50 km	0.036	.000	–0.012	.109	–0.017	.009	0.069	.000	0.055	.000	0.034	.000	0.046	.000	0.040	.000	0.021	.000
Born abroad	–0.008	.715	–0.080	.000	–0.044	.009	0.112	.000	0.065	.020	0.004	.893	–0.005	.797	0.009	.595	–0.019	.196
Parish-Level Covariates																		
Female labor force rate																		
Low (1st quartile)	0.008	.492	0.020	.054	0.050	.000	–0.008	.143	–0.004	.529	0.005	.329	–0.006	.251	–0.007	.171	0.013	.010
Medium (2nd and 3rd quartiles) (ref.)																		
High (4th quartile)	–0.017	.074	–0.017	.095	–0.016	.163	–0.005	.441	–0.010	.184	–0.014	.040	–0.005	.306	–0.006	.305	–0.011	.030
Education rate (teacher/child ratio)																		
Low (1st quartile)	–0.009	.372	0.005	.583	0.011	.287	–0.009	.105	0.008	.189	0.000	.928	–0.012	.011	0.006	.254	0.010	.033
Medium (2nd and 3rd quartiles) (ref.)																		
High (4th quartile)	0.002	.835	–0.005	.619	–0.014	.218	–0.013	.028	–0.014	.029	–0.033	.000	–0.008	.117	–0.006	.227	–0.021	.000
Proportion employed in industry																		
Low (1st quartile)	0.022	.084	0.034	.008	–0.003	.835	0.024	.000	0.004	.506	0.018	.002	0.016	.006	0.023	.000	0.009	.134
Medium (2nd and 3rd quartiles) (ref.)																		
High (4th quartile)	0.002	.818	–0.015	.117	–0.022	.046	0.006	.327	–0.010	.133	0.002	.699	0.001	.794	–0.005	.354	–0.005	.335
Proportion of migrants born more than 100 km away and/or abroad																		
Low (1st quartile)	0.034	.009	0.022	.092	0.015	.268	0.001	.868	0.002	.740	–0.013	.035	–0.006	.275	–0.007	.255	0.004	.497
Medium (2nd and 3rd quartiles) (ref.)																		
High (4th quartile)	–0.033	.002	–0.017	.104	–0.017	.149	–0.001	.842	0.006	.421	0.001	.834	–0.003	.566	–0.006	.272	–0.020	.000
Population density per km^2^																		
Less than 50 (ref.)																		
50–100	–0.036	.007	0.001	.926	–0.014	.363	–0.016	.088	–0.036	.001	–0.051	.000	–0.030	.000	–0.004	.587	–0.023	.001
100–1,000	–0.051	.000	–0.042	.002	–0.045	.010	–0.017	.379	–0.037	.045	–0.049	.004	–0.054	.000	–0.045	.000	–0.055	.000
More than 1,000	–0.047	.020	–0.099	.000	–0.107	.001	–0.088	.126	–0.097	.118	–0.015	.775	–0.063	.001	–0.068	.000	–0.100	.000
Regional dummy variable																		
Less than 10 km from Stockholm (ref.)																		
10–50 km from Stockholm	0.085	.003	0.051	.241	0.072	.284	0.031	.691	0.022	.794	0.195	.017	0.041	.227	0.033	.334	0.064	.022
50–100 km from Stockholm	0.048	.030	0.010	.809	0.077	.232	0.024	.750	–0.013	.872	0.190	.018	0.002	.957	–0.016	.631	0.041	.122
100–150 km from Stockholm	0.094	.000	0.026	.510	0.083	.201	0.027	.728	0.019	.818	0.204	.011	0.023	.488	0.009	.787	0.067	.011
150–200 km from Stockholm	0.126	.000	0.073	.071	0.102	.115	0.113	.139	0.084	.306	0.265	.001	0.086	.009	0.060	.072	0.103	.000
Less than 10 km from Gothenburg	0.182	.000	0.152	.001	0.223	.004	0.190	.027	0.257	.006	0.397	.000	0.149	.000	0.173	.000	0.258	.000
10–50 km from Gothenburg	0.205	.000	0.137	.002	0.238	.000	0.216	.005	0.236	.004	0.434	.000	0.141	.000	0.159	.000	0.201	.000
50–100 km from Gothenburg	0.183	.000	0.139	.001	0.194	.003	0.209	.006	0.200	.015	0.424	.000	0.156	.000	0.151	.000	0.230	.000
Less than 10 km from Malmö	0.161	.000	0.146	.003	0.177	.022	0.139	.093	0.002	.981	0.294	.001	0.175	.000	0.098	.012	0.196	.000
10–50 km from Malmö	0.177	.000	0.043	.294	0.117	.075	0.126	.099	0.071	.385	0.280	.001	0.126	.000	0.057	.091	0.168	.000
50–100 km from Malmö	0.139	.000	0.076	.067	0.125	.056	0.165	.031	0.131	.110	0.328	.000	0.149	.000	0.098	.004	0.162	.000
Gotland	0.035	.343	0.011	.830	0.039	.596	–0.138	.074	–0.152	.067	0.094	.250	–0.081	.022	–0.077	.034	0.018	.546
Southern Norrland and Kopparberg county	0.132	.000	0.093	.021	0.149	.022	0.125	.101	0.111	.176	0.320	.000	0.097	.003	0.094	.005	0.157	.000
Northern Norrland	0.235	.000	0.150	.001	0.254	.000	0.365	.000	0.324	.000	0.525	.000	0.178	.000	0.136	.000	0.213	.000
Other areas (central and southern Sweden)	0.190	.000	0.124	.001	0.182	.005	0.206	.007	0.173	.034	0.384	.000	0.160	.000	0.132	.000	0.205	.000
Constant	1.101	.000	1.122	.000	1.005	.000	1.084	.000	1.114	.000	0.920	.000	1.117	.000	1.139	.000	1.033	.000
Number of Women	59,047		69,971		86,593		239,268		220,105		200,589		282,534		296,842		331,914	
Number of Parishes	2,408		2,409		2,416		2,422		2,426		2,428		2,435		2,435		2,435	
Spatial Autocorrelation Diagnostics																		
Moran’s I dependent variable	0.059	.000	0.049	.000	0.049	.000	0.371	.000	0.353	.000	0.307	.000	0.276	.000	0.223	.000	0.210	.000
Moran’s I residuals	-0.007	.584	-0.012	.353	-0.020	.108	0.109	.000	0.039	.001	0.067	.000	0.069	.000	0.061	.000	0.064	.000

*Notes:* The Moran’s I is derived at the parish level; the neighborhood is defined as the five nearest neighbors, with each neighbor given equal weight. Parishes with no observations are excluded from the calculation of the Moran’s I prior to constructing the spatial weight matrices in which information on the five nearest neighboring parishes is stored. Moran’s I tests for alternative spatial weight matrix specifications are presented in the online appendix (section 5).

*Sources:* Swedish National Archives et al. (2011a, 2011b, 2014), own calculations.

In presenting the model outcomes, we focus mainly on the three models for all SES groups. In all models, the estimates of the biodemographic controls are statistically significant and in the expected directions. The SES differences across the three censuses (Table 3) corroborate our descriptive analysis, with the net fertility levels of the elite SES group becoming increasingly distinct from the other SES groups over time.

An interesting temporal pattern emerges for our individual-level lifetime net migration variable. In 1880, women living within 10 km of their birth parish (reference category) had the lowest net fertility. This result does not fit with our expectation that women living far from their birthplace were forerunners in the fertility decline. However, this pattern shifted over time. In 1900, women who were born abroad had significantly lower fertility than women in our reference category, and the differences between women in the reference group and women living more than 50 km away decreased substantially. This pattern appears to be even more pronounced in the models for elite women (Table 4), which show that in 1900, both women born abroad and women born more than 50 km away from their place of residence had significantly lower fertility than the reference group, whereas this was not the case in 1880.

To explore our supposition that long-distance migrants—and especially those living in densely populated contexts—had greater incentives to reduce their fertility, we specified additional models in which we included an interaction between individual-level migration background and population density. The outcomes (see the online appendix, section 6) suggest that such a mechanism might help to explain the shifts in the fertility outcomes of long-distance migrants across all SES groups and among workers and others, but not among the elite. Our contextual lifetime net migration measure corresponds with the temporal pattern for the individual-level variable: we obtain a significantly lower fertility outcome for women in parishes with a high share of long-distance migrants for 1900 only (Table 3).

Among the contextual parish-level variables, we frequently observe the emergence or the strengthening of a significant negative gradient in the association with fertility levels. Such developments are visible for female labor force participation and population density, and to some extent also for education and the proportion employed in industry. The regional dummy variable accounts for unexplained fertility variation around the three biggest cities (Stockholm, Gothenburg, and Malmö)^10^ and in areas with distinct fertility patterns (the island of Gotland and two areas in northern Sweden). When we introduce the regional dummy variable, the Moran’s I values for the model residuals are substantially reduced: from 0.346 to 0.194 for the 1880 model, from 0.320 to 0.162 for the 1890 model, and from 0.250 to 0.142 for the 1900 model. Models without the regional dummy variable are available in the online appendix, section 6. A comparison shows that the drastic decreases in spatial autocorrelation in our model residuals have almost no effect on the estimates for our individual-level covariates, but they alter the outcomes for some of the parish-level covariates. However, apart from one exception in a model for farmers, who experienced only a slight fertility decline during the study period, none of the statistically significant coefficients change sign when we introduce the regional dummy variable. The regional dummy coefficients provide mixed support for the existence of nebula-like diffusion clusters of unexplained fertility decline around big cities. In the models for all SES groups—and in the 1900 model in particular—only the cluster of low fertility in the area around Stockholm cannot be completely explained.

In assessing whether our main model outcomes are biased by unaccounted spatial autocorrelation in the residuals, we see two potential problems of concern. The first is that the detected remaining spatial autocorrelation might cause bias in our estimates. The second is that especially in the models for the different SES groups, in which the numbers of women in specific locations can be quite small, we might find it difficult to isolate statistical signals for spatial autocorrelation from random noise.

To explore the first potential problem, we conducted sensitivity checks with conditional autoregressive models that allowed us to integrate the remaining spatial autocorrelation into the random effects (see the online appendix, section 6). The outcomes for the individual-level variables hardly changed when we replaced the random effects with spatial random effects. The parish-level covariates were slightly more affected, and we saw some shifts in significance levels. However, the observation of a tendency toward the emergence of negative gradients between net fertility and parish-level controls for socioeconomic conditions still held. In exploring the second potential problem, we were particularly concerned that the nonsignificant Moran’s I values for the elite models stem from the small numbers of elite women in many parishes. To address this concern, we conducted sensitivity checks in which we excluded stepwise from our sample parishes with fewer than 2–40 women before deriving the Moran’s I. The upper threshold of 40 women was determined by variance checks, which suggested that random noise is of particular concern among parishes with fewer than 30 women. The results of our sensitivity checks suggest that the insignificant Moran’s I for the residuals of the elite model in 1880 is likely an artifact of random noise. The insignificant Moran’s I tests for the 1890 and 1900 elite models, on the other hand, turned out to be quite robust. In the sensitivity tests on the 1890 model, we did not obtain a significant Moran’s I in any of our 39 tests; for the 1900 model, only 3 of the 39 tests returned significant outcomes at the .05 level. The results suggest that these two models are not greatly affected by bias due to spatial autocorrelation.

We also performed a number of additional consistency checks. These included models in which we dropped the dummy variable for children aged 5 and older because of the aforementioned endogeneity concerns. Furthermore, we specified models in which we excluded the more sparsely populated northern part of Sweden, given that this region might have differed from central and southern Sweden. Finally, we specified models in which we exchanged our dependent variable with a CWR that considers only children under age 1. This allowed us to determine whether the model outcomes would differ if they were no longer affected by potentially selective mortality above age 0. These consistency checks, which are presented in the online appendix (section 6), indicate that the main findings derived from our models are quite robust.

## Discussion and Conclusion

In this study, we have explored the relevance of geography and social status for understanding fertility variation at the onset of the Swedish fertility transition. In addition to corroborating previous findings that the elite was a vanguard group, we show that the spatial fertility decline pattern was more homogenous for the elite than for other SES groups. These outcomes suggest that among the elite, access to information relevant to the adoption of fertility-controlling strategies was less constrained by spatial distance. Meanwhile, farmers and workers and others lagged behind in both central and peripheral areas. In many locations, the net fertility of the non-elite groups was still rising when the net fertility of the elite had already begun to decline (including in Stockholm and Gothenburg, 1880–1890). The areas where non-elite groups experienced an early decrease were also much more clustered around the early centers of the decline. These findings are in line with Szreter’s (1996) notion of communication communities.

In interpreting the results, it is important to stress that although our analyses enable us to detect associations between variables, they do not allow us to identify causal effects. For example, the associations between the two variables that capture migration background and our dependent variable could also be related to the presence or absence of fertility events early in life, which may in turn have affected migration decisions at later stages of the life course. In the online appendix (section 7), we present evidence showing that most of the fertility decline occurred at ages at which the lifetime net migration patterns of cohorts were already quite stable. Although this finding could be interpreted as suggesting that the direction of influence is rather from migration to fertility, our data do not allow us to determine this conclusively.

We also found support for our expectation that long-distance migrants were among the pioneers of the process. However, these results are difficult to interpret. Our finding that fertility was initially higher among long-distance migrants than among women living close to their birth parish might be linked to healthy-migrant effects, or to migration potentially providing women with better access to assets. The observation of a temporal tendency among long-distance migrants toward having a smaller fertility advantage or even lower fertility levels might be related to the higher social connectedness and lower social embeddedness of these migrants. But we also cannot rule out the possibility that long-distance migrants had particularly ambitious goals for themselves and their offspring that made them more open to reducing their fertility. Our models allowed us to investigate the structural consideration that the large number of long-distance migrants clustered in big cities might have had greater incentives to reduce their number of offspring given that they might have had less access to property ownership and local family networks of support. The outcomes suggest that these factors were not important for the elite but might have been relevant for all SES groups taken together and for workers and others. Another possible explanation for the observed shifts concerns changes in unobserved compositional characteristics. The share of long-distance migrants was growing in this period. However, given that this increase was not very large, we consider it rather unlikely that changes in compositional characteristics drove these patterns. We hope that future research will provide more definite answers about the determinants of the observed shifts.

In line with the adjustment perspective, another potential explanation for the spatially more homogenous decline pattern of the elite is that they were more likely to be influenced not only by changes in local conditions but also by developments in distant places. For example, changes in career opportunities in Stockholm may have been relevant for the elite who were living far from the capital: these families might have been able to provide their offspring with access to the education their children would need to take advantage of these new opportunities. However, in the context of this argument, it is unclear whether the quality-quantity trade-off was really of great relevance for the elite, given that this SES group was the least constrained by financial limitations (see, e.g., Bengtsson and Dribe 2014; Dribe and Scalone 2014; Molitoris and Dribe 2016). Perhaps the personal advantages associated with having a smaller childrearing workload were more relevant to the decisions of elite women.

As part of a structural argument, one could also assert that the elite reduced their fertility first because they experienced the mortality transition earlier (Bengtsson and Dribe 2010; Burström and Bernhardt 2001). We do not have information on mortality trends by SES for the whole country, but the findings of supplementary analyses on infant and child mortality by SES for three case study areas presented in our online appendix (section 4) do not provide support for such an argument. We thus consider it rather unlikely that SES differences in infant and child mortality had a strong impact on fertility decline patterns by SES.

Do these findings imply that local structural conditions were not very important for the fertility transition, given that—at least among the elite—the decline spread rapidly even to peripheral areas? It is interesting to observe that in cities such as Stockholm and Gothenburg, the timing of the onset of the fertility decline also differed by SES (Molitoris and Dribe 2016). Nevertheless, after the fertility decline started to spread within an SES group, it seems to have been most intense in economically developed areas.

Although most of the bigger Swedish cities were early centers of the fertility decline, the second-largest Swedish city of Gothenburg and its surrounding areas stand out as having experienced rather high fertility and limited fertility reductions in our study period. This anomaly might be related to the religious movements that were widespread in this area at that time. Especially in the second half of the nineteenth century, a highly conservative interpretation of Lutheranism (so-called *Schartauanism*) became popular among both farmers and workers (Jarlert 2005). This movement may have contributed to a delay in the adoption of fertility-controlling behavior. Previous research has shown that religiosity was a crucial determinant of fertility behavior in different parts of Sweden, both before and during the transition (Junkka and Edvinsson 2016; Larsson 1984). There may, however, be alternative explanations for this anomaly.

Limitations of our study include that data restrictions allowed us to analyze births only over a rather short period of 25 years. In addition, we had to investigate net fertility outcomes in 10-year intervals. Nevertheless, the rich census data and the high geographic detail allowed us to gain insights into aspects of the fertility decline, such as the interplay between geography and SES, which have so far been understudied in research on the fertility transition.

Overall, our results suggest that the fertility decline did not occur in one wave but rather in several waves differentiated by SES. This supports the view that social stratification was important for the structuring of the fertility transition. With our finding that the behavioral shift in fertility seems to have been much less constrained by spatial distance among the elite compared with other groups, we contribute to a growing body of evidence indicating that the impact of spatial context on individual-level demographic outcomes differs substantially by SES (see, e.g., Andreev et al. 2011; Harper 2013).

## Electronic supplementary material

ESM 1 (PDF 5.59 mb)

## Footnotes

1 The role of local social embeddedness likely shifts during the fertility transition. It might impede change initially, but it could accelerate the process if an increasing share of individuals with similar characteristics in a locality changed their fertility behavior (Rosero-Bixby and Casterline 1993).

2 We use a linear instead of a Poisson specification because some of our spatial analysis methods have been developed for linear models and have not been thoroughly tested for Poisson models. Sensitivity checks with Poisson models showed that this choice does not greatly affect our results.

3 Moran’s I index is defined as: $I=\left(\frac{n}{\sum \limits_{i=1}^n\sum \limits_{j=1}^n{\mathbf{W}}_{ij}}\right)\frac{\sum \limits_{i=1}^n\sum \limits_{j=1}^n{\mathbf{W}}_{ij}\left({y}_i-\overline{y}\right)\left({y}_j-\overline{y}\right)}{\sum \limits_{i=1}^n{\left({y}_i-\overline{y}\right)}^2},$ where *n* is the number of spatial units indexed by *i* and *j*, and **W**_*ij*_ is a matrix of spatial weights.

4 In the models for the SES groups, a small number of parishes have no individual-level observations. These parishes are excluded from the GIS file before deriving information on the five nearest neighbors *j* of a parish *i* for a specific model.

5 The inclusion of this dummy variable might raise concerns that it could act as a proxy for a proclivity for higher or lower fertility. We believe this to be unlikely because voluntary childlessness was very rare at that time. Sensitivity checks in which we excluded this variable showed that our main findings were not affected (see the online appendix, section 6).

6 Because we lack information on the transport infrastructure during our study period, we use the spherical distances between the parish centroids.

7 This decision was motivated by the massive shifts in the SES of married women in Sweden during that period (see Table 1).

8 In aggregating the data to the judicial districts, one challenge was that some small urban settlements form own districts. Because the CWRs of these units might still be affected by substantial noise, we combined urban judicial districts with less than 5,000 inhabitants in 1900 with the surrounding judicial district.

9 Malmö is located close to the Danish capital of Copenhagen, where the fertility transition started around 1880 (Coale and Watkins 1986).

10 For Stockholm, we consider a larger area because an analysis of areas from which these cities were drawing in-migrants suggested that Stockholm had a larger sphere of influence than the other two cities.
