# Supplementary material for: Spatial and Social Distance at the Onset of the Fertility Transition: Sweden, 1880–1900
Source: Demography. 2019 Jan 17;56(1):169–99. doi: 10.1007/s13524-018-0737-9 (PMC6514273; doi:10.1007/s13524-018-0737-9)
Supplement: Supplementary file 1 — (PDF 5.59 mb) [file 13524_2018_737_MOESM1_ESM.pdf]

Online Appendix

This online appendix contains supplementary information and analyses for the Demography paper titled “Spatial and Social Distance at the Onset of the Fertility Transition: Sweden, 1880–1900” by Sebastian Klüsener, Martin Dribe, and Francesco Scalone.

https://doi.org/10.1007/s13524-018-0737-9

1 Map of Swedish Parishes

Fig. OA1 Map of the 2,435 time-constant parishes considered in our multilevel models

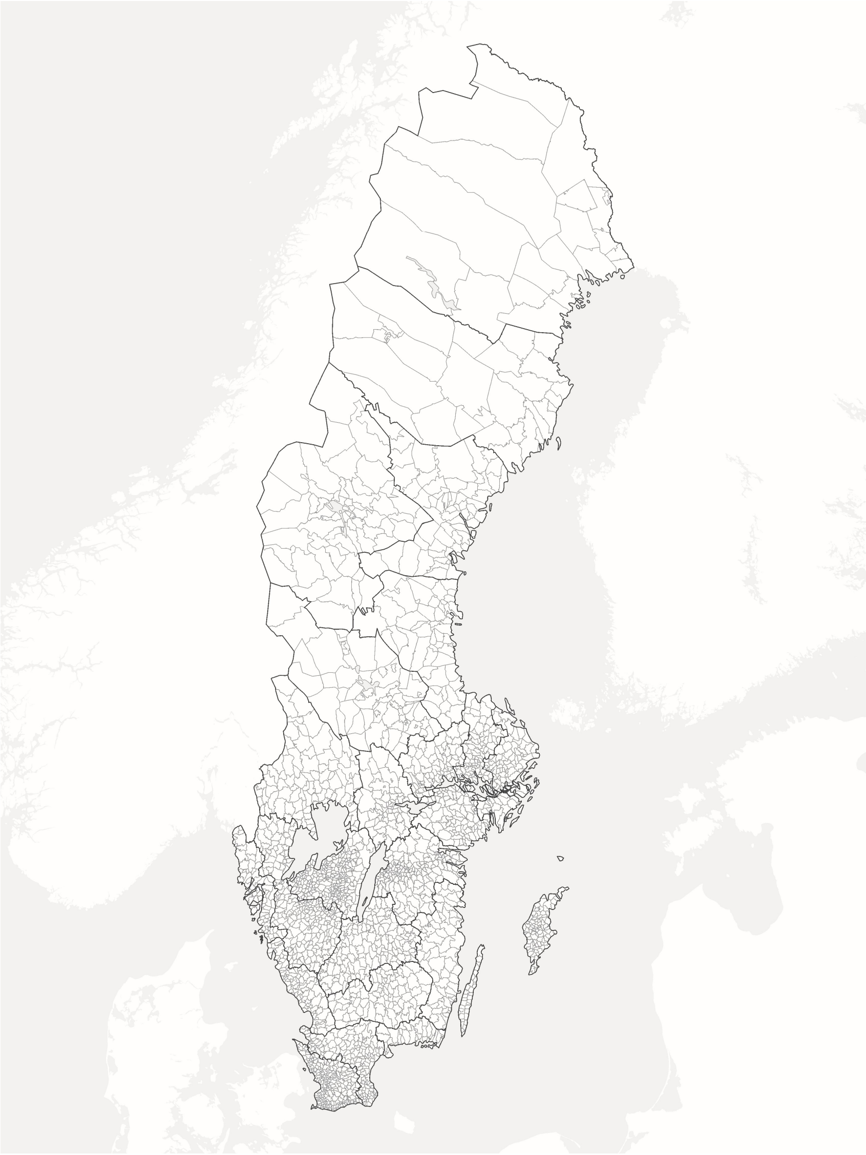

Note: Borders of the 25 Swedish counties are shown for orientation.
Base maps: Riksarkivet (2016), MPIDR Population History GIS Collection (2014)

2 Background Information on the Dependent Variable (Child-Woman Ratio)

Table OA1 Distribution – number of children aged 0–4 per married woman aged 15–54 (in %)

	1880	1890	1900
All			
0	43.4	43.6	45.7
1	30.3	29.2	28.7
2+	26.4	27.1	25.6
Total	100.0	100.0	100.0
Elite			
0	45.7	48.3	52.8
1	27.7	27.2	26.6
2+	26.6	24.6	20.5
Total	100.0	100.0	100.0
Farmers			
0	45.0	46.3	48.0
1	29.0	27.8	26.4
2+	26.0	25.9	25.6
Total	100.0	100.0	100.0
Workers and Others			
0	41.4	40.5	42.4
1	31.9	30.8	30.7
2+	26.7	28.6	26.9
Total	100.0	100.0	100.0

Sources: Swedish National Archives et al. (2011a, 2011b, 2014), own calculations.

3 Emigration by Age

Table OA2 Average emigration rates by age (per 1,000) 1880–1910

Age	Emigration Rate
0–14	2.9
15–19	13.4
20–24	19.5
25–29	12.9
30–34	7.7
35+	3.1

Source: Bohlin and Eurenius (2010, p. 536)

4 The Potential Role of Selective Mortality in the Relationship between Fertility and Net Fertility

Trends in net fertility as measured by the child-woman ratio (CWR) are driven not only by fertility trends but also by mortality developments. Our study analyzes the surviving offspring of surviving mothers at the respective census dates. We do not expect that improvements in the survivorship of mothers will affect our analysis substantially because mortality among women of these age groups was low and quite stable between 1876 and 1900 (Human Mortality Database 2017). More important are the trends in infant and child mortality, which had much higher levels and were also subject to changes over the observation period. As illustrated in Figure 1 of the main text, infant and child mortality were falling. This trend should have a positive effect on our net fertility measure, and should offset some of the observed fertility decline if we use this net fertility measure to study the fertility transition. To gain a better understanding of the magnitude of this offsetting effect, we use data from the Human Mortality Database (HMD) to derive the probabilities of the cohorts born in 1876–1880, 1886–1890, and 1896–1900 still being alive at their birthdays in 1881, 1891, and 1901. It should be noted that by using this approach, we are slightly overestimating the contribution of mortality for two reasons. First, we are deriving the estimate not for the census date (December 31 of 1880, 1890, or 1900) but for the next birthday after the census date. In addition, we use numbers for all infants, even though there are indications that infants born within marriage had somewhat higher survival chances than infants born outside marriage (see also below). In this period, about 10% of all births in Sweden were nonmarital (Statistics Sweden 1999). But we prefer to overestimate the offsetting effects due to infant and child mortality to get an upper ceiling of how this trend could distort our ability to derive from our analyses of net fertility assessments of fertility trends. The survival chances obtained from the HMD are then related to the total number of births in each cohort to derive a coarse estimate of the share of children born in the 5 years before a census who were still alive when a census was conducted (under the assumption of no selective migration from and to Sweden).

The outcomes of this analysis are presented in Table OA3 below. Our estimates of an adjusted CWR that includes children who died before the census show that there was a slight decline in this ratio as early as between 1880 and 1890. This finding suggests that in the first part of our study period the observed CWR increases might have been related to survival improvements. However, our results indicate that in the second part of this period, the offsetting effects of infant and child mortality improvements were rather small, at least at the national level (about 1.3% compared to 1890).

Table OA3 Estimate of offsetting effects of infant and child mortality

	Census 1880	Census 1890	Census 1900
Percentage alive, children aged 0–4 (coarse estimate)	82.4	85.5	86.5
Percentage alive, children aged 0–4 (1880=100) (coarse estimate)	100.0	103.6	105.0
CWR (unstandardized)	0.87	0.89	0.85
CWR (including live-born children who died before the census, coarse estimate)	1.05	1.04	0.98

Sources: Swedish National Archives et al. (2011a, 2011b, 2014), Human Mortality Database (2017), own calculations.

We will now turn to the relationship between regional net fertility levels, as assessed by our CWR and recorded fertility levels. Unfortunately, no geographically detailed information on marital fertility by socioeconomic status (SES) group is available. But for all women we can at least rely on information on regional marital fertility rates (I_g), as provided by the Princeton European Fertility project (Coale and Watkins 1986). We compare these values with our CWR levels for married women, which we obtained from our datasets. A challenging aspect of this comparison is that the I_g values provide information on fertility in single years; i.e., in 1880 or 1900, respectively (Coale and Treadway 1986). Our CWR data, on the other hand, are derived from data for longer periods (1876–1880, 1886–1890, 1896–1900). This discrepancy influences our ability to make comparisons of changes over time because these changes would have been affected not only by regional variation in infant and child mortality but also by the steepness of fertility trends in the 5 years preceding the censuses. Therefore, we also include a comparison with a CWR for children aged 0, which is restricted to children born in 1880 or 1900, respectively.

Like in the descriptive analyses presented in the paper, we have age-standardized our CWR values using our national dataset of married women aged 15–54 in 1890 as a reference. The outcomes of this analysis do not change much if we use unstandardized CWR values instead. The results of our comparison are presented in Figure OA2. For the 25 Swedish counties, the correlation between I_g and CWR levels for children aged 0–4 is 0.96 in 1880 and 0.97 in 1900. These outcomes reassure us that our analysis of regional variation in net fertility levels provides genuine insights into regional variation in fertility levels.

Fig. OA2 Comparison: Marital fertility (I_g) and child-woman ratio (CWR) of married women 1880–1900 (25 Swedish counties)

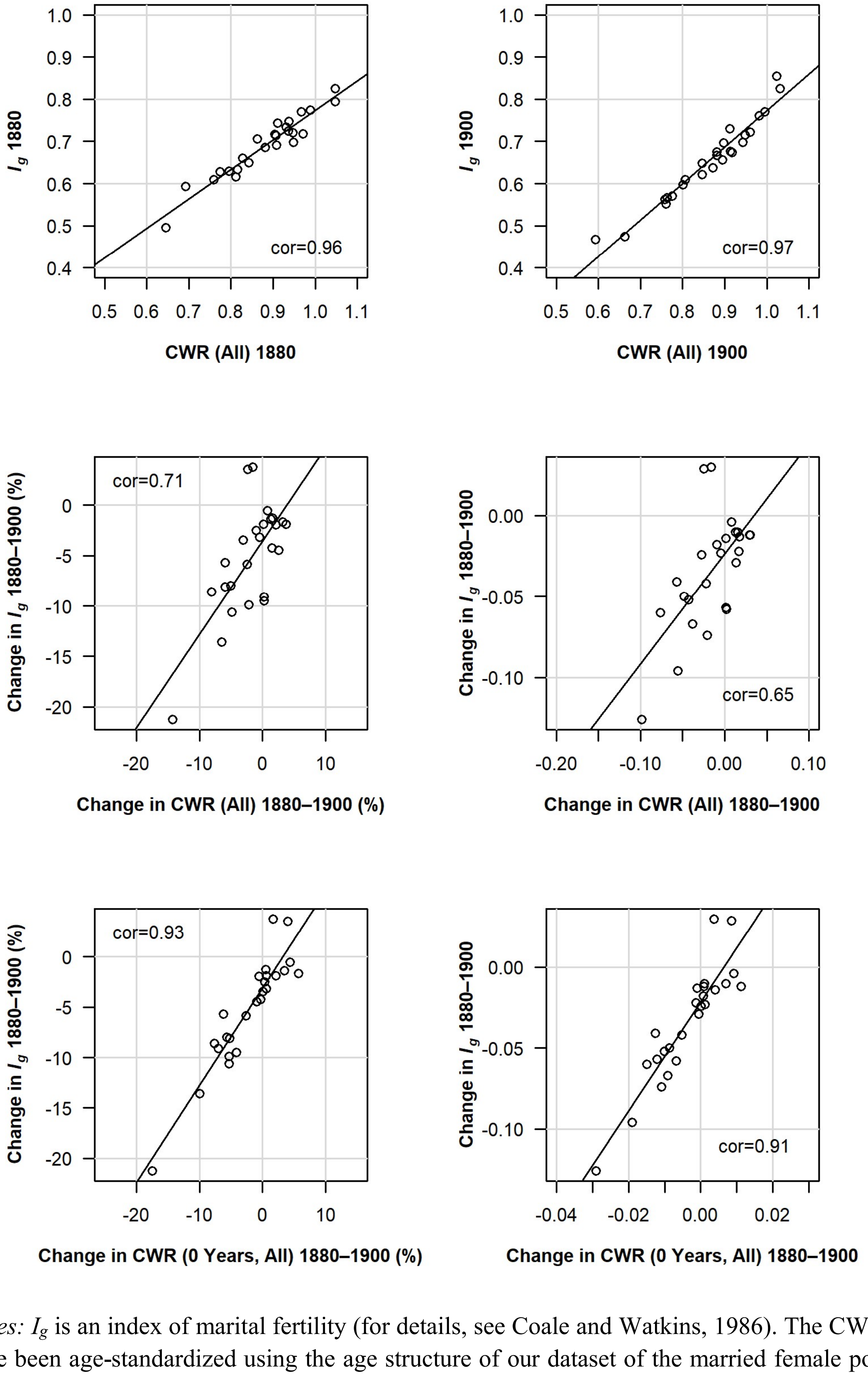

Notes: I_g is an index of marital fertility (for details, see Coale and Watkins, 1986). The CWR values have been age-standardized using the age structure of our dataset of the married female population aged 15–54 in 1890 as a reference.

Sources: Swedish National Archives et al. (2011a, 2011b, 2014), Coale and Watkins (1986)

Looking at changes over time, we first examine the correlation between the fertility and the net fertility measures that were both derived from births for the same year. These measures are plotted in the lower subplots of Figure OA2. Here, we get correlations of 0.93 for the percentage change and of 0.91 for the absolute change. If we compare our CWR of children aged 0–4 with the marital fertility rate in the two subplots in the middle of Figure OA2, the correlations become somewhat weaker. We obtain a correlation of 0.71 for the percentage change and a correlation of 0.65 for the absolute change. When we exclude the two counties in the very north of Sweden that experienced fertility increases during our study period, we derive correlations of 0.82 (percentage change) and 0.80 (absolute change). However, as stated above, the lower correlations are not only driven by regional variation in infant and child mortality. They are also affected by the circumstance that the CWR measure is calculated for surviving children born over a period of 5 years, whereas the I_g measure only refers to fertility levels in a single year. However, while the correlations between regional net fertility change and fertility change we obtained are slightly lower than those derived for the levels, they are still quite high.

To examine the potential impact of regional variation in mortality trends on our findings, we compare in Figure OA3 infant mortality levels and changes with fertility levels and changes across the 25 Swedish counties. We could obtain infant mortality levels for the counties from 1881 onward. As information on marital infant mortality was available for 1900 only, we focus here on the infant mortality rate and the total fertility rate (TFR). If we compare infant mortality and marital infant mortality in 1900 (graph not shown), we see that only one region deviates from an otherwise rather linear relationship. The city of Stockholm, which reported the highest infant mortality levels by far of all counties during our observation period, had lower marital infant mortality levels. But these levels were still high in the Swedish context since only two counties reported higher marital infant mortality levels in 1900. In the upper two subplots of Figure OA3, we show the relationship between infant mortality rate levels and TFR levels in 1880/1881 and 1900. There seems to be no statistical association for 1880/1881, while for 1900 we observe a weak correlation of 0.15. In the 1900 graph, the city of Stockholm constitutes a clear outlier, as it reported the lowest fertility levels in combination with the highest infant mortality levels.

When we compare the changes between 1880/1881 and 1900 in the two subplots in the middle of Figure OA3, we find no evidence of a statistical association if we look at change in percent, while we see an unexpected negative association if we look at absolute change. However, because the decline in infant mortality had progressed

Fig. OA3 Comparison: Infant mortality and fertility 1880–1900 (25 Swedish counties)

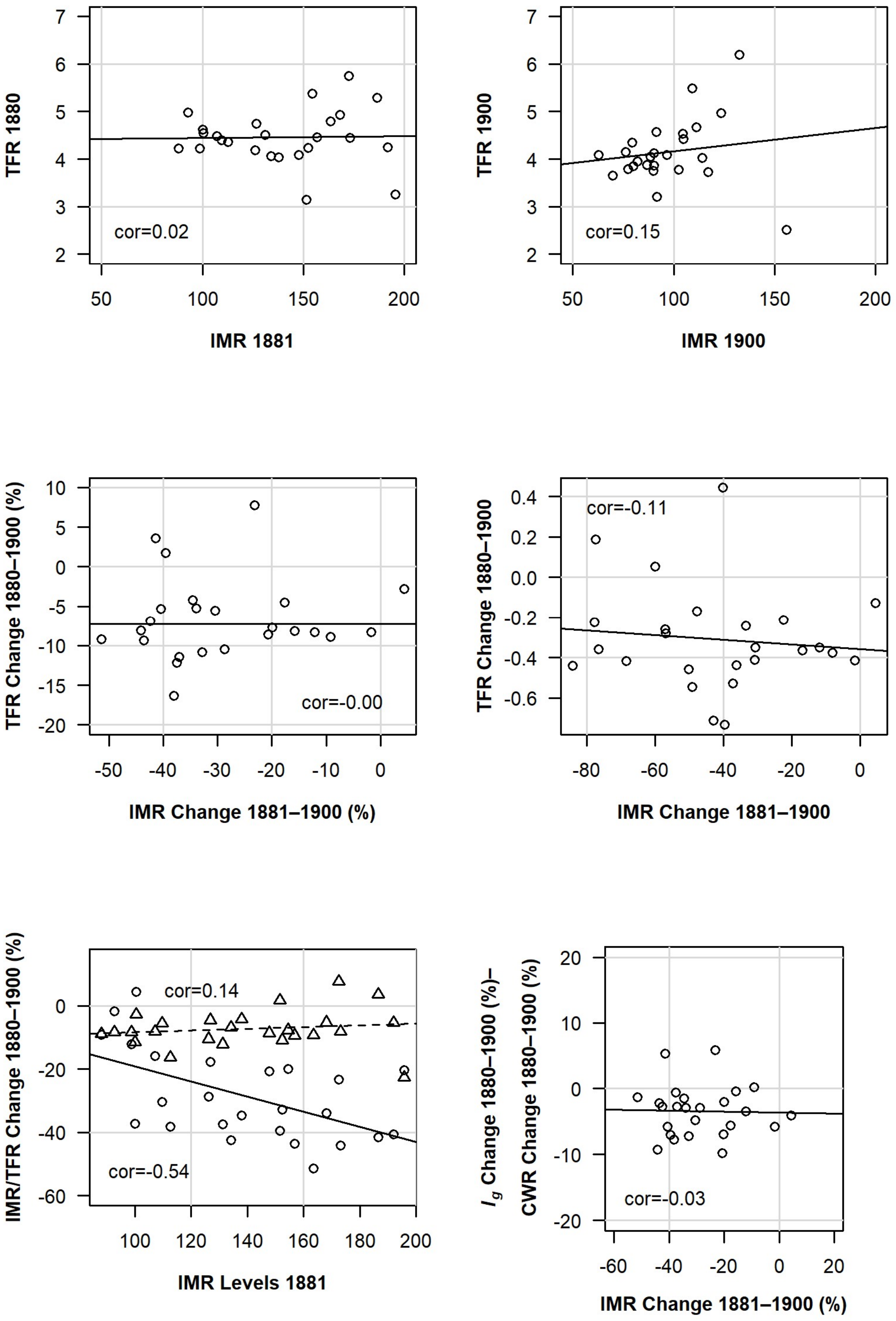

Notes: TFR refers to the total fertility rate, IMR to the infant mortality rate, and CWR to the child-woman ratio. I_g is an index of marital fertility (for details, see Coale and Watkins, 1986).

Sources: Vital statistics publications of Statistics Sweden, Statistics Sweden (1999), Coale and Watkins (1986)

substantially by the early 1880s, the already achieved infant mortality levels might have mattered more for fertility reductions once the fertility decline had started than the additional infant mortality improvements in the 1880–1900 period. This possibility is explored in the subplot on the left-hand side at the bottom of Figure OA3, where we compare infant mortality levels in 1881 with infant mortality changes in the 1881–1900 period and TFR changes in the 1880–1900 period.

For the infant mortality decline, we find a clear negative relationship with the levels in 1881, as those regions that were lagging behind in the early 1880s were able to make the most progress. For the TFR change, we obtain a weak positive association, which suggests that the fertility decline was slightly stronger in areas that had already achieved quite low infant mortality levels by 1881. The clear exception is again the city of Stockholm; it had the highest infant mortality levels in 1881, and reported the highest fertility decline in the 1881–1900 period. The subplot on the right-hand side at the bottom of Figure OA3 is perhaps the most important for our analysis. Here, we assess whether the difference we observe between the percentage change in marital fertility between 1880 and 1900 and the percentage change in our net fertility measure CWR (0–4 years) over the same period varies systematically depending on the infant mortality decline observed in that period. The plot suggests that at least at the county level this is not the case.

Our outcomes are in line with findings from multivariate analyses at the county level by Dribe (2009), in which infant mortality change was also only very weakly associated with simultaneous fertility change during the Swedish fertility transition. This, however, does not imply that these processes were not connected. We rather believe that the substantial delay between the onset of the infant mortality decline and the onset of the fertility decline during the Swedish demographic transition make it difficult to capture the relationship between these trends with models that aim to detect simultaneous associations.

In assessing whether SES variation in net fertility is related to SES variation in fertility, we can rely on outcomes of earlier studies (Dribe and Scalone 2014; Scalone and Dribe 2017). To test the influence of socioeconomic mortality differentials on the CWR values, the CWR values were corrected by taking into account SES- and age-specific mortality rates (Shryock and Siegel 1980). Because for the time under consideration age-specific mortality rates by SES are not available for Sweden as a whole, Dribe and Scalone (2014) used micro-data from study areas in Malmöhus county in southern Sweden, Stockholm city in central Sweden, and Västerbotten county in northern Sweden. These datasets are available in the Scanian Economic-Demographic Database, the Roteman Archive, and the Demographic Database of Umeå University. For

these data, Dribe and Scalone (2014) derived mortality-adjusted marital CWR values by adopting the age-specific mortality rates of the corresponding SES group.

Fig. OA4 Deriving a mortality adjusted child-woman ratio (CWR)

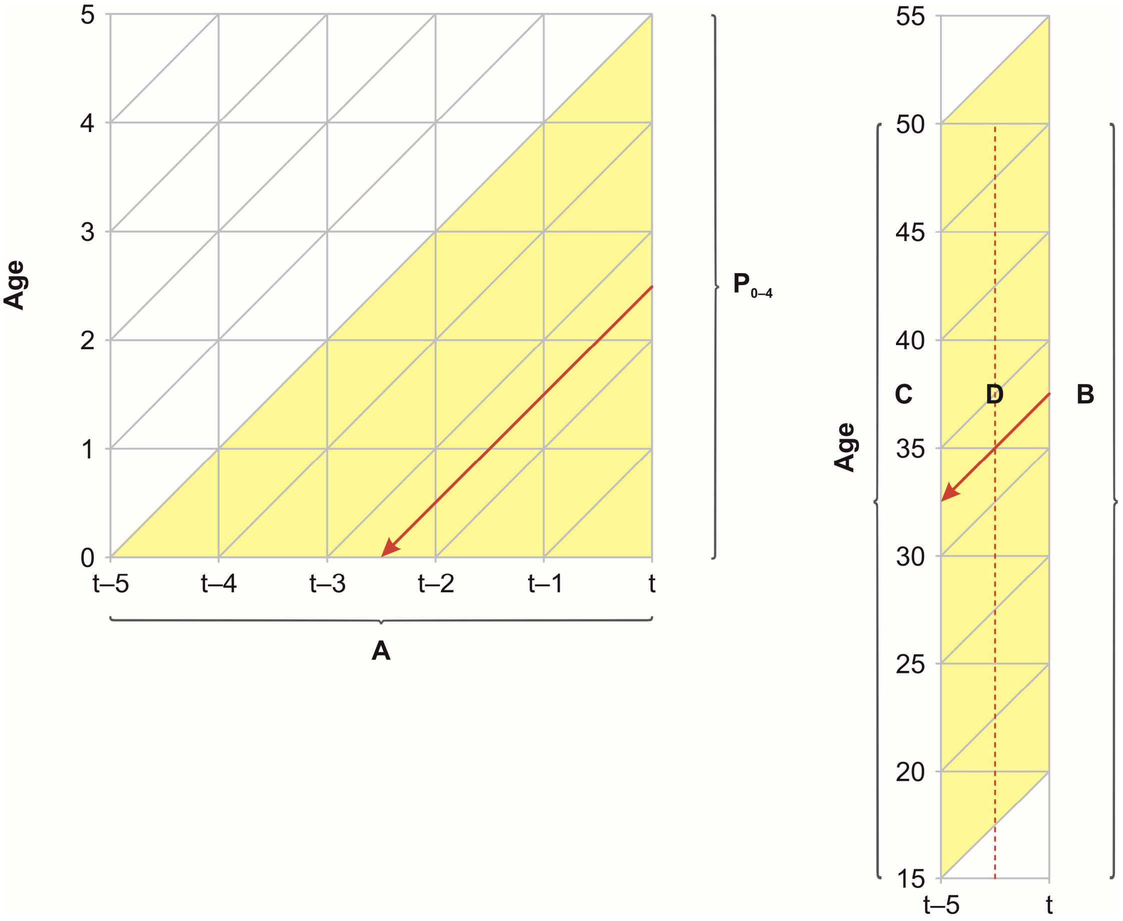

Note: t refers to year, *P* to population; A, B, C, and D are derived in the text.
Source: Dribe and Scalone (2014)

The CWR values were obtained by dividing the estimated number of births in the 5 years before the census by the mean number of women over the 5 years before the census date. As shown in Figure OA4, the segment A represents the estimated births in the previous 5 years obtained by back-projecting the number of children between age 0 and 4 at the census time.

Assuming a given life table whose radix is equal to 100,000, it will be:

(4.1) $$A = P_{0-4} \times (500{,}000/L_{0-4}).$$

The segment B represents the female population aged 15–49 at time *t*:

(4.2) $$B = P_{f15-49}.$$

Back-projecting the number of women aged 20–54 at time *t* to 5 years earlier, an estimated number of women aged 15–49 (P_{f15-49}) at time *t–5* is derived as follows:

(4.3) $$C = P_{f20-5} \times (L_{15-} \quad /L_{20-54}).$$

The average number of women P_{f15-49} between *t–5* and *t* is calculated with the following equation:

(4.4) $$D = \left[(P_{f20-5} \times (\frac{L_{15-49}}{L_{20-54}})) + P_{f15} \quad \right] \times 0.5.$$

To sum up, the adjusted CWR_{0-4} is then derived as follows:

(4.5) $$adj.\mathrm{CWR}_{0-4} = \frac{P_{0-4} \times (500{,}000/L_{0-4})}{\left[(P_{f20-5} \times (\frac{L_{15-4}}{L_{20-54}}) + P_{f15-4} \right] \times 0.5}.$$

This adjusted indicator can be used to assess the effects of socioeconomic mortality differentials on estimates of net fertility (standard CWR) (Dribe and Scalone 2014).

Figure OA5 compares the unadjusted marital CWR values with the mortality-adjusted marital CWR values. The fertility patterns by SES did not change regardless of the adopted mortality model (Dribe and Scalone 2014). Obviously, the mortality correction had some effects but not enough to change the basic fertility patterns by SES. These results support the view that in Sweden at the end of the nineteenth century, differences in the number of children born per woman explained most of the socioeconomic differences in net fertility, whereas the socioeconomic differences in mortality played only a limited role.

In order to further explore the potential effects of selective mortality, we specified as an additional consistency check models in which we used as an alternative dependent variable a CWR that just considers children aged 0. In these models, our outcomes can no longer be affected by selective variation in child mortality at ages 1–4. These models are presented in section 6 of this online appendix. Our main findings on SES differences and associations with important covariates are also observed in these models.

The evidence presented above suggests that net fertility and fertility were strongly related in our observation period. Hence, we consider it unlikely that our main conclusions would be substantially different if we were able to study fertility rates by SES instead of net fertility by SES. Since in our observation period fertility was generally characterized by stopping behavior (Dribe 2009), it is also likely that the surviving children played a larger role in the decision to adopt fertility-controlling strategies than the total number of children including those who had already died. Thus, even if there were large deviations between fertility and net fertility, we believe that an analysis of net fertility would be informative.

Fig. OA5 Marital child-woman ratio (CWR) and mortality-adjusted CWR by socioeconomic status group for Malmöhus county (southern Sweden), Stockholm city (central Sweden), and Västerbotten county (northern Sweden) in 1900

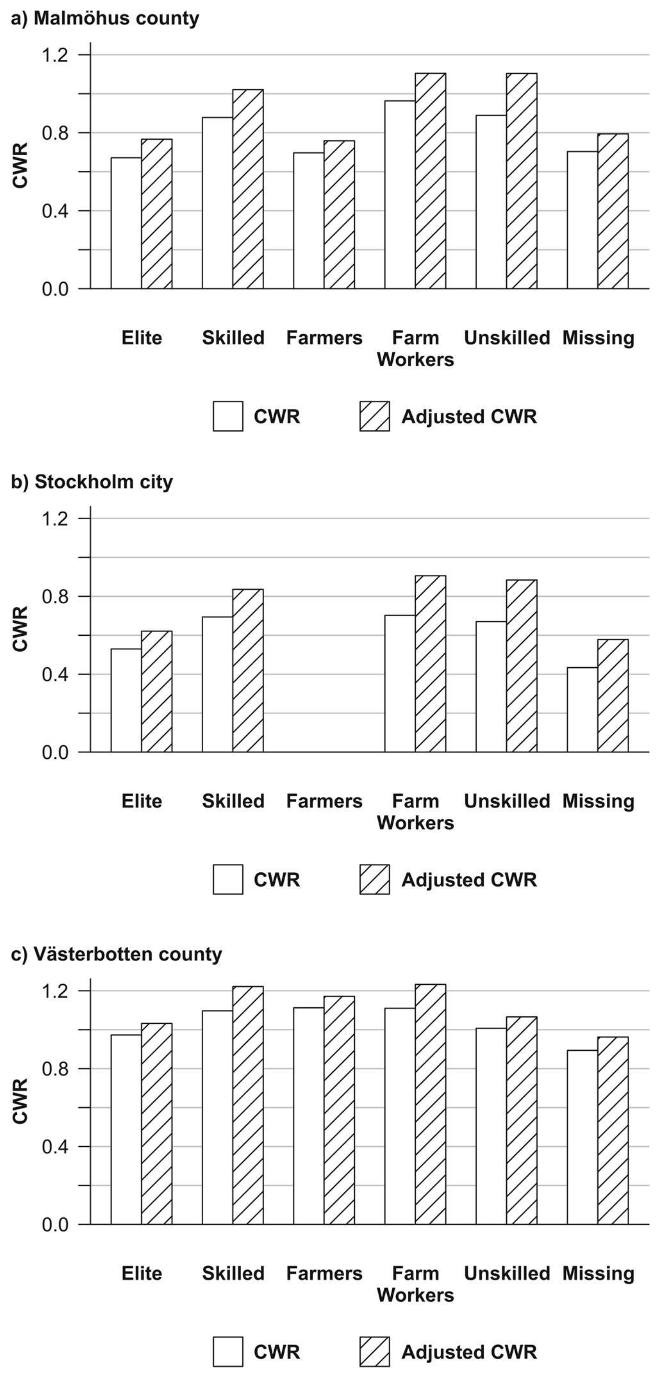

Notes: This graph is based on data from Dribe and Scalone (2014). Some SES categories slightly differ from those used in our study. Nevertheless, the SES categories are still close enough to be informative for our analysis.

Source: Dribe and Scalone (2014)

5 Spatial Autocorrelation Tests: Sensitivity Checks with Different Spatial Weight Matrices

The Moran's I tests for spatial autocorrelation in our paper are based on spatial weight matrices that treat for each parish *i* the five nearest parishes in the surrounding as neighbors *j*. The distance between parishes is derived as the spherical distance between their geographic centroids. Each neighbor gets equal weight, and the weights are row-standardized so that the sum of the weights of the neighbors adds up to 1. To explore whether the outcomes of these tests are sensitive to the choice of the weight matrix, we implemented consistency checks with other weight matrices. These checks included one contiguity-based weight matrix; it is derived using a so-called first-order queen definition of adjacency that considers those parishes as neighbors *j* of a parish *i* that share at least one common border point with that parish. In addition, we provide Moran's I values for weight matrices with three, four, five, six, and seven nearest neighbors; and calculations for distance-based weight matrices that consider as neighbors *j* all parishes with geographic centroids that are situated within a radius of 10, 20, or 30 km around the centroid of a parish *i*. In the contiguity-based and the distance-based weight matrices, some parishes have no neighbors as they are located on small islands or in areas where parishes cover quite large territories (the latter parishes are mostly clustered in northern Sweden). These parishes with no neighbors are not considered when the Moran's I is derived (see notes of Tables OA4 and OA5 for details).

Table OA4 provides the Moran's I tests for the dependent variable. It shows that the Moran's I is subject to some changes if different weight matrices are used. The Moran's I values obtained for the five nearest neighbors weight matrix tend to be in the upper spectrum. This reassures us that the choice of our preferred weight matrix does not systematically increase the likelihood that we are detecting lower levels of spatial autocorrelation than we would using other weight matrices. Qualitatively, all of the weight matrices return the same outcome: namely, that the values of the dependent variable are spatially autocorrelated, with the elite reporting the lowest Moran's I values. Also the Moran's I tests on the residuals of our preferred models presented in the paper (Table OA5) are not substantially shifting; the five nearest neighbors weight matrix tends to report rather high (positive or negative) Moran's I values. Among the elite models, which are of particular concern for our spatial autocorrelation analysis, we obtain for the 1880 and 1890 models insignificant results, independent of the weight matrix chosen. The significance levels vary slightly for the 1900 model only. For the six and the seven nearest neighbors, the Moran's I is significant at the 0.05 level. But in these cases, we are confronted with negative spatial autocorrelation. This is less problematic, as it tends to deflate the significance levels and to make the model estimates more conservative.

Table OA4: Moran's I test for spatial autocorrelation of the dependent variable (outcomes for different spatial weight matrices)

Year<br>SES	1880<br>All	1890<br>All	1900<br>All	1880<br>Elite	1890<br>Elite	1900<br>Elite	1880<br>Farmers	1890<br>Farmers	1900<br>Farmers	1880<br>Workers & Others	1890<br>Workers & Others	1900<br>Workers & Others
FOQ	0.464	0.470	0.418	0.058	0.063	0.060	0.378	0.361	0.317	0.279	0.214	0.209
p Val.	.000	.000	.000	.000	.000	.000	.000	.000	.000	.000	.000	.000
NN3	0.463	0.480	0.413	0.063	0.048	0.059	0.374	0.369	0.322	0.259	0.224	0.207
p Val.	.000	.000	.000	.000	.002	.000	.000	.000	.000	.000	.000	.000
NN4	0.468	0.476	0.418	0.063	0.044	0.059	0.378	0.358	0.314	0.273	0.223	0.209
p Val.	.000	.000	.000	.000	.001	.000	.000	.000	.000	.000	.000	.000
NN5	0.465	0.471	0.414	0.059	0.049	0.049	0.371	0.353	0.307	0.276	0.223	0.210
p Val.	.000	.000	.000	.000	.000	.000	.000	.000	.000	.000	.000	.000
NN6	0.461	0.467	0.408	0.062	0.051	0.042	0.364	0.352	0.311	0.276	0.219	0.208
p Val.	.000	.000	.000	.000	.000	.000	.000	.000	.000	.000	.000	.000
NN7	0.454	0.459	0.402	0.065	0.058	0.041	0.361	0.342	0.312	0.272	0.222	0.204
p Val.	.000	.000	.000	.000	.000	.000	.000	.000	.000	.000	.000	.000
d10km	0.324	0.333	0.296	0.065	0.027	0.037	0.235	0.244	0.228	0.226	0.174	0.170
p Val.	.000	.000	.000	.000	.097	.022	.000	.000	.000	.000	.000	.000
d20km	0.366	0.371	0.321	0.061	0.049	0.047	0.288	0.268	0.257	0.247	0.197	0.165
p Val.	.000	.000	.000	.000	.000	.000	.000	.000	.000	.000	.000	.000
d30km	0.363	0.364	0.325	0.045	0.044	0.046	0.284	0.268	0.257	0.230	0.190	0.163
p Val.	.000	.000	.000	.000	.000	.000	.000	.000	.000	.000	.000	.000

Notes: While the nearest neighbors weight matrices (NN3–NN7) comprise for all observations neighbors, the contiguity-based first-order queen matrix (FOQ) returns no direct neighbors for 25 parishes located on small islands. Moreover, the distance-based weight matrices (d10km–d30km) include parishes without neighbors in the specified distance around their geographic centroids (parishes without neighbors within 10 km: 424; within 20 km: 89, within 30 km: 41). All weights are row-standardized so that the weights of the neighbors *j* of each parish *i* sum up to 1.

Sources: Swedish National Archives et al. (2011a, 2011b, 2014), own calculations.

Table OA5: Moran's I test for spatial autocorrelation of the residuals of our preferred models (outcomes for different spatial weight matrices)

Year<br>SES	1880<br>All	1890<br>All	1900<br>All	1880<br>Elite	1890<br>Elite	1900<br>Elite	1880<br>Farmers	1890<br>Farmers	1900<br>Farmers	1880<br>Workers<br>& Others	1890<br>Workers<br>& Others	1900<br>Workers<br>& Others
FOQ	0.178	0.149	0.129	–0.011	0.006	–0.005	0.109	0.067	0.090	0.059	0.043	0.052
p Val.	.000	.000	.000	.413	.594	.705	.000	.000	.000	.000	.001	.000
NN3	0.193	0.167	0.134	–0.011	0.004	–0.024	0.122	0.060	0.074	0.052	0.057	0.071
p Val.	.000	.000	.000	.512	.767	.127	.000	.000	.000	.001	.000	.000
NN4	0.202	0.169	0.148	–0.006	–0.008	–0.017	0.116	0.050	0.075	0.067	0.061	0.069
p Val.	.000	.000	.000	.664	.573	.230	.000	.000	.000	.000	.000	.000
NN5	0.194	0.162	0.142	–0.007	–0.012	–0.020	0.109	0.039	0.067	0.069	0.061	0.064
p Val.	.000	.000	.000	.584	.353	.108	.000	.001	.000	.000	.000	.000
NN6	0.188	0.150	0.134	–0.002	–0.013	–0.024	0.096	0.038	0.069	0.075	0.050	0.056
p Val.	.000	.000	.000	.870	.269	.035	.000	.000	.000	.000	.000	.000
NN7	0.177	0.141	0.130	0.001	–0.008	–0.024	0.092	0.036	0.064	0.072	0.055	0.059
p Val.	.000	.000	.000	.930	.443	.025	.000	.000	.000	.000	.000	.000
d10km	0.147	0.109	0.113	0.016	–0.017	–0.011	0.071	0.027	0.055	0.058	0.052	0.060
p Val.	.000	.000	.000	.326	.333	.526	.000	.088	.001	.000	.001	.000
d20km	0.124	0.095	0.099	0.005	–0.009	0.005	0.067	0.028	0.057	0.068	0.042	0.035
p Val.	.000	.000	.000	.523	.360	.585	.000	.001	.000	.000	.000	.000
d30km	0.109	0.083	0.088	–0.002	–0.011	0.000	0.053	0.025	0.058	0.051	0.034	0.034
p Val.	.000	.000	.000	.759	.121	.973	.000	.000	.000	.000	.000	.000

Notes: While the nearest neighbors weight matrices (NN3–NN7) comprise for all observations neighbors, the contiguity-based first-order queen matrix (FOQ) returns no direct neighbors for 25 parishes located on small islands. Moreover, the distance-based weight matrices (d10km–d30km) include parishes without neighbors in the specified distance around their geographic centroids (parishes without neighbors within 10 km: 424; within 20 km: 89, within 30 km: 41). All weights are row-standardized so that the weights of the neighbors *j* of each parish *i* sum up to 1.

Sources: Swedish National Archives et al. (2011a, 2011b, 2014), own calculations.

6 Multilevel Models: Consistency Checks and Additional Analyses

In addition to estimating our preferred models presented in the main text, we performed a number of additional analyses and sensitivity checks that are discussed here. The first modified set of models also includes an interaction between the individual-level migration background variable and the parish-level population density variable. The interaction was introduced to explore the question of why we observe for the international and internal long-distance migrants a tendency toward a reduction in or a reversal of the fertility advantage relative to women living close to their birth parish over the three censuses. As these international and internal long-distance migrants were heavily clustered in densely populated areas, we posited that their migrant status might have provided them with greater structural incentives to reduce their number of offspring, because they might have been less able to rely in these densely populated contexts on their own property or on local (family) networks of support. If this was an important mechanism, we would expect to see a significant negative interaction coefficient for international and internal long-distance migrants living in densely populated contexts.

When we look at the models for all social groups (Table OA6), we find such significant negative interaction coefficients in the models for 1880 and 1890 but not in the 1900 model. If structural pressures on migrants living in densely populated environments during the fertility decline had affected this outcome, we would rather expect the coefficients for the interaction to increase as the fertility transition unfolds. When we contrast the models with the interaction with our main models, the biggest differences are visible in our individual-level covariate that accounts for the distance from the parish of birth. Those migrants who were born abroad are now found to have significantly higher fertility outcomes than locally-born people in the 1880 and 1890 models (at the 0.1 level). The coefficient in the 1900 model remains negative but is no longer significant. However, the tendency of international and internal long-distance migrants to lose their advantage over women living close to their birth parish is also detectable in the models with the interaction.

When we turn to the separate models by SES (Table OA7), we see over time a tendency toward positive interaction coefficients among the elite; a result that is not in line with the assumption that the decline among the elite international and internal long-distance migrants can be explained by their tendency to cluster in densely populated environments in which they might be challenged by structural changes. The negative coefficients for our individual-level migration background variable are particularly enlarged for 1900, when the interaction is introduced. For workers and others, on the other hand, we find a significant negative interaction coefficient among internal long-distance migrants in more densely populated contexts. However, the significant coeffi-

cients for the 1880 model are bigger than those for the 1900 model. The outcomes for the individual-level migration background variable in the models for workers and others got slightly changed due to the introduction of the interaction. But the trend toward a decreasing fertility advantage for international and internal long-distance migrants remains visible. We believe that the results of this analysis can be interpreted as providing weak support for the claim that among all social groups taken together and workers and others, the posited mechanism might have contributed to the tendency for international and internal long-distance migrants to lose their fertility advantage relative to individuals who were living close to their birth parish. For the elite, on the other hand, the outcomes do not provide support for the view that such a mechanism played a large role.

In Table OA8 and Table OA9 we present outcomes from sensitivity checks, in which we excluded the regional dummies from our main models. These regional dummies are used as coarse controls for spill-over effects around the three biggest urban centers of Sweden (Stockholm, Gothenburg, and Malmö), and to account for larger regions with distinct fertility patterns that remain unexplained in our models. The comparison between these models and our main models allows us to explore how the introduction of the regional dummies reduces the remaining spatial autocorrelation that is unexplained by our models, and how the coefficients of our models are affected by the exclusion of the dummies. The outcomes of these checks are discussed in the results section of our paper.

Additional consistency checks were made using models in which we excluded the dummy for own children aged 5 and older due to endogeneity concerns (see footnote 5 of our paper). Furthermore, we specified models in which we left out the sparsely populated northern part of Sweden to see how the model outcomes change if we focus on the more densely populated central and southern parts of the country. In another check, we estimated models in which we exchanged our dependent variable by a CWR that only considers children under age 1. This check was used to determine whether the model outcomes would shift if the model results were no longer affected by potentially selective mortality above age 0. Last but not least, we looked at whether the main outcomes of our models hold if the remaining unaccounted spatial autocorrelation in the models is integrated into the random effects. To do so, we estimated conditional autoregressive models (Besag et al. 1991) using integrated nested Laplace approximation (INLA) based on Bayesian inference (Bivand et al. 2015).

The dummy variable for children aged 5 and older is used as an indirect control for marital duration; i.e. that the couple had the option of having children for the entire 5-year period preceding the census. The inclusion of this dummy variable might

raise concerns that it could act as a proxy for a proclivity for higher or lower fertility. We believe this is unlikely, as voluntary childlessness was very rare at that time. In order to explore the impact of this variable on our model outcomes, we present in Tables OA10 and OA11 sensitivity checks in which this covariate has been excluded. As expected, we find that the impact is greatest for our age controls, as we obtained in this sensitivity check more negative coefficients for the younger ages, which likely include large shares of recently married women. The coefficients of the SES covariate are slightly higher than those of our reference group elite. This outcome might be related to the fact that the elite group was the fastest growing SES group in that time period, which increases the chances that there would be more recently married couples in this group. The tendency for the fertility advantage of international and long-distance migrants relative to locally-born people to be reduced or reversed over time comes out a bit more strongly in the models for all SES groups, as well as in the models for the elite and for workers and others. Generally, however, the main conclusions from the models remain the same if the dummy controlling for own children aged 5 and older is not included.

We will now turn to the sensitivity check in which we excluded the less densely populated northern part of Sweden from our models. For this check, we omitted data for the five northern-most counties that are considered part of the historic region of Norrland (Gävleborg, Västernorrland, Jämtland, Västerbotten, and Norrbotten). The results are presented in Table OA12 (all SES groups) and Table OA13 (by SES). In the model for all SES groups, there are two notable changes. The first change is that in the 1880 model, the coefficient for farmers is no longer significant compared to our reference group. However, the coefficients for farmers in the models for 1890 and 1900 remain significant. More remarkable are the changes for the individual-level distance from birth parish variable. The fertility outcomes for women born abroad are already significantly lower than the reference category in the 1890 model, and we find a more negative coefficient in the 1900 model as well. In addition, we observe for workers and others a significant negative coefficient in the 1900 model. We believe these findings are related to the fact that most of the analyzed foreign-born women residing in northern Sweden were from neighboring Finland and Norway (1900: 95% of all foreign-born women). These two countries experienced the onset of the fertility decline later than Sweden (Coale and Watkins 1986). In central and southern Sweden, on the other hand, the composition of foreign-born women was more diverse, and also comprised a substantial share of foreign-born women from countries in which the fertility transition was already underway during our study period (Coale and Watkins 1986), including Denmark, Germany, the United Kingdom, Belgium, and France. The combined contri-

bution of women of these nationalities to the total number of foreign-born women in our sample for central and southern Sweden amounts to 50%. The parish-level controls for socioeconomic conditions are also slightly affected, and in some cases move above or below a *p* value of .05. But the observation of a tendency toward the emergence of a negative gradient between net fertility and parish-level measures of socioeconomic development still holds.

The models in which we replaced our dependent variable with a CWR that only considers children aged 0 are presented in Table OA14. These models allow us to explore how the outcomes change if they can no longer be affected by potentially selective mortality above age 0. Here we present only the models for all SES groups, as the outcomes for the models by SES seem to become unstable (especially for the elite) when we only consider births from 1 instead of 5 years. The exchange of the dependent variable certainly has considerable effects on the coefficient levels, as we are now looking at births for 1 year instead of births for 5 years. A variable that is substantially affected is the control for children aged 5 and older. Its coefficients remain significant but are diminished more than we would have expected from reducing the period from which births are considered to one-fifth of the initial period. This finding is in line with our expectation that for explaining variation in births within the last year, a proxy for marital duration should be less relevant. Among the SES covariates, the coefficients for farmers and for others are no longer significantly different from those for the elite in the model for 1880. However, for 1890 and 1900 we observe very similar outcomes by SES. We interpret this finding as providing support for the view that at least selective child mortality between ages 1 and 5 does not strongly affect our outcomes for the SES groups. One deviation in the outcomes for the individual-level migration background variable is that the coefficient for women born abroad is already negative and significant in 1880. However, we still see shifts toward less positive or more negative values among both the internal and international long-distance migrants. The parish-level controls for socioeconomic conditions are also subject to slight shifts in coefficients and significance levels. But overall, we again observe that a tendency toward the emergence of a negative gradient between net fertility and parish-level measures of socioeconomic development holds.

In our final sensitivity check, we specified conditional autoregressive models. These do not treat the random effects for the parishes as independent from each other but allow random effects of neighboring parishes to be spatially dependent. With these models we can explore whether the main outcomes of our models hold if the remaining unaccounted spatial autocorrelation in the models is integrated into the random effects. These models with spatial random effects were not calculated with Stata, which we

used for our main models and all other sensitivity checks, because we are not aware of any Stata model that would allow us to estimate conditional autoregressive multilevel models with such a large number of first- and second-level observations. Instead, we chose the INLA library in R, which estimates the models with integrated nested Laplace approximation (INLA) based on Bayesian inference (Bivand et al. 2015; Martins et al. 2013). In these latent Gaussian models, the dependent variable y_{wi} is assumed to belong to a distribution family in which some parameter that is of the family ϕ_{wi} is linked with a structured additive predictor (η_{wi}) by a link function $g(\cdot)$. Hence, $g(\phi_{wi}) = \eta_{wi}$. The structured additive parameter η_{wi} accounts for the effects of a set of covariates through the following formula:

(6.1) $$\eta_{wi} = \alpha + \sum_{m=1}^{M} f^{(m)}(u_{mi}) + \sum_{l=1}^{L} \beta_l \boldsymbol{X}_{l,wi} + \sum_{k=1}^{K} \beta_k \boldsymbol{Z}_{k,i} + \varepsilon_{wi},$$

where, as in our main models, α is an intercept, $\boldsymbol{X}$ represents a vector of individual-level covariates, $\boldsymbol{Z}$ is a vector of parish-level covariates, while ε is the error term. The $f^{(j)}$ are functions on a set of n_f random effects on a vector of covariates u. For details on how the posterior marginal distributions of the model parameters are derived for this model, see Martins et al. (2013) and Bivand et al. (2015).

As we switched to another estimation procedure, we first specified random-effects models similar to the main models in our paper that are calculated with Stata using the xtreg command. In these models, we assume the random effects to be distributed as follows:

(6.2) $$\zeta_i \sim N(0, \tau_\zeta),$$

where τ_ζ refers to the precision of the Gaussian distribution. In the models for all SES groups, we observe almost identical values if we compare our coefficient estimates from the main models with the mean values of the posterior marginals of the INLA models. The deviation is a maximum of 0.001 for the individual-level covariates and a maximum of 0.002 for the parish-level covariates (the outcomes of these models are not shown but can be requested from the authors). The conclusions derived from the p values of the main models and the posterior marginal distributions of the INLA models also correspond closely. It is reassuring that using a very different estimation procedure we are able to obtain almost exactly the same outcomes. Among the models for the three different SES groups, we observe for the individual-level covariates a maximum deviation of 0.001, while the estimates for the parish-level variables vary a bit more, especially for the elite and the farmers. We believe these findings might be related to the estimation methods used, which makes a slight difference when there are many par-

ishes with a small number of individual-level observations. Such parishes are particularly prevalent in the models on the elite and on the farmer women. However, for the parish-level covariates, we obtain a deviation bigger than 0.003 in only five instances, while the outcomes for the regional dummies are bigger than 0.010 in only a few cases. Overall, however, these deviations do not affect the main conclusions derived from the models.

We then used a conditional autoregressive model in which spatial autocorrelation is specified as a Gaussian distribution with a zero mean and a precision matrix that accounts for correlation between neighbors (Bivand et al. 2015). As the latent effects are Gaussian Markov Random Fields, a variance-covariance matrix of the spatial random effects can be specified as follows:

(6.3) $$\Sigma = \frac{1}{\tau}\boldsymbol{W}^{-1},$$

where τ is a precision parameter, while $\boldsymbol{W}$ provides information on the neighbors. $\boldsymbol{W}$ is a matrix in which element $\boldsymbol{W}_{ii}$ is n_i, the number of neighbors of parish i, and element $\boldsymbol{W}_{ij}$ (with $i \neq j$) is –1 if areas i and j are neighbors, and is zero otherwise. The spatial random effects v_i are then distributed as follows:

(6.4) $$v_i | v_j, \tau_v \sim N(\frac{1}{n_i}\sum_{i\sim j} v_j, \frac{1}{\tau_v(n_i)})\ i \neq j,$$

where τ_v is a precision parameter of the random effects.

In specifying the models, we faced a number of limitations. One limitation was that these conditional autoregressive models can only be estimated with symmetric spatial weight matrices. This implies that we could not use our preferred five nearest neighbors weight matrix but instead had to use a matrix in which the neighborhood of pairs of regions is always reciprocal. We thus decided to use a contiguity-based first-order queen weight matrix in which all of the regions that share at least one border point are considered neighbors. Another limitation was that the computer servers on which we were running the models only had enough computational power to derive just the posterior marginal distributions for our model parameters for the full samples, which consisted of more than 600,000 individuals. The available capacities did not allow us to make the predictions for our individual-level observations, which were derived in these models during the estimation process. As we could not derive the predictions, we were not able to calculate Moran's I tests on the residuals. We were, however, able to implement the estimations for smaller subsamples, which allowed us to derive the predictions (results are available upon request from the authors). The Moran's I tests performed on the parish mean values were either insignificant or in the case of the

elite slightly negative and significant. The latter result is of less concern for our analysis, as negative spatial autocorrelation tends to deflate significance levels. The outcomes of the elite models generally have to be interpreted with caution, as it has been noted that such conditional autoregressive models might lead to misleading results if the existing spatial autocorrelation is already accounted for by the available controls before introducing the spatial random effects (Riebler et al. 2016; who refer to these models as Besag-models).

The outcomes for all women are presented in Table OA15, while the models for the different SES are shown in Table OA16. The inclusion of the remaining spatial autocorrelation in the random effects has almost no influence on the outcomes for our individual-level control variables. This comprises the outcomes for the SES covariate and the individual-level migration background covariate. As in the other sensitivity checks, we see some changes in the parish-level controls for socioeconomic conditions. The 0.025 and 0.0975 quantiles of the posterior marginal distributions are subject to slight shifts, such that in some cases the distributions are compared to the models with random effects entirely situated above or below zero, while in other cases the opposite can be detected. But again, the tendency toward the emergence of negative gradients in the association between net fertility and our socioeconomic parish-level covariates remains visible. The biggest changes are observed among the regional dummies, which we introduced to control for spatial spill-over effects and to account for fertility variation across bigger regions that we are not able to account for in our models. As these regional dummies have a clear spatial dimension, they are likely to be strongly affected if random effects are replaced by spatial random effects. But the shifts in the outcomes for the regional dummies do not change the general conclusions we derive from them. The results of this sensitivity check with conditional autoregressive models provide us with additional reassurance that the main findings of our models are not just artifacts of bias due to spatial autocorrelation.

Table OA6 Model estimates for the number of children aged 0–4 per married woman aged 15–54 (with interaction effects between migration background and population density)

	1880		1890		1900	
	Coef.	*p* Val.	Coef.	*p* Val.	Coef.	*p* Val.
Individual-Level Covariates						
Age of woman						
15–19 years	–0.605	.000	–0.589	.000	–0.477	.000
20–24 years	–0.214	.000	–0.150	.000	–0.101	.000
25–29 years	0.054	.000	0.079	.000	0.120	.000
30–34 years (ref.)						
35–39 years	–0.207	.000	–0.218	.000	–0.221	.000
40–44 years	–0.571	.000	–0.584	.000	–0.576	.000
45–49 years	–1.116	.000	–1.131	.000	–1.100	.000
50–54 years	–1.404	.000	–1.397	.000	–1.353	.000
Age difference between spouses						
Wife older	0.026	.000	0.028	.000	0.040	.000
Husband 0–2 years older (ref.)						
Husband 3–6 years older	–0.017	.000	–0.027	.000	–0.019	.000
Husband >6 years older	–0.083	.000	–0.102	.000	–0.083	.000
Children >4 years old in household						
No (ref.)						
Yes	0.252	.000	0.270	.000	0.252	.000
Husband household head						
Yes (ref.)						
No	–0.153	.000	–0.145	.000	–0.157	.000
Socioeconomic status						
Elite (ref.)						
Farmers	0.012	.001	0.049	.000	0.088	.000
Skilled workers	0.050	.000	0.085	.000	0.096	.000
Lower-skilled workers	0.060	.000	0.096	.000	0.114	.000
Unskilled workers	0.006	.128	0.059	.000	0.091	.000
Others	–0.021	.000	–0.002	.701	0.035	.000
Distance from parish of birth						
Less than 10 km (ref.)						
10–50 km	0.025	.000	0.029	.000	0.031	.000
More than 50 km	0.065	.000	0.038	.000	0.016	.000
Born abroad	0.050	.007	0.028	.092	–0.020	.210
Parish-Level Covariates						
Female labor force rate						
Low (1st quartile)	–0.006	.166	–0.004	.321	0.010	.017
Medium (2nd/3rd quartiles) (ref.)						
High (4th quartile)	–0.006	.188	–0.009	.056	–0.014	.002
Education rate (teacher/child ratio)						
Low (1st quartile)	–0.010	.010	0.008	.061	0.005	.203
Medium (2nd/3rd quartiles) (ref.)						
High (4th quartile)	–0.011	.010	–0.011	.013	–0.027	.000
Proportion employed in industry						
Low (1st quartile)	0.021	.000	0.017	.000	0.016	.001
Medium (2nd/3rd quartiles) (ref.)						
High (4th quartile)	0.001	.763	–0.009	.041	–0.005	.238

Proportion of migrants born more than 100 km away and/or abroad						
Low (1st quartile)	0.001	.757	0.001	.816	–0.003	.509
Medium (2nd/3rd quartiles) (ref.)						
High (4th quartile)	–0.005	.369	–0.001	.872	–0.009	.048
Population density per km²						
Less than 50 (ref.)						
50–100	–0.022	.002	–0.021	.005	–0.034	.000
100–1,000	–0.044	.000	–0.031	.001	–0.046	.000
More than 1,000	–0.042	.057	–0.083	.000	–0.101	.000
Distance from parish of birth * population density per km²						
Less than 10 km/less than 50 (ref.)						
10–50 km/50–100	–0.001	.882	–0.001	.915	–0.003	.767
10–50 km/100–1,000	0.000	.969	–0.020	.020	–0.018	.024
10–50 km/more than 1,000	–0.031	.005	0.004	.665	–0.009	.323
More than 50 km/50–100	–0.047	.000	0.006	.620	0.002	.870
More than 50 km/ 100–1,000	–0.063	.000	–0.045	.000	–0.021	.015
More than 50 km/more than 1,000	–0.045	.000	0.008	.351	0.003	.717
Born abroad/50–100	–0.061	.179	–0.036	.457	0.021	.607
Born abroad/100–1,000	–0.003	.933	–0.037	.228	–0.025	.369
Born abroad/more than 1,000	–0.126	.000	–0.129	.000	–0.024	.316
Regional dummies						
Less than 10 km from Stockholm (ref.)						
10–50 km from Stockholm	0.029	.440	0.026	.461	0.073	.031
50–100 km from Stockholm	–0.007	.854	–0.023	.500	0.054	.099
100–150 km from Stockholm	0.011	.767	0.001	.986	0.071	.030
150–200 km from Stockholm	0.080	.029	0.056	.103	0.112	.001
Less than 10 km from Gothenburg	0.159	.000	0.174	.000	0.254	.000
10–50 km from Gothenburg	0.161	.000	0.178	.000	0.248	.000
50–100 km from Gothenburg	0.162	.000	0.156	.000	0.250	.000
Less than 10 km from Malmö	0.150	.000	0.078	.048	0.196	.000
10–50 km from Malmö	0.107	.004	0.047	.171	0.158	.000
50–100 km from Malmö	0.131	.000	0.096	.006	0.173	.000
Gotland	–0.117	.002	–0.126	.000	–0.014	.689
Southern Norrland & Kopparberg county	0.091	.013	0.084	.014	0.168	.000
Northern Norrland	0.254	.000	0.219	.000	0.299	.000
Other areas (central & southern Sweden)	0.162	.000	0.135	.000	0.219	.000
Constant	1.102	.000	1.082	.000	0.950	.000
Number of Women	580,849		586,198		619,096	
Number of Parishes	2,435		2,435		2,435	
Spatial Autocorrelation Diagnostics						
Moran's I dependent variable	0.465	.000	0.471	.000	0.414	.000
Moran's I residuals	0.194	.000	0.162	.000	0.142	.000

Note: The Moran's I is derived at the parish level; neighborhood is defined as the five nearest neighbors, with each neighbor given equal weight.

Sources: Swedish National Archives et al. (2011a, 2011b, 2014), own calculations.

Table OA7 Models by socioeconomic status: Estimates for the number of children aged 0–4 per married woman aged 15–54 (with interaction effects between migration background and population density)

	Elite						Farmers						Workers and Others					
	1880		1890		1900		1880		1890		1900		1880		1890		1900	
	Coef.	*p* Val.	Coef.	*p* Val.	Coef.	*p* Val.	Coef.	*p* Val.	Coef.	*p* Val.	Coef.	*p* Val.	Coef.	*p* Val.	Coef.	*p* Val.	Coef.	*p* Val.
Individual-Level Covariates																		
Age of woman																		
15–19 years	–0.616	.000	–0.674	.000	–0.390	.000	–0.740	.000	–0.713	.000	–0.646	.000	–0.542	.000	–0.541	.000	–0.444	.000
20–24 years	–0.234	.000	–0.137	.000	–0.126	.000	–0.216	.000	–0.163	.000	–0.142	.000	–0.205	.000	–0.143	.000	–0.076	.000
25–29 years	0.075	.000	0.101	.000	0.122	.000	0.071	.000	0.094	.000	0.123	.000	0.043	.000	0.072	.000	0.125	.000
30–34 years (ref.)																		
35–39 years	–0.250	.000	–0.262	.000	–0.240	.000	–0.217	.000	–0.223	.000	–0.226	.000	–0.191	.000	–0.207	.000	–0.217	.000
40–44 years	–0.651	.000	–0.640	.000	–0.609	.000	–0.578	.000	–0.599	.000	–0.602	.000	–0.552	.000	–0.564	.000	–0.555	.000
45–49 years	–1.159	.000	–1.119	.000	–1.034	.000	–1.137	.000	–1.154	.000	–1.165	.000	–1.095	.000	–1.121	.000	–1.080	.000
50–54 years	–1.402	.000	–1.344	.000	–1.232	.000	–1.439	.000	–1.438	.000	–1.442	.000	–1.379	.000	–1.384	.000	–1.327	.000
Age difference between spouses																		
Wife older	0.036	.001	0.053	.000	0.051	.000	0.032	.000	0.040	.000	0.042	.000	0.021	.000	0.016	.000	0.038	.000
Husband 0–2 years older (ref.)																		
Husband 3–6 years older	–0.036	.001	–0.030	.001	–0.031	.000	–0.023	.000	–0.036	.000	–0.033	.000	–0.010	.014	–0.022	.000	–0.008	.034
Husband >6 years older	–0.099	.000	–0.135	.000	–0.109	.000	–0.092	.000	–0.104	.000	–0.099	.000	–0.078	.000	–0.096	.000	–0.067	.000
Children >4 years old in household																		
No (ref.)																		
Yes	0.267	.000	0.279	.000	0.221	.000	0.252	.000	0.263	.000	0.249	.000	0.251	.000	0.274	.000	0.261	.000
Husband household head																		
Yes (ref.)																		
No	–0.221	.000	–0.125	.000	–0.147	.000	–0.077	.000	–0.085	.000	–0.092	.000	–0.185	.000	–0.196	.000	–0.201	.000
Distance from parish of birth																		
Less than 10 km (ref.)																		
10–50 km	0.032	.003	–0.001	.914	0.010	.306	0.029	.000	0.030	.000	0.037	.000	0.020	.000	0.030	.000	0.029	.000
More than 50 km	0.063	.000	–0.033	.002	–0.041	.000	0.069	.000	0.057	.000	0.034	.000	0.065	.000	0.047	.000	0.028	.000
Born abroad	0.004	.929	–0.075	.063	–0.107	.003	0.131	.000	0.079	.010	0.004	.903	0.019	.465	0.036	.119	0.001	.948
Parish-Level Covariates																		
Female labor force rate																		
Low (1st quartile)	0.006	.560	0.019	.060	0.050	.000	–0.008	.132	–0.004	.509	0.005	.341	–0.006	.236	–0.008	.154	0.013	.010
Medium (2nd/3rd quartiles) (ref.)																		
High (4th quartile)	–0.015	.111	–0.016	.103	–0.016	.165	–0.005	.450	–0.009	.192	–0.013	.053	–0.005	.318	–0.005	.332	–0.011	.031
Education rate (teacher/child ratio)																		
Low (1st quartile)	–0.009	.377	0.005	.634	0.011	.287	–0.009	.100	0.008	.183	0.000	.928	–0.012	.010	0.006	.256	0.010	.035
Medium (2nd/3rd quartiles) (ref.)																		
High (4th quartile)	0.001	.928	–0.005	.619	–0.014	.208	–0.013	.023	–0.014	.024	–0.033	.000	–0.008	.109	–0.006	.231	–0.021	.000
Proportion employed in industry																		
Low (1st quartile)	0.025	.055	0.032	.012	–0.004	.733	0.024	.000	0.004	.495	0.018	.002	0.016	.006	0.023	.000	0.010	.114
Medium (2nd/3rd quartiles) (ref.)																		
High (4th quartile)	0.003	.802	–0.016	.105	–0.022	.044	0.006	.314	–0.010	.129	0.003	.676	0.001	.783	–0.005	.369	–0.005	.351

Proportion of migrants born more than 100 km away and/or abroad																		
Low (1st quartile)	0.036	.007	0.021	.108	0.014	.312	0.001	.872	0.003	.693	–0.013	.035	–0.006	.345	–0.007	.287	0.005	.447
Medium (2nd/3rd quartiles) (ref.)																		
High (4th quartile)	–0.033	.002	–0.015	.161	–0.014	.214	–0.002	.807	0.006	.448	0.002	.752	–0.005	.388	–0.007	.236	–0.020	.000
Population density per km²																		
Less than 50 (ref.)																		
50–100	0.004	.810	–0.016	.394	–0.027	.171	–0.014	.157	–0.037	.001	–0.043	.000	–0.028	.002	0.003	.782	–0.021	.017
100–1,000	–0.035	.052	–0.043	.016	–0.062	.003	–0.016	.462	–0.025	.274	–0.007	.745	–0.041	.000	–0.027	.011	–0.042	.000
More than 1,000	–0.034	.162	–0.136	.000	–0.151	.000	–0.005	.961	0.010	.920	0.032	.755	–0.031	.127	–0.058	.003	–0.082	.000
Distance from parish of birth * population density per km²																		
Less than 10 km/less than 50 (ref.)																		
10–50 km/50–100	–0.036	.187	0.028	.298	0.018	.477	–0.009	.477	0.008	.566	–0.025	.125	0.008	.494	–0.017	.165	–0.003	.807
10–50 km/100–1,000	0.014	.578	0.009	.673	0.036	.065	0.003	.929	–0.004	.883	–0.096	.001	–0.005	.620	–0.025	.015	–0.019	.041
10–50 km/more than 1,000	–0.018	.521	0.041	.082	0.054	.007	–0.111	.300	–0.145	.185	–0.127	.285	–0.040	.002	–0.016	.156	–0.030	.005
More than 50 km/50–100	–0.112	.000	0.041	.169	0.012	.638	0.022	.533	0.008	.828	–0.011	.739	–0.028	.098	–0.007	.667	–0.003	.800
More than 50 km/ 100–1,000	–0.064	.006	–0.006	.774	0.017	.351	–0.030	.604	–0.079	.097	–0.042	.319	–0.056	.000	–0.040	.001	–0.022	.038
More than 50 km/more than 1,000	–0.024	.257	0.072	.000	0.070	.000	–0.115	.308	–0.124	.284	0.058	.654	–0.059	.000	–0.010	.388	–0.020	.051
Born abroad/50–100	–0.040	.678	–0.045	.658	0.224	.003	–0.128	.098	–0.033	.723	0.002	.983	–0.028	.692	–0.020	.775	–0.074	.182
Born abroad/100–1,000	0.062	.383	0.033	.572	0.066	.206	0.145	.383	–0.211	.144	–0.040	.779	0.011	.829	–0.013	.749	–0.038	.309
Born abroad/more than 1,000	–0.045	.434	0.000	.995	0.090	.037	–0.770	.148	–0.612	.257	–0.026	.974	–0.139	.004	–0.123	.003	–0.038	.294
Regional dummies																		
Less than 10 km from Stockholm (ref.)																		
10–50 km from Stockholm	0.087	.003	0.058	.190	0.072	.280	0.024	.766	0.023	.782	0.218	.008	0.036	.293	0.035	.313	0.065	.021
50–100 km from Stockholm	0.053	.020	0.014	.725	0.077	.232	0.017	.827	–0.011	.889	0.213	.009	–0.001	.965	–0.014	.681	0.042	.116
100–150 km from Stockholm	0.099	.000	0.032	.436	0.083	.200	0.019	.804	0.021	.802	0.228	.005	0.020	.554	0.011	.739	0.068	.011
150–200 km from Stockholm	0.133	.000	0.080	.052	0.103	.113	0.106	.177	0.086	.298	0.289	.000	0.082	.013	0.062	.065	0.105	.000
Less than 10 km from Gothenburg	0.184	.000	0.157	.001	0.223	.004	0.180	.039	0.256	.006	0.406	.000	0.147	.000	0.176	.000	0.259	.000
10–50 km from Gothenburg	0.208	.000	0.143	.002	0.235	.000	0.209	.008	0.238	.004	0.458	.000	0.137	.000	0.160	.000	0.202	.000
50–100 km from Gothenburg	0.188	.000	0.144	.001	0.192	.003	0.201	.010	0.201	.015	0.448	.000	0.153	.000	0.154	.000	0.232	.000
Less than 10 km from Malmö	0.164	.000	0.151	.002	0.174	.024	0.131	.120	0.001	.993	0.320	.000	0.170	.000	0.099	.012	0.198	.000
10–50 km from Malmö	0.173	.000	0.049	.246	0.115	.080	0.119	.130	0.072	.382	0.302	.000	0.121	.000	0.058	.087	0.169	.000
50–100 km from Malmö	0.140	.000	0.081	.055	0.123	.059	0.157	.045	0.132	.108	0.351	.000	0.144	.000	0.100	.004	0.163	.000
Gotland	0.041	.277	0.016	.763	0.038	.604	–0.146	.067	–0.150	.072	0.118	.156	–0.084	.019	–0.074	.044	0.020	.511
Southern Norrland & Kopparberg county	0.137	.000	0.100	.015	0.148	.022	0.118	.132	0.113	.170	0.344	.000	0.094	.005	0.097	.004	0.159	.000
Northern Norrland	0.237	.000	0.156	.001	0.253	.000	0.358	.000	0.326	.000	0.550	.000	0.174	.000	0.139	.000	0.215	.000
Other areas (central & southern Sweden)	0.196	.000	0.130	.001	0.181	.005	0.199	.011	0.175	.033	0.408	.000	0.157	.000	0.134	.000	0.207	.000
Constant	1.087	.000	1.126	.000	1.021	.000	1.091	.000	1.113	.000	0.894	.000	1.118	.000	1.133	.000	1.028	.000
Number of Women	59,047		69,971		86,593		239,268		220,105		200,589		282,534		296,842		331,914	
Number of Parishes	2,408		2,409		2,416		2,422		2,426		2,428		2,435		2,435		2,435	

Spatial Autocorrelation Diagnostics																		
Moran’s I dependent variable	0.059	.000	0.049	.000	0.049	.000	0.371	.000	0.353	.000	0.307	.000	0.276	.000	0.223	.000	0.210	.000
Moran’s I residuals	–0.010	.617	–0.010	.373	–0.020	.120	0.111	.000	0.043	.000	0.065	.000	0.069	.000	0.061	.000	0.064	.000

Notes: The Moran’s I is derived at the parish level; neighborhood is defined as the five nearest neighbors, with each neighbor given equal weight. Parishes with no observations are excluded from the calculation of the Moran’s I prior to constructing the spatial weight matrices in which information on the five nearest neighboring parishes is stored.

Sources: Swedish National Archives et al. (2011a, 2011b, 2014), own calculations.

Table OA8 Model estimates for the number of children aged 0–4 per married woman aged 15–54 (without regional dummies)

	1880		1890		1900	
	Coef.	*p* Val.	Coef.	*p* Val.	Coef.	*p* Val.
Individual-Level Covariates						
Age of woman						
15–19 years	–0.606	.000	–0.590	.000	–0.478	.000
20–24 years	–0.214	.000	–0.151	.000	–0.101	.000
25–29 years	0.054	.000	0.079	.000	0.120	.000
30–34 years (ref.)						
35–39 years	–0.207	.000	–0.218	.000	–0.221	.000
40–44 years	–0.571	.000	–0.585	.000	–0.575	.000
45–49 years	–1.117	.000	–1.131	.000	–1.100	.000
50–54 years	–1.404	.000	–1.397	.000	–1.353	.000
Age difference between spouses						
Wife older	0.026	.000	0.028	.000	0.041	.000
Husband 0–2 years older (ref.)						
Husband 3–6 years older	–0.017	.000	–0.027	.000	–0.019	.000
Husband >6 years older	–0.082	.000	–0.102	.000	–0.083	.000
Children >4 years old in household						
No (ref.)						
Yes	0.252	.000	0.270	.000	0.252	.000
Husband household head						
Yes (ref.)						
No	–0.156	.000	–0.147	.000	–0.160	.000
Socioeconomic status						
Elite (ref.)						
Farmers	0.010	.010	0.050	.000	0.087	.000
Skilled workers	0.050	.000	0.086	.000	0.096	.000
Lower-skilled workers	0.060	.000	0.097	.000	0.114	.000
Unskilled workers	0.004	.292	0.059	.000	0.089	.000
Others	–0.022	.000	–0.001	.798	0.034	.000
Distance from parish of birth						
Less than 10 km (ref.)						
10–50 km	0.023	.000	0.026	.000	0.027	.000
More than 50 km	0.047	.000	0.033	.000	0.013	.000
Born abroad	0.017	.181	–0.015	.197	–0.027	.008
Parish-level covariates						
Female labor force rate						
Low (1st quartile)	0.009	.071	0.013	.012	0.031	.000
Medium (2nd/3rd quartiles) (ref.)						
High (4th quartile)	–0.031	.000	–0.039	.000	–0.048	.000
Education rate (teacher/child ratio)						
Low (1st quartile)	–0.029	.000	0.002	.756	0.007	.150
Medium (2nd/3rd quartiles) (ref.)						
High (4th quartile)	–0.022	.000	–0.029	.000	–0.038	.000
Proportion employed in industry						
Low (1st quartile)	0.048	.000	0.051	.000	0.044	.000
Medium (2nd/3rd quartiles) (ref.)						
High (4th quartile)	–0.005	.345	–0.009	.083	0.001	.802

Proportion of migrants born more than 100 km away and/or abroad						
Low (1st quartile)	0.019	.000	0.016	.003	0.022	.000
Medium (2nd/3rd quartiles) (ref.)						
High (4th quartile)	–0.056	.000	–0.040	.000	–0.047	.000
Population density per km²						
Less than 50 (ref.)						
50–100	–0.012	.107	–0.022	.002	–0.018	.020
100–1,000	–0.002	.834	0.000	.997	–0.008	.433
More than 1,000	–0.027	.316	–0.038	.122	–0.049	.029
Constant	1.228	.000	1.178	.000	1.120	.000
Number of Women	580,849		586,198		619,096	
Number of Parishes	2,435		2,435		2,435	
Spatial Autocorrelation Diagnostics						
Moran's I dependent variable	0.465	.000	0.471	.000	0.414	.000
Moran's I residuals	0.346	.000	0.320	.000	0.250	.000

Note: The Moran's I is derived at the parish level; neighborhood is defined as the five nearest neighbors, with each neighbor given equal weight.

Sources: Swedish National Archives et al. (2011a, 2011b, 2014), own calculations.

Table OA9 Models by socioeconomic status: Estimates for the number of children aged 0–4 per married woman aged 15–54 (without regional dummies)

	Elite						Farmers						Workers and Others					
	1880		1890		1900		1880		1890		1900		1880		1890		1900	
	Coef.	*p* Val.	Coef.	*p* Val.	Coef.	*p* Val.	Coef.	*p* Val.	Coef.	*p* Val.	Coef.	*p* Val.	Coef.	*p* Val.	Coef.	*p* Val.	Coef.	*p* Val.
Individual-Level Covariates																		
Age of woman																		
15–19 years	–0.617	.000	–0.677	.000	–0.389	.000	–0.737	.000	–0.711	.000	–0.646	.000	–0.543	.000	–0.542	.000	–0.446	.000
20–24 years	–0.231	.000	–0.138	.000	–0.125	.000	–0.215	.000	–0.162	.000	–0.143	.000	–0.206	.000	–0.144	.000	–0.075	.000
25–29 years	0.078	.000	0.101	.000	0.123	.000	0.071	.000	0.094	.000	0.123	.000	0.043	.000	0.071	.000	0.126	.000
30–34 years (ref.)																		
35–39 years	–0.251	.000	–0.262	.000	–0.240	.000	–0.217	.000	–0.223	.000	–0.225	.000	–0.191	.000	–0.207	.000	–0.217	.000
40–44 years	–0.652	.000	–0.641	.000	–0.608	.000	–0.578	.000	–0.599	.000	–0.602	.000	–0.553	.000	–0.564	.000	–0.555	.000
45–49 years	–1.158	.000	–1.119	.000	–1.032	.000	–1.137	.000	–1.154	.000	–1.165	.000	–1.095	.000	–1.122	.000	–1.079	.000
50–54 years	–1.401	.000	–1.343	.000	–1.231	.000	–1.439	.000	–1.438	.000	–1.442	.000	–1.379	.000	–1.384	.000	–1.327	.000
Age difference between spouses																		
Wife older	0.035	.001	0.054	.000	0.051	.000	0.032	.000	0.041	.000	0.043	.000	0.021	.000	0.016	.000	0.039	.000
Husband 0–2 years older (ref.)																		
Husband 3–6 years older	–0.035	.001	–0.030	.001	–0.031	.000	–0.023	.000	–0.035	.000	–0.033	.000	–0.010	.014	–0.022	.000	–0.009	.023
Husband >6 years older	–0.098	.000	–0.134	.000	–0.109	.000	–0.092	.000	–0.103	.000	–0.098	.000	–0.078	.000	–0.096	.000	–0.067	.000
Children >4 years old in household																		
No (ref.)																		
Yes	0.269	.000	0.280	.000	0.221	.000	0.251	.000	0.263	.000	0.248	.000	0.250	.000	0.274	.000	0.261	.000
Husband household head																		
Yes (ref.)																		
No	–0.223	.000	–0.126	.000	–0.146	.000	–0.080	.000	–0.087	.000	–0.094	.000	–0.193	.000	–0.200	.000	–0.206	.000
Distance from parish of birth																		
Less than 10 km (ref.)																		
10–50 km	0.029	.001	0.010	.208	0.027	.000	0.026	.000	0.028	.000	0.029	.000	0.018	.000	0.023	.000	0.020	.000
More than 50 km	0.029	.001	–0.013	.084	–0.018	.006	0.069	.000	0.053	.000	0.030	.000	0.044	.000	0.038	.000	0.019	.000
Born abroad	0.001	.975	–0.077	.000	–0.043	.010	0.113	.000	0.068	.015	0.010	.704	0.001	.972	0.014	.389	–0.014	.333
Parish-Level Covariates																		
Female labor force rate																		
Low (1st quartile)	0.016	.130	0.031	.003	0.069	.000	0.010	.108	0.015	.034	0.029	.000	0.006	.271	0.008	.183	0.031	.000
Medium (2nd/3rd quartiles) (ref.)																		
High (4th quartile)	–0.040	.000	–0.035	.001	–0.037	.001	–0.039	.000	–0.050	.000	–0.064	.000	–0.027	.000	–0.032	.000	–0.042	.000
Education rate (teacher/child ratio)																		
Low (1st quartile)	–0.025	.011	0.001	.914	0.015	.171	–0.032	.000	0.002	.810	0.004	.554	–0.027	.000	0.000	.989	0.010	.068
Medium (2nd/3rd quartiles) (ref.)																		
High (4th quartile)	0.000	.993	–0.017	.103	–0.020	.084	–0.029	.000	–0.040	.000	–0.054	.000	–0.014	.016	–0.020	.001	–0.024	.000
Proportion employed in industry																		
Low (1st quartile)	0.040	.001	0.057	.000	0.022	.097	0.055	.000	0.047	.000	0.054	.000	0.037	.000	0.049	.000	0.031	.000
Medium (2nd/3rd quartiles) (ref.)																		
High (4th quartile)	0.003	.736	–0.014	.181	–0.020	.079	–0.007	.310	–0.015	.055	0.009	.247	0.001	.927	–0.001	.928	0.003	.663

Proportion of migrants born more than 100 km away and/or abroad																		
Low (1st quartile)	0.046	.000	0.026	.039	0.026	.049	0.020	.002	0.019	.009	0.016	.020	0.012	.069	0.011	.122	0.029	.000
Medium (2nd/3rd quartiles) (ref.)																		
High (4th quartile)	–0.063	.000	–0.037	.000	–0.036	.001	–0.063	.000	–0.045	.000	–0.044	.000	–0.051	.000	–0.04	.000	–0.053	.0000
Population density per km²																		
Less than 50 (ref.)																		
50–100	–0.025	.045	–0.008	.556	–0.012	.412	–0.010	.268	–0.038	.000	–0.044	.000	–0.009	.293	–0.003	.705	–0.003	.752
100–1,000	–0.016	.181	–0.012	.390	–0.015	.399	0.027	.189	0.000	.982	–0.014	.457	–0.001	.952	–0.002	.870	–0.008	.428
More than 1,000	–0.086	.000	–0.078	.001	–0.075	.029	–0.067	.264	–0.100	.133	–0.020	.727	–0.029	.229	–0.033	.153	–0.048	.015
Constant	1.261	.000	1.218	.000	1.151	.000	1.249	.000	1.247	.000	1.249	.000	1.238	.000	1.232	.000	1.187	.000
Number of Women	59,047		69,971		86,593		239,268		220,105		200,589		282,534		296,842		331,914	
Number of Parishes	2,408		2,409		2,416		2,422		2,426		2,428		2,435		2,435		2,435	
Spatial Autocorrelation Diagnostics																		
Moran's I dependent variable	0.059	.000	0.049	.000	0.049	.000	0.371	.000	0.353	.000	0.307	.000	0.276	.000	0.223	.000	0.210	.000
Moran's I residuals	0.018	.137	0.000	.940	–0.010	.722	0.186	.000	0.105	.000	0.137	.000	0.156	.000	0.126	.000	0.118	.000

Notes: The Moran's I is derived at the parish level; neighborhood is defined as the five nearest neighbors, with each neighbor given equal weight. Parishes with no observations are excluded from the calculation of the Moran's I prior to constructing the spatial weight matrices in which information on the five nearest neighboring parishes is stored.

Sources: Swedish National Archives et al. (2011a, 2011b, 2014), own calculations.

Table OA10 Model estimates for the number of children aged 0–4 per married woman aged 15–54 (without dummy for own children above age 4 in the household)

	1880		1890		1900	
	Coef.	*p* Val.	Coef.	*p* Val.	Coef.	*p* Val.
Individual-Level Covariates						
Age of woman						
15–19 years	–0.761	.000	–0.765	.000	–0.648	.000
20–24 years	–0.358	.000	–0.315	.000	–0.258	.000
25–29 years	–0.028	.000	–0.012	.001	0.035	.000
30–34 years (ref.)						
35–39 years	–0.158	.000	–0.170	.000	–0.177	.000
40–44 years	–0.504	.000	–0.520	.000	–0.515	.000
45–49 years	–1.047	.000	–1.066	.000	–1.038	.000
50–54 years	–1.343	.000	–1.344	.000	–1.300	.000
Age difference between spouses						
Wife older	0.001	.620	0.002	.561	0.017	.000
Husband 0–2 years older (ref.)						
Husband 3–6 years older	–0.007	.011	–0.016	.000	–0.010	.001
Husband >6 years older	–0.068	.000	–0.089	.000	–0.074	.000
Husband household head						
Yes (ref.)						
No	–0.183	.000	–0.174	.000	–0.185	.000
Socioeconomic status						
Elite (ref.)						
Farmers	0.019	.000	0.058	.000	0.098	.000
Skilled workers	0.054	.000	0.092	.000	0.107	.000
Lower-skilled workers	0.069	.000	0.109	.000	0.129	.000
Unskilled workers	0.004	.338	0.064	.000	0.100	.000
Others	–0.023	.000	–0.007	.221	0.035	.000
Distance from parish of birth						
Less than 10 km (ref.)						
10–50 km	0.024	.000	0.028	.000	0.030	.000
More than 50 km	0.042	.000	0.029	.000	0.010	.001
Born abroad	0.017	.170	–0.020	.085	–0.035	.001
Parish-Level Covariates						
Female labor force rate						
Low (1st quartile)	–0.003	.495	–0.001	.739	0.013	.004
Medium (2nd/3rd quartiles) (ref.)						
High (4th quartile)	–0.010	.045	–0.012	.011	–0.016	.001
Education rate (teacher/child ratio)						
Low (1st quartile)	–0.012	.005	0.007	.076	0.005	.219
Medium (2nd/3rd quartiles) (ref.)						
High (4th quartile)	–0.011	.018	–0.013	.003	–0.030	.000
Proportion employed in industry						
Low (1st quartile)	0.022	.000	0.017	.000	0.017	.001
Medium (2nd/3rd quartiles) (ref.)						
High (4th quartile)	0.000	.934	–0.009	.037	–0.006	.195

Proportion of migrants born more than 100 km away and/or abroad						
Low (1st quartile)	0.000	.973	–0.001	.916	–0.006	.220
Medium (2nd/3rd quartiles) (ref.)						
High (4th quartile)	–0.002	.714	0.002	.636	–0.008	.119
Population density per km²						
Less than 50 (ref.)						
50–100	–0.030	.000	–0.023	.001	–0.037	.000
100–1,000	–0.063	.000	–0.055	.000	–0.062	.000
More than 1,000	–0.074	.001	–0.088	.000	–0.113	.000
Regional dummies						
Less than 10 km from Stockholm (ref.)						
10–50 km from Stockholm	0.044	.271	0.028	.440	0.078	.027
50–100 km from Stockholm	0.003	.935	–0.024	.508	0.059	.089
100–150 km from Stockholm	0.024	.543	0.005	.884	0.078	.025
150–200 km from Stockholm	0.094	.016	0.061	.089	0.119	.001
Less than 10 km from Gothenburg	0.174	.000	0.176	.000	0.263	.000
10–50 km from Gothenburg	0.169	.000	0.179	.000	0.250	.000
50–100 km from Gothenburg	0.172	.000	0.156	.000	0.251	.000
Less than 10 km from Malmö	0.163	.000	0.088	.033	0.207	.000
10–50 km from Malmö	0.118	.003	0.049	.172	0.162	.000
50–100 km from Malmö	0.140	.000	0.097	.007	0.176	.000
Gotland	–0.107	.008	–0.135	.000	–0.018	.616
Southern Norrland & Kopparberg county	0.102	.009	0.090	.012	0.177	.000
Northern Norrland	0.274	.000	0.226	.000	0.307	.000
Other areas (central & southern Sweden)	0.174	.000	0.139	.000	0.222	.000
Constant	1.249	.000	1.254	.000	1.108	.000
Number of Women	580,849		586,198		619,096	
Number of Parishes	2,435		2,435		2,435	
Spatial Autocorrelation Diagnostics						
Moran's I dependent variable	0.465	.000	0.471	.000	0.414	.000
Moran's I residuals	0.192	.000	0.163	.000	0.142	.000

Note: The Moran's I is derived at the parish level; neighborhood is defined as the five nearest neighbors, with each neighbor given equal weight.

Sources: Swedish National Archives et al. (2011a, 2011b, 2014), own calculations.

Table OA11 Models by socioeconomic status: Estimates for the number of children aged 0–4 per married woman aged 15–54 (without dummy for own children above age 4 in the household)

	Elite						Farmers						Workers and Others					
	1880		1890		1900		1880		1890		1900		1880		1890		1900	
	Coef.	*p* Val.	Coef.	*p* Val.	Coef.	*p* Val.	Coef.	*p* Val.	Coef.	*p* Val.	Coef.	*p* Val.	Coef.	*p* Val.	Coef.	*p* Val.	Coef.	*p* Val.
Individual-Level Covariates																		
Age of woman																		
15–19 years	–0.774	.000	–0.850	.000	–0.518	.000	–0.907	.000	–0.883	.000	–0.812	.000	–0.692	.000	–0.719	.000	–0.625	.000
20–24 years	–0.379	.000	–0.298	.000	–0.251	.000	–0.369	.000	–0.327	.000	–0.297	.000	–0.345	.000	–0.310	.000	–0.241	.000
25–29 years	–0.009	.433	0.005	.635	0.050	.000	–0.014	.018	0.006	.379	0.040	.000	–0.037	.000	–0.020	.000	0.036	.000
30–34 years (ref.)																		
35–39 years	–0.198	.000	–0.207	.000	–0.195	.000	–0.172	.000	–0.179	.000	–0.184	.000	–0.140	.000	–0.157	.000	–0.172	.000
40–44 years	–0.574	.000	–0.568	.000	–0.546	.000	–0.517	.000	–0.540	.000	–0.545	.000	–0.482	.000	–0.497	.000	–0.494	.000
45–49 years	–1.078	.000	–1.045	.000	–0.966	.000	–1.072	.000	–1.093	.000	–1.106	.000	–1.023	.000	–1.057	.000	–1.018	.000
50–54 years	–1.329	.000	–1.279	.000	–1.173	.000	–1.380	.000	–1.384	.000	–1.388	.000	–1.321	.000	–1.336	.000	–1.280	.000
Age difference between spouses																		
Wife older	0.009	.410	0.028	.003	0.029	.000	0.012	.007	0.019	.000	0.023	.000	–0.006	.124	–0.014	.001	0.012	.003
Husband 0–2 years older (ref.)																		
Husband 3–6 years older	–0.027	.010	–0.018	.054	–0.023	.003	–0.014	.002	–0.026	.000	–0.026	.000	–0.001	.781	–0.010	.018	0.002	.655
Husband >6 years older	–0.077	.000	–0.115	.000	–0.099	.000	–0.077	.000	–0.091	.000	–0.091	.000	–0.066	.000	–0.085	.000	–0.057	.000
Husband household head																		
Yes (ref.)																		
No	–0.253	.000	–0.145	.000	–0.169	.000	–0.100	.000	–0.110	.000	–0.118	.000	–0.219	.000	–0.233	.000	–0.236	.000
Distance from parish of birth																		
Less than 10 km (ref.)																		
10–50 km	0.028	.001	0.014	.092	0.032	.000	0.026	.000	0.030	.000	0.035	.000	0.018	.000	0.025	.000	0.023	.000
More than 50 km	0.027	.002	–0.019	.014	–0.023	.000	0.068	.000	0.051	.000	0.031	.000	0.042	.000	0.035	.000	0.018	.000
Born abroad	–0.002	.938	–0.077	.000	–0.048	.005	0.113	.000	0.062	.030	–0.002	.949	–0.004	.817	0.004	.827	–0.027	.068
Parish-Level Covariates																		
Female labor force rate																		
Low (1st quartile)	0.009	.413	0.021	.048	0.055	.000	–0.006	.327	–0.001	.919	0.008	.139	–0.003	.611	–0.005	.346	0.015	.005
Medium (2nd/3rd quartiles) (ref.)																		
High (4th quartile)	–0.019	.042	–0.020	.046	–0.016	.150	–0.008	.216	–0.012	.091	–0.018	.009	–0.009	.079	–0.009	.116	–0.012	.023
Education rate (teacher/child ratio)																		
Low (1st quartile)	–0.006	.582	0.004	.700	0.011	.300	–0.011	.060	0.008	.172	–0.001	.905	–0.014	.004	0.005	.353	0.010	.041
Medium (2nd/3rd quartiles) (ref.)																		
High (4th quartile)	0.003	.789	–0.008	.406	–0.018	.115	–0.012	.045	–0.017	.011	–0.036	.000	–0.007	.146	–0.009	.101	–0.024	.000
Proportion employed in industry																		
Low (1st quartile)	0.022	.090	0.035	.007	–0.005	.690	0.023	.000	0.005	.479	0.020	.001	0.017	.003	0.023	.000	0.009	.141
Medium (2nd/3rd quartiles) (ref.)																		
High (4th quartile)	0.003	.801	–0.013	.182	–0.022	.044	0.005	.448	–0.010	.118	0.002	.709	0.001	.815	–0.005	.343	–0.005	.338
Proportion of migrants born more than 100 km away and/or abroad																		
Low (1st quartile)	0.036	.006	0.022	.099	0.015	.254	0.000	.968	0.001	.853	–0.016	.012	–0.007	.217	–0.008	.229	0.002	.745
Medium (2nd/3rd quartiles) (ref.)																		
High (4th quartile)	–0.033	.002	–0.018	.096	–0.019	.100	0.000	.991	0.009	.258	0.004	.575	–0.001	.799	–0.003	.579	–0.018	.001

Population density per km²																		
Less than 50 (ref.)																		
50–100	–0.042	.002	0.000	.988	–0.015	.330	–0.019	.050	–0.037	.000	–0.054	.000	–0.032	.000	–0.007	.384	–0.026	.000
100–1,000	–0.058	.000	–0.047	.001	–0.048	.005	–0.022	.267	–0.044	.020	–0.057	.001	–0.059	.000	–0.051	.000	–0.061	.000
More than 1,000	–0.054	.009	–0.107	.000	–0.114	.001	–0.103	.082	–0.119	.056	–0.022	.683	–0.072	.000	–0.077	.000	–0.111	.000
Regional dummies																		
Less than 10 km from Stockholm (ref.)																		
10–50 km from Stockholm	0.102	.000	0.067	.144	0.082	.212	0.039	.622	0.032	.704	0.213	.010	0.048	.172	0.033	.344	0.068	.029
50–100 km from Stockholm	0.062	.005	0.018	.666	0.087	.173	0.026	.741	–0.005	.955	0.204	.012	0.008	.814	–0.017	.611	0.045	.134
100–150 km from Stockholm	0.111	.000	0.044	.302	0.094	.143	0.032	.685	0.031	.706	0.219	.007	0.032	.342	0.014	.681	0.074	.013
150–200 km from Stockholm	0.144	.000	0.091	.036	0.117	.069	0.116	.142	0.094	.253	0.278	.001	0.099	.004	0.067	.049	0.111	.000
Less than 10 km from Gothenburg	0.195	.000	0.164	.001	0.234	.002	0.196	.027	0.270	.004	0.422	.000	0.160	.000	0.176	.000	0.269	.000
10–50 km from Gothenburg	0.217	.000	0.150	.002	0.247	.000	0.214	.007	0.242	.004	0.445	.000	0.145	.000	0.160	.000	0.203	.000
50–100 km from Gothenburg	0.197	.000	0.150	.001	0.198	.002	0.211	.008	0.206	.012	0.434	.000	0.164	.000	0.151	.000	0.232	.000
Less than 10 km from Malmö	0.177	.000	0.174	.001	0.190	.013	0.135	.112	0.009	.923	0.308	.001	0.188	.000	0.111	.006	0.212	.000
10–50 km from Malmö	0.192	.000	0.056	.198	0.126	.053	0.123	.119	0.073	.375	0.287	.000	0.136	.000	0.063	.068	0.174	.000
50–100 km from Malmö	0.154	.000	0.089	.044	0.133	.040	0.163	.038	0.135	.102	0.337	.000	0.155	.000	0.102	.003	0.167	.000
Gotland	0.059	.111	0.010	.855	0.047	.517	–0.136	.089	–0.151	.070	0.098	.236	–0.076	.038	–0.086	.021	0.012	.711
Southern Norrland & Kopparberg county	0.148	.000	0.113	.009	0.164	.011	0.128	.105	0.123	.135	0.338	.000	0.105	.002	0.100	.003	0.166	.000
Northern Norrland	0.251	.000	0.165	.000	0.267	.000	0.380	.000	0.342	.000	0.548	.000	0.187	.000	0.136	.000	0.215	.000
Other areas (central & southern Sweden)	0.207	.000	0.141	.001	0.192	.002	0.211	.007	0.184	.025	0.395	.000	0.170	.000	0.137	.000	0.211	.000
Constant	1.244	.000	1.281	.000	1.131	.000	1.247	.000	1.283	.000	1.078	.000	1.264	.000	1.319	.000	1.208	.000
Number of Women	59,047		69,971		86,593		239,268		220,105		200,589		282,534		296,842		331,914	
Number of Parishes	2,408		2,409		2,416		2,422		2,426		2,428		2,435		2,435		2,435	
Spatial Autocorrelation Diagnostics																		
Moran's I dependent variable	0.059	.000	0.049	.000	0.049	.000	0.371	.000	0.353	.000	0.307	.000	0.276	.000	0.223	.000	0.210	.000
Moran's I residuals	–0.002	.898	–0.014	.266	–0.020	.113	0.107	.000	0.048	.000	0.073	.000	0.074	.000	0.069	.000	0.060	.000

Notes: The Moran's I is derived at the parish level; neighborhood is defined as the five nearest neighbors, with each neighbor given equal weight. Parishes with no observations are excluded from the calculation of the Moran's I prior to constructing the spatial weight matrices in which information on the five nearest neighboring parishes is stored.

Sources: Swedish National Archives et al. (2011a, 2011b, 2014), own calculations.

Table OA12 Model estimates for the number of children aged 0–4 per married woman aged 15–54 (counties in Norrland excluded)

	1880		1890		1900	
	Coef.	*p* Val.	Coef.	*p* Val.	Coef.	*p* Val.
Individual-Level Covariates						
Age of woman						
15–19 years	–0.622	.000	–0.568	.000	–0.463	.000
20–24 years	–0.216	.000	–0.154	.000	–0.097	.000
25–29 years	0.051	.000	0.076	.000	0.113	.000
30–34 years (ref.)						
35–39 years	–0.204	.000	–0.215	.000	–0.223	.000
40–44 years	–0.565	.000	–0.577	.000	–0.573	.000
45–49 years	–1.108	.000	–1.117	.000	–1.087	.000
50–54 years	–1.396	.000	–1.383	.000	–1.335	.000
Age difference between spouses						
Wife older	0.026	.000	0.028	.000	0.042	.000
Husband 0–2 years older (ref.)						
Husband 3–6 years older	–0.016	.000	–0.028	.000	–0.020	.000
Husband >6 years older	–0.082	.000	–0.105	.000	–0.085	.000
Children >4 years old in household						
No (ref.)						
Yes	0.247	.000	0.261	.000	0.239	.000
Husband household head						
Yes (ref.)						
No	–0.153	.000	–0.141	.000	–0.149	.000
Socioeconomic status						
Elite (ref.)						
Farmers	0.004	.266	0.044	.000	0.081	.000
Skilled workers	0.049	.000	0.088	.000	0.100	.000
Lower-skilled workers	0.061	.000	0.102	.000	0.122	.000
Unskilled workers	0.005	.216	0.061	.000	0.095	.000
Others	–0.024	.000	–0.002	.746	0.039	.000
Distance from parish of birth						
Less than 10 km (ref.)						
10–50 km	0.023	.000	0.027	.000	0.029	.000
More than 50 km	0.043	.000	0.035	.000	0.020	.000
Born abroad	–0.012	.419	–0.050	.000	–0.047	.000
Parish-Level Covariates						
Female labor force rate						
Low (1st quartile)	0.000	.974	0.003	.575	0.013	.004
Medium (2nd/3rd quartiles) (ref.)						
High (4th quartile)	–0.007	.121	–0.009	.059	–0.014	.003
Education rate (teacher/child ratio)						
Low (1st quartile)	–0.006	.164	0.006	.175	0.007	.094
Medium (2nd/3rd quartiles) (ref.)						
High (4th quartile)	–0.007	.126	–0.007	.091	–0.022	.000
Proportion employed in industry						
Low (1st quartile)	0.011	.019	0.012	.012	0.007	.146
Medium (2nd/3rd quartiles) (ref.)						
High (4th quartile)	0.001	.830	–0.008	.064	–0.008	.067

Proportion of migrants born more than 100 km away and/or abroad						
Low (1st quartile)	0.002	.624	0.002	.666	–0.001	.790
Medium (2nd/3rd quartiles) (ref.)						
High (4th quartile)	0.001	.841	0.003	.547	–0.008	.116
Population density per km²						
Less than 50 (ref.)						
50–100	–0.029	.000	–0.024	.000	–0.036	.000
100–1,000	–0.062	.000	–0.054	.000	–0.060	.000
More than 1,000	–0.068	.001	–0.086	.000	–0.108	.000
Regional dummies						
Less than 10 km from Stockholm (ref.)						
10–50 km from Stockholm	0.034	.336	0.022	.503	0.070	.033
50–100 km from Stockholm	–0.002	.953	–0.025	.447	0.054	.094
100–150 km from Stockholm	0.015	.670	–0.004	.910	0.069	.032
150–200 km from Stockholm	0.088	.012	0.055	.093	0.111	.001
Less than 10 km from Gothenburg	0.167	.000	0.171	.000	0.256	.000
10–50 km from Gothenburg	0.168	.000	0.178	.000	0.249	.000
50–100 km from Gothenburg	0.171	.000	0.157	.000	0.253	.000
Less than 10 km from Malmö	0.161	.000	0.082	.030	0.196	.000
10–50 km from Malmö	0.116	.001	0.049	.134	0.157	.000
50–100 km from Malmö	0.140	.000	0.098	.003	0.173	.000
Gotland	–0.113	.002	–0.129	.000	–0.017	.620
Southern Norrland & Kopparberg county	0.082	.025	0.060	.079	0.152	.000
Northern Norrland	0.170	.000	0.135	.000	0.220	.000
Constant	1.099	.000	1.084	.000	0.955	.000
Number of Women	495,985		490,188		511,752	
Number of Parishes	2,213		2,213		2,213	
Spatial Autocorrelation Diagnostics						
Moran's I dependent variable	0.424	.000	0.422	.000	0.369	.000
Moran's I residuals	0.186	.000	0.148	.000	0.137	.000

Notes: The excluded counties in Norrland are Gävleborg, Västernorrland, Jämtland, Västerbotten, and Norrbotten. The Moran's I is derived at the parish level; neighborhood is defined as the five nearest neighbors, with each neighbor given equal weight.

Sources: Swedish National Archives et al. (2011a, 2011b, 2014), own calculations.

Table OA13 Models by socioeconomic status: Estimates for the number of children aged 0–4 per married woman aged 15–54 (counties in Norrland excluded)

	Elite						Farmers						Workers and Others					
	1880		1890		1900		1880		1890		1900		1880		1890		1900	
	Coef.	*p* Val.	Coef.	*p* Val.	Coef.	*p* Val.	Coef.	*p* Val.	Coef.	*p* Val.	Coef.	*p* Val.	Coef.	*p* Val.	Coef.	*p* Val.	Coef.	*p* Val.
Individual-Level Covariates																		
Age of woman																		
15–19 years	–0.645	.000	–0.632	.000	–0.395	.000	–0.751	.000	–0.676	.000	–0.652	.000	–0.553	.000	–0.528	.000	–0.423	.000
20–24 years	–0.234	.000	–0.131	.000	–0.120	.000	–0.220	.000	–0.171	.000	–0.150	.000	–0.207	.000	–0.147	.000	–0.068	.000
25–29 years	0.073	.000	0.102	.000	0.124	.000	0.068	.000	0.086	.000	0.100	.000	0.040	.000	0.070	.000	0.123	.000
30–34 years (ref.)																		
35–39 years	–0.253	.000	–0.256	.000	–0.237	.000	–0.211	.000	–0.217	.000	–0.232	.000	–0.190	.000	–0.207	.000	–0.218	.000
40–44 years	–0.650	.000	–0.630	.000	–0.599	.000	–0.569	.000	–0.586	.000	–0.602	.000	–0.548	.000	–0.561	.000	–0.554	.000
45–49 years	–1.157	.000	–1.108	.000	–1.018	.000	–1.126	.000	–1.136	.000	–1.153	.000	–1.089	.000	–1.114	.000	–1.072	.000
50–54 years	–1.400	.000	–1.331	.000	–1.214	.000	–1.428	.000	–1.419	.000	–1.426	.000	–1.375	.000	–1.376	.000	–1.315	.000
Age difference between spouses																		
Wife older	0.040	.000	0.052	.000	0.052	.000	0.033	.000	0.045	.000	0.046	.000	0.020	.000	0.016	.000	0.039	.000
Husband 0–2 years older (ref.)																		
Husband 3–6 years older	–0.038	.000	–0.036	.000	–0.038	.000	–0.021	.000	–0.035	.000	–0.028	.000	–0.011	.011	–0.023	.000	–0.012	.007
Husband >6 years older	–0.102	.000	–0.139	.000	–0.113	.000	–0.091	.000	–0.104	.000	–0.098	.000	–0.079	.000	–0.101	.000	–0.070	.000
Children >4 years old in household																		
No (ref.)																		
Yes	0.263	.000	0.270	.000	0.212	.000	0.247	.000	0.252	.000	0.230	.000	0.245	.000	0.266	.000	0.253	.000
Husband household head																		
Yes (ref.)																		
No	–0.224	.000	–0.112	.000	–0.138	.000	–0.091	.000	–0.082	.000	–0.086	.000	–0.189	.000	–0.196	.000	–0.196	.000
Distance from parish of birth																		
Less than 10 km (ref.)																		
10–50 km	0.025	.005	0.013	.115	0.031	.000	0.027	.000	0.030	.000	0.031	.000	0.018	.000	0.025	.000	0.026	.000
More than 50 km	0.031	.001	–0.008	.325	–0.010	.152	0.065	.000	0.049	.000	0.026	.004	0.044	.000	0.043	.000	0.030	.000
Born abroad	–0.015	.536	–0.092	.000	–0.038	.035	0.082	.013	0.032	.369	–0.033	.353	–0.054	.026	–0.018	.399	–0.049	.007
Parish-Level Covariates																		
Female labor force rate																		
Low (1st quartile)	0.006	.602	0.023	.053	0.056	.000	0.004	.468	0.007	.268	0.010	.104	–0.009	.135	–0.006	.284	0.012	.046
Medium (2nd/3rd quartiles) (ref.)																		
High (4th quartile)	–0.014	.177	–0.012	.284	–0.011	.372	–0.008	.200	–0.014	.050	–0.016	.019	–0.006	.290	–0.003	.543	–0.010	.055
Education rate (teacher/child ratio)																		
Low (1st quartile)	–0.009	.429	0.006	.569	0.021	.083	–0.004	.441	0.004	.551	–0.001	.902	–0.006	.246	0.008	.146	0.014	.009
Medium (2nd/3rd quartiles) (ref.)																		
High (4th quartile)	0.001	.905	–0.002	.855	–0.006	.639	–0.009	.135	–0.009	.168	–0.030	.000	–0.004	.431	–0.004	.416	–0.018	.001
Proportion employed in industry																		
Low (1st quartile)	0.006	.673	0.023	.109	0.005	.720	0.010	.107	–0.001	.860	0.003	.593	0.014	.028	0.020	.003	0.003	.651
Medium (2nd/3rd quartiles) (ref.)																		
High (4th quartile)	–0.001	.948	–0.020	.068	–0.022	.068	0.006	.347	–0.011	.103	–0.003	.685	0.002	.711	–0.003	.603	–0.006	.243

Proportion of migrants born more than 100 km away and/or abroad																		
Low (1st quartile)	0.035	.010	0.018	.181	0.011	.437	0.001	.825	0.003	.632	–0.011	.061	–0.005	.428	–0.006	.379	0.006	.349
Medium (2nd/3rd quartiles) (ref.)																		
High (4th quartile)	–0.035	.003	–0.024	.059	–0.029	.032	0.011	.171	0.015	.086	0.011	.159	–0.003	.679	–0.001	.817	–0.020	.001
Population density per km²																		
Less than 50 (ref.)																		
50–100	–0.037	.010	0.000	.977	–0.011	.526	–0.021	.022	–0.038	.000	–0.053	.000	–0.028	.000	–0.007	.400	–0.023	.002
100–1,000	–0.054	.000	–0.039	.018	–0.039	.044	–0.034	.075	–0.035	.062	–0.041	.019	–0.053	.000	–0.048	.000	–0.053	.000
More than 1,000	–0.046	.026	–0.096	.000	–0.098	.006	–0.095	.089	–0.096	.103	–0.018	.732	–0.061	.001	–0.073	.000	–0.099	.000
Regional dummies																		
Less than 10 km from Stockholm (ref.)																		
10–50 km from Stockholm	0.085	.003	0.050	.333	0.077	.274	0.027	.721	0.026	.745	0.188	.019	0.043	.209	0.031	.347	0.064	.023
50–100 km from Stockholm	0.048	.029	0.005	.918	0.078	.252	0.023	.755	–0.006	.943	0.189	.017	0.002	.941	–0.016	.618	0.043	.116
100–150 km from Stockholm	0.092	.000	0.022	.659	0.083	.227	0.024	.741	0.023	.767	0.201	.012	0.023	.474	0.007	.837	0.067	.014
150–200 km from Stockholm	0.117	.000	0.062	.213	0.096	.163	0.124	.091	0.097	.220	0.267	.001	0.087	.009	0.061	.056	0.103	.000
Less than 10 km from Gothenburg	0.183	.000	0.161	.005	0.225	.006	0.198	.016	0.258	.004	0.391	.000	0.150	.000	0.175	.000	0.261	.000
10–50 km from Gothenburg	0.209	.000	0.137	.010	0.235	.001	0.218	.003	0.241	.002	0.437	.000	0.142	.000	0.162	.000	0.204	.000
50–100 km from Gothenburg	0.189	.000	0.142	.005	0.189	.006	0.215	.003	0.208	.008	0.428	.000	0.159	.000	0.156	.000	0.235	.000
Less than 10 km from Malmö	0.161	.000	0.147	.013	0.174	.031	0.152	.055	0.011	.896	0.292	.001	0.175	.000	0.102	.007	0.199	.000
10–50 km from Malmö	0.176	.000	0.041	.411	0.111	.109	0.134	.069	0.081	.304	0.279	.000	0.125	.000	0.061	.060	0.169	.000
50–100 km from Malmö	0.140	.000	0.074	.140	0.122	.078	0.171	.020	0.141	.074	0.328	.000	0.149	.000	0.101	.002	0.164	.000
Gotland	0.037	.316	0.012	.841	0.041	.595	–0.141	.059	–0.148	.063	0.088	.276	–0.081	.022	–0.078	.025	0.018	.566
Kopparberg county	0.150	.000	0.085	.129	0.100	.177	0.090	.231	0.073	.359	0.291	.000	0.113	.001	0.104	.002	0.165	.000
Other areas (central & southern Sweden)	0.193	.000	0.126	.009	0.180	.008	0.211	.004	0.181	.021	0.385	.000	0.163	.000	0.135	.000	0.209	.000
Constant	1.108	.000	1.127	.000	1.002	.000	1.074	.000	1.104	.000	0.933	.000	1.116	.000	1.137	.000	1.030	.000
Number of Women	52,686		61,630		75,525		201,284		178,211		159,002		242,015		250,347		277,225	
Number of Parishes	2,187		2,188		2,194		2,203		2,206		2,207		2,213		2,213		2,213	
Spatial Autocorrelation Diagnostics																		
Moran's I dependent variable	0.061	.000	0.042	.001	0.034	.007	0.333	.000	0.316	.000	0.268	.000	0.264	.000	0.201	.000	0.194	.000
Moran's I residuals	–0.005	.716	–0.013	.320	–0.025	.060	0.114	.000	0.047	.000	0.058	.000	0.060	.000	0.049	.000	0.065	.000

Notes: The excluded counties in Norrland are Gävleborg, Västernorrland, Jämtland, Västerbotten, and Norrbotten. The Moran's I is measured at parish level; neighborhood is defined as the five nearest neighbors. Parishes with no observations are excluded from the calculation of the Moran's I prior to constructing the spatial weight matrices in which information on the five nearest neighboring parishes is stored. As a result, each region has the five nearest neighbors with at least one woman each.

Sources: Swedish National Archives et al. (2011a, 2011b, 2014), own calculations.

Table OA14 Model estimates for the number of children aged 0 per married woman aged 15–54

	1880		1890		1900	
	Coef.	*p* Val.	Coef.	*p* Val.	Coef.	*p* Val.
Individual-Level Covariates						
Age of woman						
15–19 years	0.091	.000	0.085	.000	0.155	.000
20–24 years	0.091	.000	0.098	.000	0.124	.000
25–29 years	0.050	.000	0.056	.000	0.064	.000
30–34 years (ref.)						
35–39 years	–0.056	.000	–0.060	.000	–0.056	.000
40–44 years	–0.153	.000	–0.156	.000	–0.153	.000
45–49 years	–0.270	.000	–0.270	.000	–0.261	.000
50–54 years	–0.296	.000	–0.291	.000	–0.282	.000
Age difference between spouses						
Wife older	0.013	.000	0.013	.000	0.014	.000
Husband 0–2 years older (ref.)						
Husband 3–6 years older	–0.010	.000	–0.012	.000	–0.010	.000
Husband >6 years older	–0.026	.000	–0.031	.000	–0.025	.000
Children >4 years old in household						
No (ref.)						
Yes	0.012	.000	0.009	.000	0.008	.000
Husband household head						
Yes (ref.)						
No	–0.018	.000	–0.018	.000	–0.025	.000
Socioeconomic status						
Elite (ref.)						
Farmers	0.003	.079	0.013	.000	0.020	.000
Skilled workers	0.014	.000	0.023	.000	0.020	.000
Lower-skilled workers	0.019	.000	0.023	.000	0.025	.000
Unskilled workers	0.003	.149	0.017	.000	0.020	.000
Others	–0.005	.059	0.003	.320	0.009	.000
Distance from parish of birth						
Less than 10 km (ref.)						
10–50 km	0.005	.000	0.008	.000	0.005	.000
More than 50 km	0.012	.000	0.008	.000	0.003	.029
Born abroad	–0.012	.055	–0.013	.018	–0.017	.000
Parish-Level Covariates						
Female labor force rate						
Low (1st quartile)	–0.003	.030	–0.002	.295	0.000	.790
Medium (2nd/3rd quartiles) (ref.)						
High (4th quartile)	–0.001	.441	0.000	.915	–0.003	.052
Education rate (teacher/child ratio)						
Low (1st quartile)	–0.001	.541	0.001	.571	0.003	.036
Medium (2nd/3rd quartiles) (ref.)						
High (4th quartile)	–0.002	.165	–0.005	.004	–0.004	.006
Proportion employed in industry						
Low (1st quartile)	0.004	.021	0.006	.000	–0.002	.300
Medium (2nd/3rd quartiles) (ref.)						
High (4th quartile)	–0.001	.614	–0.001	.436	0.001	.323

Proportion of migrants born more than 100 km away and/or abroad						
Low (1st quartile)	0.002	.285	0.000	.994	0.005	.002
Medium (2nd/3rd quartiles) (ref.)						
High (4th quartile)	–0.005	.001	–0.003	.092	–0.006	.000
Population density per km²						
Less than 50 (ref.)						
50–100	–0.001	.779	–0.003	.189	–0.008	.000
100–1,000	–0.003	.327	–0.010	.000	–0.011	.000
More than 1,000	–0.008	.118	–0.013	.007	–0.018	.000
Regional dummies						
Less than 10 km from Stockholm (ref.)						
10–50 km from Stockholm	–0.001	.949	–0.006	.519	0.014	.040
50–100 km from Stockholm	–0.007	.450	–0.010	.279	0.002	.743
100–150 km from Stockholm	–0.002	.777	–0.007	.437	0.010	.118
150–200 km from Stockholm	0.008	.390	0.006	.544	0.016	.009
Less than 10 km from Gothenburg	0.027	.006	0.038	.000	0.056	.000
10–50 km from Gothenburg	0.026	.005	0.039	.000	0.046	.000
50–100 km from Gothenburg	0.025	.005	0.035	.000	0.052	.000
Less than 10 km from Malmö	0.022	.040	0.010	.363	0.034	.000
10–50 km from Malmö	0.018	.049	0.007	.446	0.024	.000
50–100 km from Malmö	0.021	.020	0.019	.048	0.029	.000
Gotland	–0.027	.005	–0.030	.004	–0.010	.195
Southern Norrland & Kopparberg county	0.012	.173	0.018	.058	0.032	.000
Northern Norrland	0.042	.000	0.051	.000	0.065	.000
Other areas (central & southern Sweden)	0.027	.002	0.032	.000	0.041	.000
Constant	0.270	.000	0.259	.000	0.236	.000
Number of Women	580,849		586,198		619,096	
Number of Parishes	2,435		2,435		2,435	
Spatial Autocorrelation Diagnostics						
Moran's I dependent variable	0.192	.000	0.266	.000	0.212	.000
Moran's I residuals	0.073	.000	0.073	.000	0.060	.000

Notes: The Moran's I is derived at the parish level; neighborhood is defined as the five nearest neighbors, with each neighbor given equal weight. For this specification, we do not present models for the three SES groups, as the outcomes for single SES became too unstable if only births from 1 year were considered at this high level of geographic detail.

Sources: Swedish National Archives et al. (2011a, 2011b, 2014), own calculations.

Table OA15 Model estimates for the number of children aged 0–4 per married woman aged 15–54 (conditional autoregressive model)

	1880			1890			1900		
	Posterior Marginals			Posterior Marginals			Posterior Marginals		
	Mean	0.025	0.975	Mean	0.025	0.975	Mean	0.025	0.975
Individual-Level Covariates									
Age of woman									
15–19 years	–0.605	–0.639	–0.570	–0.589	–0.625	–0.554	–0.479	–0.510	–0.448
20–24 years	–0.213	–0.224	–0.202	–0.150	–0.161	–0.138	–0.101	–0.111	–0.092
25–29 years	0.055	0.047	0.063	0.080	0.072	0.088	0.120	0.112	0.127
30–34 years (ref.)									
35–39 years	–0.207	–0.215	–0.199	–0.218	–0.226	–0.211	–0.221	–0.228	–0.214
40–44 years	–0.571	–0.579	–0.564	–0.585	–0.592	–0.577	–0.576	–0.583	–0.569
45–49 years	–1.117	–1.125	–1.109	–1.131	–1.139	–1.123	–1.100	–1.107	–1.093
50–54 years	–1.404	–1.413	–1.396	–1.397	–1.405	–1.389	–1.353	–1.360	–1.345
Age difference between spouses									
Wife older	0.026	0.019	0.032	0.028	0.021	0.034	0.040	0.035	0.046
Husband 0–2 years older (ref.)									
Husband 3–6 years older	–0.017	–0.023	–0.010	–0.027	–0.034	–0.021	–0.019	–0.025	–0.013
Husband >6 years older	–0.083	–0.090	–0.077	–0.103	–0.109	–0.096	–0.083	–0.089	–0.078
Children >4 years old in household									
No (ref.)									
Yes	0.253	0.247	0.258	0.271	0.265	0.277	0.252	0.247	0.257
Husband household head									
Yes (ref.)									
No	–0.152	–0.164	–0.139	–0.144	–0.157	–0.132	–0.155	–0.167	–0.143
Socioeconomic status									
Elite (ref.)									
Farmers	0.011	0.003	0.019	0.049	0.042	0.057	0.086	0.079	0.093
Skilled workers	0.050	0.040	0.060	0.085	0.076	0.094	0.096	0.088	0.104
Lower-skilled workers	0.061	0.051	0.072	0.098	0.089	0.108	0.115	0.107	0.122
Unskilled workers	0.007	–0.002	0.015	0.060	0.052	0.068	0.091	0.084	0.098
Others	–0.022	–0.034	–0.010	–0.002	–0.014	0.010	0.034	0.023	0.044
Distance from parish of birth									
Less than 10 km (ref.)									
10–50 km	0.024	0.019	0.029	0.028	0.023	0.033	0.029	0.024	0.034
More than 50 km	0.049	0.041	0.056	0.035	0.028	0.042	0.014	0.008	0.020
Born abroad	0.013	–0.015	0.041	–0.017	–0.041	0.007	–0.030	–0.050	–0.010

Parish-Level Covariates									
Female labor force rate									
Low (1st quartile)	–0.002	–0.010	0.005	0.005	–0.002	0.012	0.003	–0.005	0.011
Medium (2nd/3rd quartiles) (ref.)									
High (4th quartile)	–0.009	–0.018	–0.001	–0.012	–0.020	–0.004	–0.010	–0.018	–0.001
Education rate (teacher/child ratio)									
Low (1st quartile)	–0.006	–0.013	0.001	0.007	0.001	0.014	0.000	–0.008	0.007
Medium (2nd/3rd quartiles) (ref.)									
High (4th quartile)	–0.008	–0.015	0.000	–0.004	–0.011	0.003	–0.022	–0.031	–0.014
Proportion employed in industry									
Low (1st quartile)	0.014	0.005	0.023	0.008	–0.001	0.016	0.010	0.001	0.019
Medium (2nd/3rd quartiles) (ref.)									
High (4th quartile)	0.001	–0.007	0.008	–0.012	–0.019	–0.005	–0.011	–0.019	–0.003
Proportion of migrants born more than 100 km away and/or abroad									
Low (1st quartile)	0.004	–0.004	0.012	0.003	–0.006	0.012	–0.002	–0.012	0.007
Medium (2nd/3rd quartiles) (ref.)									
High (4th quartile)	–0.002	–0.011	0.007	–0.002	–0.010	0.006	–0.010	–0.019	–0.001
Population density per km²									
Less than 50 (ref.)									
50–100	–0.026	–0.038	–0.014	–0.015	–0.027	–0.004	–0.032	–0.045	–0.020
100–1,000	–0.056	–0.071	–0.040	–0.041	–0.054	–0.029	–0.054	–0.069	–0.038
More than 1,000	–0.076	–0.105	–0.047	–0.089	–0.112	–0.066	–0.118	–0.149	–0.087
Regional dummies									
Less than 10 km from Stockholm (ref.)									
10–50 km from Stockholm	0.058	–0.006	0.122	0.008	–0.046	0.062	0.065	–0.011	0.141
50–100 km from Stockholm	0.035	–0.035	0.105	–0.031	–0.091	0.029	0.075	–0.008	0.158
100–150 km from Stockholm	0.057	–0.015	0.130	–0.008	–0.070	0.054	0.083	–0.002	0.168
150–200 km from Stockholm	0.104	0.031	0.178	0.032	–0.031	0.095	0.113	0.027	0.198
Less than 10 km from Gothenburg	0.188	0.094	0.283	0.137	0.055	0.218	0.248	0.137	0.360
10–50 km from Gothenburg	0.192	0.114	0.271	0.121	0.053	0.189	0.204	0.113	0.294
50–100 km from Gothenburg	0.186	0.110	0.262	0.086	0.020	0.152	0.180	0.092	0.268
Less than 10 km from Malmö	0.219	0.116	0.322	0.106	0.017	0.195	0.210	0.090	0.329
10–50 km from Malmö	0.154	0.068	0.240	0.048	–0.026	0.123	0.168	0.068	0.267
50–100 km from Malmö	0.155	0.074	0.236	0.054	–0.017	0.124	0.143	0.049	0.238
Gotland	–0.096	–0.170	–0.022	–0.169	–0.233	–0.104	–0.054	–0.139	0.032
Southern Norrland & Kopparberg county	0.102	0.025	0.180	0.041	–0.026	0.108	0.134	0.043	0.225
Northern Norrland	0.140	0.049	0.232	0.065	–0.013	0.144	0.194	0.088	0.300
Other areas (central & southern Sweden)	0.165	0.092	0.239	0.068	0.004	0.131	0.152	0.067	0.238

Constant	1.083	1.010	1.155	1.126	1.064	1.188	0.989	0.906	1.073
Number of Women	580,849			586,198			619,096		
Number of Parishes	2,435			2,435			2,435		

Sources: Swedish National Archives et al. (2011a, 2011b, 2014), own calculations.

Table OA16 Models by socioeconomic status: Estimates for the number of children aged 0–4 per married woman aged 15–54 (conditional autoregressive model)

Elite

	1880			1890			1900		
	Posterior Marginals			Posterior Marginals			Posterior Marginals		
	Mean	0.025	0.975	Mean	0.025	0.975	Mean	0.025	0.975
Individual-Level Covariates									
Age of woman									
15–19 years	–0.619	–0.729	–0.510	–0.674	–0.794	–0.555	–0.392	–0.503	–0.280
20–24 years	–0.235	–0.268	–0.202	–0.138	–0.170	–0.106	–0.125	–0.153	–0.097
25–29 years	0.074	0.050	0.098	0.101	0.079	0.123	0.123	0.103	0.142
30–34 years (ref.)									
35–39 years	–0.250	–0.272	–0.228	–0.262	–0.282	–0.242	–0.240	–0.258	–0.223
40–44 years	–0.650	–0.674	–0.627	–0.640	–0.661	–0.620	–0.607	–0.626	–0.589
45–49 years	–1.158	–1.182	–1.133	–1.119	–1.140	–1.098	–1.033	–1.052	–1.014
50–54 years	–1.401	–1.427	–1.375	–1.343	–1.366	–1.320	–1.231	–1.251	–1.211
Age difference between spouses									
Wife older	0.036	0.015	0.056	0.054	0.035	0.072	0.052	0.036	0.068
Husband 0–2 years older (ref.)									
Husband 3–6 years older	–0.036	–0.056	–0.015	–0.030	–0.048	–0.012	–0.032	–0.047	–0.017
Husband >6 years older	–0.099	–0.118	–0.080	–0.136	–0.154	–0.119	–0.110	–0.125	–0.095
Children >4 years old in household									
No (ref.)									
Yes	0.266	0.249	0.283	0.280	0.264	0.295	0.223	0.210	0.236
Husband household head									
Yes (ref.)									
No	–0.222	–0.284	–0.161	–0.122	–0.176	–0.068	–0.144	–0.196	–0.092
Distance from parish of birth									
Less than 10 km (ref.)									
10–50 km	0.027	0.010	0.044	0.012	–0.004	0.028	0.031	0.017	0.045
More than 50 km	0.035	0.018	0.052	–0.012	–0.027	0.003	–0.018	–0.031	–0.005
Born abroad	–0.010	–0.055	0.035	–0.081	–0.121	–0.042	–0.047	–0.080	–0.014
Parish-Level Covariates									
Female labor force rate									
Low (1st quartile)	0.003	–0.021	0.025	0.022	0.002	0.042	0.048	0.029	0.067
Medium (2nd/3rd quartiles) (ref.)									
High (4th quartile)	–0.021	–0.041	–0.000	–0.018	–0.037	0.001	–0.024	–0.042	–0.006

Education rate (teacher/child ratio)									
Low (1st quartile)	–0.005	–0.026	0.015	0.003	–0.015	0.021	0.010	–0.008	0.028
Medium (2nd/3rd quartiles) (ref.)									
High (4th quartile)	0.005	–0.015	0.025	–0.006	–0.024	0.012	–0.012	–0.030	0.006
Proportion employed in industry									
Low (1st quartile)	0.024	–0.003	0.051	0.035	0.010	0.060	0.003	–0.020	0.025
Medium (2nd/3rd quartiles) (ref.)									
High (4th quartile)	–0.001	–0.022	0.020	–0.015	–0.034	0.004	–0.019	–0.037	–0.001
Proportion of migrants born more than 100 km away and/or abroad									
Low (1st quartile)	0.035	0.008	0.062	0.026	0.000	0.052	0.015	–0.009	0.038
Medium (2nd/3rd quartiles) (ref.)									
High (4th quartile)	–0.032	–0.055	–0.010	–0.019	–0.039	0.002	–0.018	–0.037	0.001
Population density per km²									
Less than 50 (ref.)									
50–100	–0.033	–0.062	–0.005	–0.001	–0.028	0.026	–0.022	–0.047	0.003
100–1,000	–0.047	–0.075	–0.018	–0.041	–0.066	–0.016	–0.052	–0.076	–0.027
More than 1,000	–0.048	–0.095	–0.002	–0.107	–0.149	–0.067	–0.132	–0.171	–0.094
Regional dummies									
Less than 10 km from Stockholm (ref.)									
10–50 km from Stockholm	0.074	–0.008	0.154	0.033	–0.045	0.106	0.063	–0.017	0.144
50–100 km from Stockholm	0.021	–0.067	0.105	–0.011	–0.098	0.065	0.071	–0.020	0.164
100–150 km from Stockholm	0.064	–0.030	0.154	0.012	–0.076	0.093	0.069	–0.026	0.166
150–200 km from Stockholm	0.098	0.001	0.191	0.064	–0.025	0.148	0.092	–0.007	0.191
Less than 10 km from Gothenburg	0.177	0.059	0.295	0.130	0.025	0.247	0.189	0.060	0.323
10–50 km from Gothenburg	0.184	0.071	0.296	0.125	0.024	0.224	0.201	0.088	0.315
50–100 km from Gothenburg	0.159	0.053	0.262	0.116	0.020	0.207	0.138	0.031	0.246
Less than 10 km from Malmö	0.162	0.027	0.298	0.127	0.018	0.239	0.096	–0.049	0.242
10–50 km from Malmö	0.137	0.017	0.252	0.024	–0.083	0.127	0.053	–0.070	0.176
50–100 km from Malmö	0.092	–0.024	0.202	0.057	–0.046	0.156	0.040	–0.076	0.156
Gotland	0.005	–0.109	0.115	–0.005	–0.113	0.100	–0.006	–0.121	0.109
Southern Norrland & Kopparberg county	0.123	0.018	0.226	0.087	–0.002	0.175	0.096	–0.010	0.203
Northern Norrland	0.231	0.097	0.364	0.148	0.038	0.260	0.175	0.040	0.312
Other areas (central & southern Sweden)	0.158	0.059	0.253	0.106	0.017	0.191	0.126	0.025	0.225
Constant	1.129	1.035	1.227	1.138	1.053	1.228	1.053	0.955	1.151
Number of Women	52,686			61,630			75,525		
Number of Parishes	2,187			2,188			2,194		

Table OA16 (continued)

Farmers

	1880			1890			1900		
	Posterior Marginals			Posterior Marginals			Posterior Marginals		
	Mean	0.025	0.975	Mean	0.025	0.975	Mean	0.025	0.975
Individual-Level Covariates									
Age of woman									
15–19 years	–0.740	–0.798	–0.682	–0.714	–0.784	–0.643	–0.649	–0.732	–0.566
20–24 years	–0.215	–0.232	–0.198	–0.163	–0.183	–0.143	–0.144	–0.166	–0.123
25–29 years	0.071	0.059	0.083	0.094	0.081	0.106	0.122	0.107	0.136
30–34 years (ref.)									
35–39 years	–0.217	–0.227	–0.207	–0.223	–0.234	–0.212	–0.226	–0.238	–0.214
40–44 years	–0.578	–0.589	–0.568	–0.599	–0.610	–0.588	–0.602	–0.614	–0.590
45–49 years	–1.137	–1.147	–1.126	–1.154	–1.165	–1.143	–1.165	–1.178	–1.153
50–54 years	–1.439	–1.450	–1.428	–1.438	–1.449	–1.427	–1.443	–1.455	–1.430
Age difference between spouses									
Wife older	0.031	0.023	0.040	0.040	0.031	0.050	0.042	0.032	0.052
Husband 0–2 years older (ref.)									
Husband 3–6 years older	–0.023	–0.032	–0.015	–0.036	–0.045	–0.027	–0.033	–0.043	–0.023
Husband >6 years older	–0.093	–0.102	–0.084	–0.105	–0.114	–0.095	–0.100	–0.110	–0.090
Children >4 years old in household									
No (ref.)									
Yes	0.252	0.243	0.260	0.263	0.254	0.272	0.248	0.239	0.258
Husband household head									
Yes (ref.)									
No	–0.074	–0.102	–0.046	–0.079	–0.103	–0.055	–0.088	–0.114	–0.062
Distance from parish of birth									
Less than 10 km (ref.)									
10–50 km	0.027	0.020	0.035	0.030	0.022	0.037	0.034	0.026	0.042
More than 50 km	0.068	0.053	0.084	0.054	0.039	0.069	0.033	0.019	0.048
Born abroad	0.109	0.053	0.165	0.062	0.007	0.117	0.000	–0.053	0.053
Parish-Level Covariates									
Female labor force rate									
Low (1st quartile)	0.001	–0.010	0.011	0.006	–0.004	0.017	–0.001	–0.012	0.010
Medium (2nd/3rd quartiles) (ref.)									
High (4th quartile)	–0.006	–0.018	0.007	–0.012	–0.026	0.001	–0.005	–0.019	0.009

Education rate (teacher/child ratio)									
Low (1st quartile)	–0.004	–0.014	0.006	0.007	–0.003	0.017	–0.007	–0.017	0.004
Medium (2nd/3rd quartiles) (ref.)									
High (4th quartile)	–0.007	–0.017	0.004	–0.007	–0.019	0.004	–0.028	–0.041	–0.016
Proportion employed in industry									
Low (1st quartile)	0.016	0.005	0.028	–0.001	–0.012	0.011	0.011	–0.001	0.024
Medium (2nd/3rd quartiles) (ref.)									
High (4th quartile)	0.006	–0.006	0.017	–0.011	–0.022	0.001	–0.004	–0.016	0.009
Proportion of migrants born more than 100 km away and/or abroad									
Low (1st quartile)	0.001	–0.010	0.012	0.005	–0.007	0.017	–0.013	–0.025	–0.001
Medium (2nd/3rd quartiles) (ref.)									
High (4th quartile)	0.005	–0.008	0.018	0.004	–0.010	0.017	0.008	–0.006	0.022
Population density per km²									
Less than 50 (ref.)									
50–100	–0.021	–0.039	–0.004	–0.035	–0.054	–0.016	–0.041	–0.062	–0.021
100–1,000	–0.021	–0.057	0.016	–0.032	–0.067	0.002	–0.041	–0.075	–0.007
More than 1,000	–0.088	–0.196	0.020	–0.114	–0.228	–0.001	–0.007	–0.115	0.101
Regional dummies									
Less than 10 km from Stockholm (ref.)									
10–50 km from Stockholm	0.043	–0.120	0.206	0.078	–0.092	0.248	0.207	0.029	0.385
50–100 km from Stockholm	0.080	–0.090	0.250	0.062	–0.115	0.238	0.224	0.039	0.410
100–150 km from Stockholm	0.103	–0.069	0.275	0.092	–0.087	0.271	0.243	0.055	0.431
150–200 km from Stockholm	0.168	–0.005	0.341	0.158	–0.021	0.338	0.299	0.110	0.488
Less than 10 km from Gothenburg	0.241	0.051	0.430	0.257	0.053	0.461	0.393	0.164	0.621
10–50 km from Gothenburg	0.286	0.110	0.461	0.285	0.102	0.468	0.400	0.204	0.595
50–100 km from Gothenburg	0.273	0.100	0.447	0.253	0.072	0.434	0.374	0.182	0.567
Less than 10 km from Malmö	0.175	–0.016	0.367	0.075	–0.128	0.278	0.376	0.146	0.605
10–50 km from Malmö	0.162	–0.016	0.339	0.149	–0.036	0.335	0.357	0.154	0.561
50–100 km from Malmö	0.178	0.002	0.354	0.172	–0.012	0.355	0.340	0.142	0.538
Gotland	–0.089	–0.262	0.084	–0.094	–0.273	0.086	0.087	–0.102	0.277
Southern Norrland & Kopparberg county	0.195	0.021	0.368	0.170	–0.011	0.350	0.320	0.126	0.515
Northern Norrland	0.391	0.211	0.571	0.335	0.149	0.522	0.373	0.166	0.580
Other areas (central & southern Sweden)	0.247	0.074	0.419	0.210	0.031	0.390	0.347	0.157	0.536
Constant	1.031	0.859	1.202	1.058	0.880	1.237	0.924	0.737	1.112
Number of Women	239,268			220,105			200,589		
Number of Parishes	2,422			2,426			2,428		

Table OA16 (continued)

Workers and Others

	1880 Posterior Marginals			1890 Posterior Marginals			1900 Posterior Marginals		
	Mean	0.025	0.975	Mean	0.025	0.975	Mean	0.025	0.975
Individual-Level Covariates									
Age of woman									
15–19 years	–0.543	–0.582	–0.504	–0.541	–0.584	–0.498	–0.445	–0.484	–0.407
20–24 years	–0.205	–0.218	–0.193	–0.143	–0.158	–0.128	–0.076	–0.089	–0.062
25–29 years	0.043	0.033	0.053	0.072	0.061	0.083	0.125	0.115	0.136
30–34 years (ref.)									
35–39 years	–0.191	–0.200	–0.181	–0.207	–0.217	–0.196	–0.217	–0.228	–0.207
40–44 years	–0.552	–0.562	–0.542	–0.563	–0.574	–0.552	–0.556	–0.566	–0.545
45–49 years	–1.095	–1.105	–1.084	–1.121	–1.132	–1.110	–1.080	–1.091	–1.069
50–54 years	–1.379	–1.390	–1.369	–1.383	–1.395	–1.371	–1.328	–1.339	–1.316
Age difference between spouses									
Wife older	0.021	0.013	0.029	0.016	0.007	0.024	0.038	0.030	0.046
Husband 0–2 years older (ref.)									
Husband 3–6 years older	–0.010	–0.019	–0.002	–0.022	–0.031	–0.013	–0.008	–0.017	0.000
Husband >6 years older	–0.078	–0.087	–0.070	–0.096	–0.106	–0.087	–0.067	–0.076	–0.058
Children >4 years old in household									
No (ref.)									
Yes	0.250	0.243	0.258	0.274	0.266	0.282	0.262	0.254	0.269
Husband household head									
Yes (ref.)									
No	–0.186	–0.198	–0.175	–0.196	–0.210	–0.182	–0.200	–0.215	–0.185
Distance from parish of birth									
Less than 10 km (ref.)									
10–50 km	0.018	0.011	0.025	0.024	0.017	0.032	0.022	0.015	0.030
More than 50 km	0.046	0.037	0.055	0.039	0.030	0.049	0.021	0.012	0.029
Born abroad	–0.003	–0.041	0.034	0.010	–0.026	0.045	–0.019	–0.050	0.012
Parish-Level Covariates									
Female labor force rate									
Low (1st quartile)	–0.007	–0.018	0.004	–0.003	–0.014	0.009	0.005	–0.006	0.016
Medium (2nd/3rd quartiles) (ref.)									
High (4th quartile)	–0.006	–0.016	0.005	–0.008	–0.020	0.005	–0.008	–0.020	0.003

Education rate (teacher/child ratio)									
Low (1st quartile)	–0.008	–0.018	0.001	0.008	–0.003	0.018	0.007	–0.004	0.017
Medium (2nd/3rd quartiles) (ref.)									
High (4th quartile)	–0.006	–0.016	0.003	–0.004	–0.015	0.007	–0.018	–0.030	–0.007
Proportion employed in industry									
Low (1st quartile)	0.007	–0.005	0.019	0.012	–0.002	0.026	0.006	–0.007	0.020
Medium (2nd/3rd quartiles) (ref.)									
High (4th quartile)	0.004	–0.006	0.014	–0.004	–0.015	0.007	–0.008	–0.018	0.003
Proportion of migrants born more than 100 km away and/or abroad									
Low (1st quartile)	0.000	–0.012	0.012	–0.007	–0.021	0.008	0.007	–0.007	0.022
Medium (2nd/3rd quartiles) (ref.)									
High (4th quartile)	–0.001	–0.012	0.010	–0.007	–0.019	0.005	–0.021	–0.032	–0.010
Population density per km²									
Less than 50 (ref.)									
50–100	–0.026	–0.041	–0.010	–0.007	–0.024	0.010	–0.027	–0.043	–0.012
100–1,000	–0.057	–0.075	–0.038	–0.045	–0.064	–0.026	–0.056	–0.073	–0.040
More than 1,000	–0.077	–0.112	–0.042	–0.077	–0.112	–0.043	–0.113	–0.143	–0.083
Regional dummies									
Less than 10 km from Stockholm (ref.)									
10–50 km from Stockholm	0.064	–0.013	0.141	0.006	–0.076	0.088	0.049	–0.021	0.120
50–100 km from Stockholm	0.029	–0.056	0.115	–0.040	–0.131	0.052	0.037	–0.042	0.117
100–150 km from Stockholm	0.056	–0.032	0.144	–0.021	–0.115	0.073	0.057	–0.026	0.139
150–200 km from Stockholm	0.091	0.002	0.180	0.006	–0.089	0.102	0.083	–0.001	0.167
Less than 10 km from Gothenburg	0.183	0.066	0.301	0.104	–0.023	0.230	0.224	0.112	0.336
10–50 km from Gothenburg	0.160	0.064	0.257	0.082	–0.023	0.186	0.159	0.066	0.252
50–100 km from Gothenburg	0.157	0.063	0.250	0.059	–0.042	0.160	0.171	0.081	0.260
Less than 10 km from Malmö	0.233	0.104	0.363	0.106	–0.033	0.245	0.162	0.040	0.285
10–50 km from Malmö	0.173	0.066	0.280	0.028	–0.088	0.144	0.120	0.017	0.223
50–100 km from Malmö	0.179	0.079	0.280	0.026	–0.083	0.136	0.099	0.003	0.197
Gotland	–0.080	–0.170	0.010	–0.150	–0.249	–0.052	–0.033	–0.121	0.055
Southern Norrland & Kopparberg county	0.103	0.008	0.198	0.038	–0.063	0.140	0.113	0.024	0.202
Northern Norrland	0.117	0.001	0.233	0.003	–0.120	0.126	0.151	0.043	0.259
Other areas (central & southern Sweden)	0.133	0.044	0.222	0.038	–0.058	0.133	0.142	0.057	0.226
Constant	1.111	1.024	1.198	1.208	1.114	1.301	1.082	0.999	1.164
Number of Women	282,534			296,842			331,914		
Number of Parishes	2,435			2,435			2,435		

Sources: Swedish National Archives et al. (2011a, 2011b, 2014), own calculations.

7 Lifetime Net Migration Patterns by Cohort

In this section, we present background information on lifetime net migration patterns[1] by cohort, as we derived them from the censuses of 1880, 1890, and 1900. We are mostly interested in comparing patterns for the same cohort in different censuses. How did, for example, the lifetime net migration patterns shift for the cohort born in 1860 between age 30 (as captured in the 1890 census) and age 40 (as obtained in the 1900 census)? We are particularly interested in knowing to what degree we still observe changes at ages between 30 and 50, which was the age range in which most of the fertility decline occurred during our observation period. If most shifts in lifetime net migration patterns were registered before age 30, this could be interpreted as an indication that the causal direction of the statistical association between migration background attributes and fertility outcomes is rather from the former to the latter. The outcomes are presented in Figure OA6 (all SES groups) and Figure OA7 (by SES). For the graphs that display the patterns for all SES groups, we are not reliant on SES information from the husband. Thus, we can plot in Figure OA6 the patterns not only for our datasets of married women (black lines) but also for the whole female population of Sweden independent of the marital status (gray lines).

A comparison of the lifetime net migration patterns of specific cohorts across censuses suggests that at ages between 20 and 30, the share of women who lived farther away from their birth parish was still increasing. In the subplots for all social groups (Figure OA6), we see that the patterns for married women and for all women (as depicted by the black and gray lines) are closely aligned, especially among the internal long-distance migrants who are living more than 50 km away from their birth parish. This finding provides support for the view that the increases in the share of such migrants among the married women between ages 20 and 30 in our sample are not just artifacts of selection effects. The latter could occur if people who migrated married later. After age 30, the patterns become particularly stable for the elite. For example, among the elite women born in 1850, the share of women living within 10 km around the parish of birth was app. 37% in all three censuses (1880: age 30; 1890: age 40; 1900: age 50). The patterns for all women, farmers, and workers and others were not as stable as those for the elite. However, no substantial changes occurred after age 30 among these groups, except perhaps among the workers and others.

---

[1] As in our paper, we obtained these data for individuals born within Sweden as the spherical distance between the parish of birth and the current parish of residence; while individuals born abroad are placed in their own category. We consider the same categorization of the lifetime net migration distances as in our models.

Thus, if high levels of migration were still occurring at ages 30 and older among important forerunners of the fertility decline like the elite, long-distance movements further away from the birth parish must have been offset by equal shares of long-distance migrants moving back to the vicinity of their birth parishes. However, considering that emigration by age, for which we have data available, was substantially decreasing after age 30 (see Table OA2 of section 3 of the online appendix) we consider such a scenario rather unlikely. We certainly cannot rule out the possibility that people who lived farther away from their birth place might have been more likely to have moved again after age 30, which could have had disruptive effects on their fertility schedules. It is, however, likely that the first move that selected these individuals into being a long-distance migrant occurred before age 30.

The graphs in Figure OA6 also show that a non-negligible share of the migrants left the area around their birth parish as children. It is a limitation that our data do not allow us to distinguish between these migrants and the bigger group of migrants who left the area around their birth parish after reaching adulthood. Overall, however, these cautious and tentative assessments suggest that in many life courses the selection into being a person who lives far away from the birth parish is likely to have occurred prior to the decision to adopt a fertility-controlling behavior. Nevertheless, our datasets do not allow us to conclusively determine whether this was the case. Thus, future research should reexamine this issue using data containing information on the order of these events. The quite smooth patterns over age shown in Figure OA6 and Figure OA7 also provide support for the view that the information on the parish of birth provided in the Swedish censuses of that time was quite reliable.

Fig. OA6 Lifetime net migration patterns for cohorts in 1880, 1890, and 1900

All SES (black lines: our sample of married women; gray lines: all women):

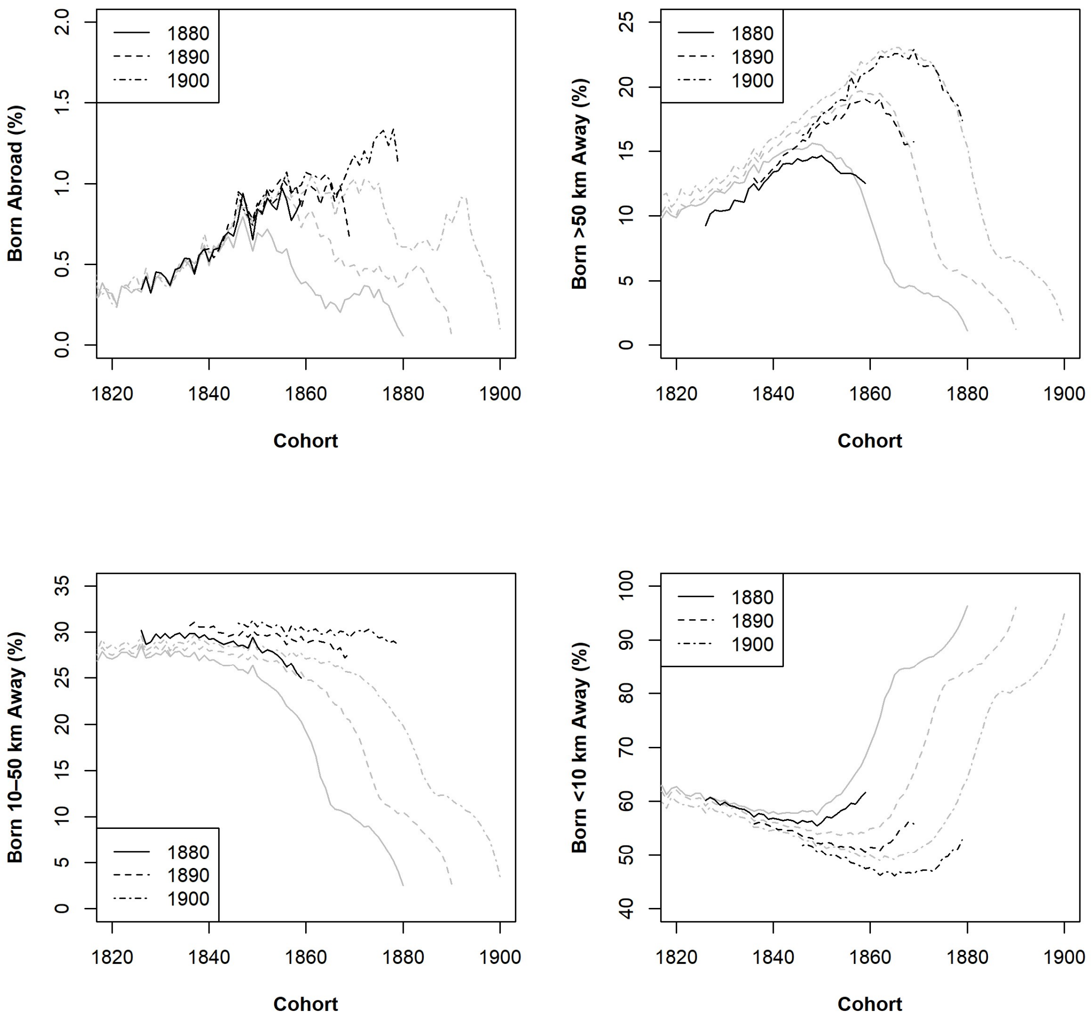

Note: The black lines show the values for our sample of women used in the analysis, the gray lines the patterns for all women independent of marital status and presence of the husband.

Sources: Swedish National Archives et al. (2011a, 2011b, 2014), own calculations.

Fig. OA7 Lifetime net migration patterns for cohorts by SES in our sample in 1880, 1890, and 1900

a) Elite:

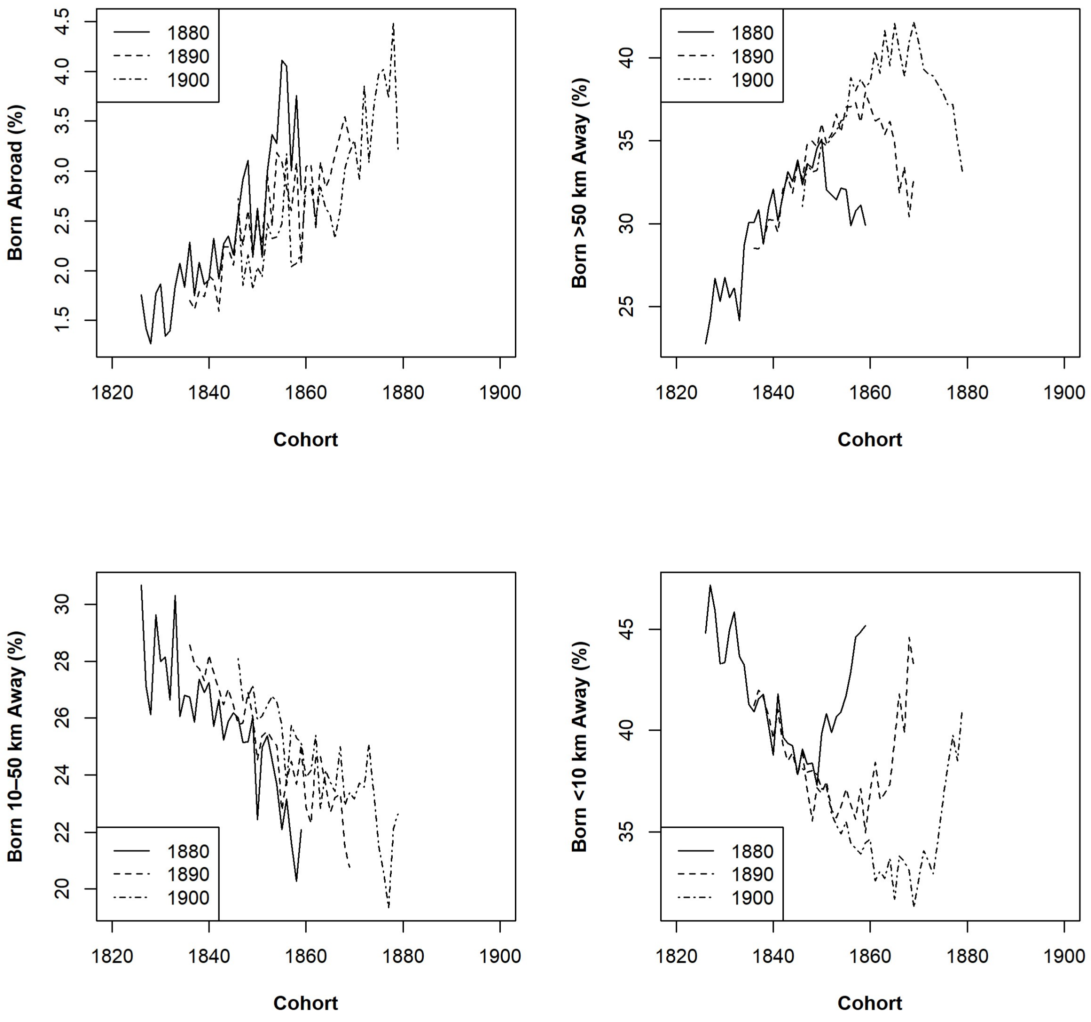

Fig. OA7 (continued)

b) Farmers:

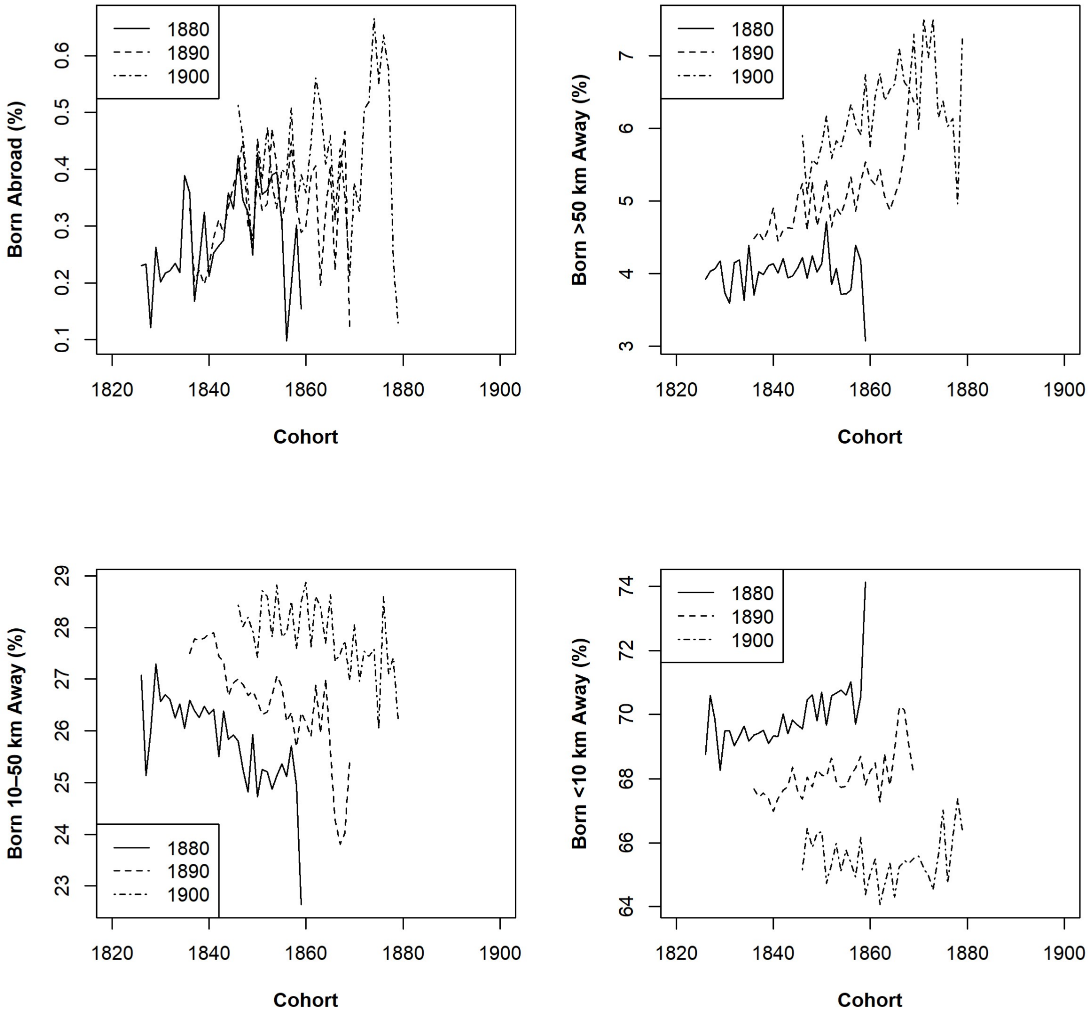

Fig. OA7 (continued)

c) Workers and Others:

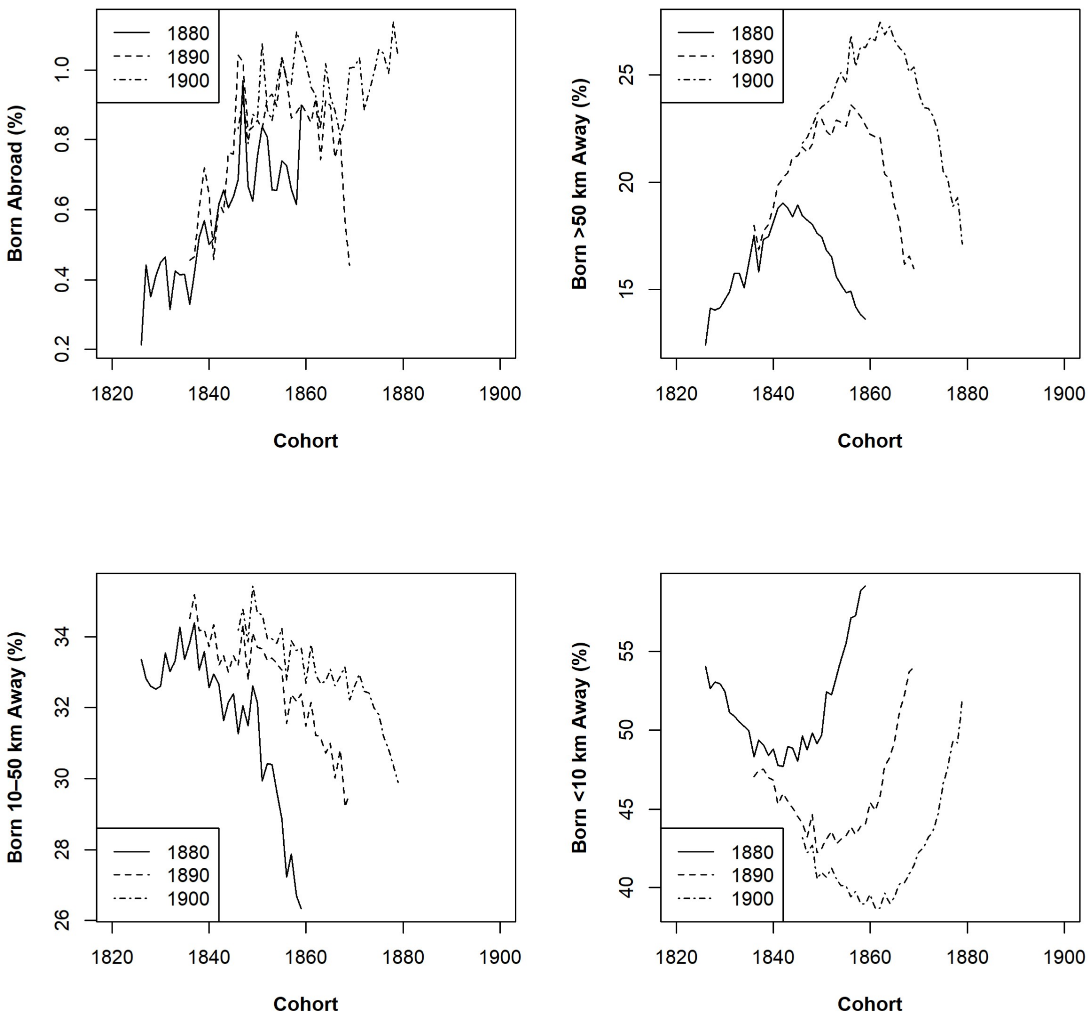

Sources: Swedish National Archives et al. (2011a, 2011b, 2014), own calculations.

References

Besag, J., York, J., & Mollié, A. (1991). Bayesian image restoration, with two applications in spatial statistics. *Annals of the Institute of Statistical Mathematics, 43,* 1–20.

Bivand, R., Gómez-Rubio, V., & Rue, H. (2015). Spatial data analysis with R-INLA with some extensions. *Journal of Statistical Software, 63* (i20). https://doi.org/10.18637/jss.v063.i20

Bohlin, J., & Eurenius, A. M. (2010). Why they moved: Emigration from the Swedish countryside to the United States, 1881–1910. *Explorations in Economic History, 47,* 533–551.

Coale, A. J., & Treadway, R. (1986). A summary of the changing distribution of overall fertility, marital fertility, and the proportion married in the provinces of Europe. In A. J. Coale, & S. C. Watkins (Eds.), *The decline of Fertility in Europe: The revised proceedings of a conference on the Princeton European Fertility Project* (pp. 31–181). Princeton, NJ: Princeton University Press.

Coale, A. J., & Watkins, S. C. (Eds.). (1986). *The decline of fertility in Europe: The revised proceedings of a conference on the Princeton European Fertility Project*. Princeton, NJ: Princeton University Press.

Dribe, M. (2009). Demand and supply factors in the fertility transition: A county-level analysis of age-specific marital fertility in Sweden, 1880–1930. *European Review of Economic History, 13,* 65–94.

Dribe, M., & Scalone, F. (2014). Social class and net fertility before, during, and after the demographic transition: A micro-level analysis of Sweden 1880–1970. *Demographic Research, 30*(article 15), 429–464. https://doi.org/10.4054/DemRes.2014.30.15

Human Mortality Database (2017). The Human Mortality Database. Berkeley/Rostock: University of California, Berkeley, CA/Max Planck Institute for Demographic Research, Rostock, Germany. Available at: http://www.mortality.org/

Martins, T. G., Simpson, D., Lindgren, F., & Rue, H. (2013). Bayesian computing with INLA: New features. *Computational Statistics & Data Analysis, 67,* 68–83.

Max Planck Institute for Demographic Research (MPIDR) (2014). MPIDR Population History GIS collectioC. Rostock, Germany: MPIDR. Retrieved from https://censusmosaic.demog.berkeley.edu/data/historical-gisfiles

Riebler, A., Sørbye, S. H., Simpson, D., & Rue, H. (2016). An intuitive Bayesian spatial model for disease mapping that accounts for scaling. *Statistical Methods in Medical Research, 25,* 1145–1165.

Riksarkivet. (2016). Historiska GIS-kartor (information om territoriella indelningar i Sverige från 1500-talets slut till 1900-talets slut) [Historical GIS map information on the administrative division of Sweden from the 16th to the 20th century]. Stockholm, Sweden: Riksarkivet.

Scalone, F., & Dribe, M. (2017). Testing child-woman ratios and the own-children method on the 1900 Sweden census: Examples of indirect fertility estimates by socioeconomic status in a historical population. *Historical Methods, 50,* 16–29.

Shyrock, H. S., & Siegel, J. S. (1980). *The methods and materials of demography* (vol. 2). Washington, DC: U.S. Department of Commerce, Bureau of Census.

Statistics Sweden. (1999). *Befolkningsutvecklingen under 250 år: Historisk statistik för Sverige* [Population development in Sweden over 250 years: Swedish historical statistics]. Stockholm: Statistics Sweden.

Swedish National Archives, Umeå University, & Minnesota Population Center. (2011a). National sample of the 1890 census of Sweden: version 2.0. Minneapolis, MN: Minnesota Population Center [distributor].

Swedish National Archives, Umeå University, & Minnesota Population Center. (2011b). National sample of the 1900 census of Sweden: version 3.0. Minneapolis, MN: Minnesota Population Center [distributor].

Swedish National Archives, Umeå University, & Minnesota Population Center. (2014). National sample of the 1880 census of Sweden: version 2.0. Minneapolis, MN: Minnesota Population Center [distributor].
